# Meta-analytic evidence for distinct neural correlates of conditioned versus verbally induced placebo analgesia

**DOI:** 10.1038/s41467-026-74743-0

**Published:** 2026-07-17

**Authors:** Tamas Spisak, Helena Hartmann, Matthias Zunhammer, Balint Kincses, Katja Wiech, Tor D. Wager, Ulrike Bingel

**Affiliations:** 1 Center for Translational Neuro- and Behavioral Sciences (C-TNBS), University Hospital Essen, Essen, Germany; 2 Department of Neurology, University Hospital Essen, Essen, Germany; 3 Wellcome Centre for Integrative Neuroimaging (WIN), Nuffield Department of Clinical Neurosciences, University of Oxford, Oxford, UK; 4 Department of Psychological and Brain Sciences, Dartmouth College, Hanover, NH USA; 5 National Center for Complementary and Integrative Health and National Institute of Mental Health, National Institutes of Health, Bethesda, MD USA; 6 National Institute on Drug Abuse, National Institutes of Health, Baltimore, MD USA; 7 University of Turin, Turin, Italy; 8 Plateau Rosà Labs, Plateau Rosà, Switzerland; 9 University Medical Center Hamburg–Eppendorf, Hamburg, Germany; 10 Graduate School of Seoul Cyber University, Seoul, South Korea; 11 Yonsei University, Wonju College of Medicine, Wonju, South Korea; 12 Cham Psychology Brain Health Institute, Seoul, South Korea; 13 University of Maryland, Baltimore, MD USA; 14 Università di Bologna, Bologna, Italy; 15 Max Planck Institute for Human Cognitive and Brain Sciences, Leipzig, Germany; 16 Department of Physics and Computation al Radiology, Division of Radiology and Nuclear Medicine, Oslo University Hospital, Oslo, Norway; 17 School of Health Sciences, Kristiania University of Applied Sciences, Oslo, Norway; 18 Department of Medical Psychology and Medical Sociology, Ruhr University Bochum, Bochum, Germany; 19 Johns Hopkins University, Baltimore, MD USA; 20 Beth Israel Deaconess Medical, Harvard Medical School, Boston, MA USA; 21 Harvard Medical School, Cambridge, MA USA; 22 Massachusetts General Hospital, Harvard Medical School, Cambridge, MA USA; 23 University of Oslo, Oslo, Norway; 24 University of Modena e Reggio Emilia, Modena, Italy; 25 University of Vienna, Vienna, Austria; 26 University of Oxford, Oxford, United Kingdom; 27 Karolinska Institute, Solna, Sweden; 28 Wake Forest School of Medicine, Winston-Salem, NC USA

**Keywords:** Perception, Prefrontal cortex, Sensory processing, Classical conditioning

## Abstract

Placebo analgesia demonstrates that belief and expectation can significantly alter pain, even without active treatment. Placebo analgesia can be induced through verbal suggestion, classical conditioning, or their combination, though the role of conditioned neural responses above and beyond effects of verbal instructions remains unclear. We conduct a systematic meta-analysis of individual participant data from 16 within-participant placebo neuroimaging studies (*n* = 409), employing univariate and multivariate analyses to identify shared and distinct mechanisms of placebo analgesia induced by suggestions alone versus suggestions combined with conditioning. Both techniques increase activity during pain in the dorsolateral prefrontal and inferior parietal cortices and decrease activation in the insula, putamen, and primary sensory areas. Adding conditioning enhances engagement of regions associated with context representation and pain modulation (e.g., dorsolateral/dorsomedial prefrontal cortices) and decreases in nociceptive regions (e.g., primary sensory and insular areas). Conditioning also strengthens the negative association between analgesia and nociceptive activity, as quantified by the Neurologic Pain Signature. Combining conditioning with instructions yields greater placebo analgesia, mediated by increased ventromedial prefrontal and dorsal caudate activity, alongside decreased sensory-nociceptive and cerebellar activity. These findings suggest the two strategies rely on partially distinct mechanisms in the brain.

## Abstract (web summary)

Placebo analgesia shows that expectations induced by suggestions or conditioning can reduce pain without active treatment. Here, the authors conduct an individual-level meta-analysis of 16 studies and show that placebo analgesia induced by these different induction techniques is mediated by partly distinct brain mechanisms.

## Introduction

Placebo effects play a significant role in health and treatment outcomes in both experimental and clinical research. Understanding the underlying mechanisms of these effects is crucial for optimizing drug development and clinical care. Placebo analgesia (PA), the relief of pain by sham treatments—like inert pills, creams, and injections—is the best-studied example of placebo effects. Placebo analgesia is typically induced through (a) verbal suggestions from an experimenter or physician that promote positive expectations, and (b) classical conditioning techniques that offer direct experiences of pain relief by experimentally modifying nociceptive input^1,2^. Most commonly, a combination of these two methods is employed, which has been shown to enhance the effectiveness of placebo treatments^1,3^.

Extensive neuroscientific research over the past few decades has demonstrated that placebo analgesia (PA) is a complex biopsychosocial phenomenon, involving distinct central nervous system mechanisms that affect pain perception^2,4^. Subjective changes in pain perception have been associated with altered processing of pain and the involvement of top-down pain modulatory activity including the release of endogenous opioids^5,6^ and other neurotransmitters (e.g., dopamine;^7^). More specifically, PA has been linked to the engagement of the dorsolateral and ventromedial prefrontal cortices (DLPFC and VMPFC), anterior cingulate cortex (ACC) and subcortical structures such as the hypothalamus, amygdala and the periaqueductal grey (PAG)^8–10^. Spinal cord imaging studies suggest that the influence of this top-down modulation can even extend to the spinal level, leading to observable alterations in nociceptive processing^11,12^. Our two recent systematic individual participant level meta-analyses of 20 independent neuroimaging studies found placebo-related activity increases consistent with modulatory activity in fronto-parietal regions, and decreases in the insula, thalamus and cerebellum. However, the effect on the Neurologic Pain Signature (NPS), an established brain signature of pain processing, was negligible in size^13–15^. These results therefore suggest that multiple pathways and mechanisms contribute to PA and largely bypass classical ascending nociceptive pathways.

At present, the question of whether verbal instruction and conditioning, as the two main methods of inducing PA, rely on the same neural pathways remains unanswered. Involvement of the descending pain modulatory system, opioid release and altered spinal cord processing have so far only been documented in studies with a conditioning component^5,6,12,16^. Whether this is the result of selective reporting and publication bias, or whether it actually reflects different mechanisms of conditioned and verbally induced PA, remains to be investigated. Evidence from studies that consider the impact of these two induction methods on dynamic learning in the context of fear, reward, and pain suggests that the neural underpinnings of these processes may indeed differ, and that they can interact when combined, such that instructions shape brain responses during learning, particularly in the striatum and ventromedial prefrontal cortex^17–21^. Behavioral studies indicate that conditioning enhances instruction-based placebo effects on pain^3,22^ and event-related potentials^23^ and creates placebo effects that are more resistant to later extinction and suggestions^24,25^. However, to date, neuroimaging studies of placebo analgesia have not directly compared the effects of instructions alone and instructions paired with classical conditioning, in part because of the large sample sizes required to do so with sufficient power. Meta-analyses provide a valuable approach to fill this gap, as they offer a quantitative synthesis and comparison of effect sizes across multiple studies, thereby enhancing power and generalizability of findings across studies.

Here, we perform an individual participant data meta-analysis (‘mega-analysis’), to disentangle the neural mechanisms of placebo induced by instructions alone or in combination with conditioning. We analyzed person-level data from 16 within-participant functional neuroimaging studies collected as part of the Placebo Imaging Consortium^15^ which included behavioral and brain responses during evoked pain with and without placebo treatment. In these studies, PA was induced either by verbal instructions alone (INST) or by verbal instructions combined with conditioning (COND-INST).

First, we tested the hypothesis that combining instructions with conditioning (as compared to instructions only) indeed results in stronger behavioral PA. Second, we identified the brain regions engaged by both induction strategies and those showing differential placebo-related activity with conditioning. Third, we performed a mediation analysis to test whether differential activity in these regions mediates the effect of induction type on PA. Finally, we investigate differences between induction types and two established and validated brain signatures of pain, the Neurologic Pain Signature reflecting stimulus input dependent pain (NPS;^13^) and the Stimulus Intensity Independent Pain Signature (SIIPS;^26^).

## Results

We analyzed participant-level data from a subset of studies included in previous meta-analyses of placebo effects on the Neurologic Pain Signature (NPS)^14^ and across the entire brain^15^. Here, we included only within-participant studies, where person-level functional magnetic resonance imaging (fMRI) maps and pain ratings were available in both the placebo and the control conditions, allowing us to account for the strength of pain experience in the control condition and the magnitude of PA at the individual-person level. This resulted in a dataset with *k* = 16 studies and *n* = 415 participants (*n* = 409 after excluding participants with missing pain ratings). The included studies induced PA either by verbal instructions alone (INST; *k* = 5, *n* = 147) or by a combination of verbal instructions and conditioning (COND + INST; *k* = 11, *n* = 268, see Supplementary Tables S1 and S2 and Supplementary Figs. S1-4). A detailed description of the data collection procedures can be found in ref. ^14^. In brief, we performed a systematic literature search to identify experimental fMRI studies of PA. Eligible studies met the following criteria: (a) published in English in a peer-reviewed journal; (b) constituted an empirical investigation; (c) involved human participants; (d) employed functional neuroimaging of the brain during evoked pain; and (e) induced pain under matched placebo and control conditions. The authors of all identified studies were contacted and invited to share their data. We collected single-participant, first-level, whole-brain standard space summary images of pain response (statistical parametric maps) from the original analyses, corresponding pain ratings separately for placebo and control conditions, experimental design parameters, and demographic data.

We performed a dedicated risk-of-bias assessment with the Cochrane risk of bias tool^27^, evaluating the risk for selection, performance, detection, attrition and reporting and sequence biases. Our assessment did not find evidence for publication bias (see Supplementary Fig. S11) and concluded that, in terms of behavioral and neural differences between the induction types, the risks of these biases are generally low, although they cannot be fully ruled out. For details see Supplementary Methods 1-3, Supplementary Tables S3-4 and Supplementary Fig. S5.

**Fig. 1:**
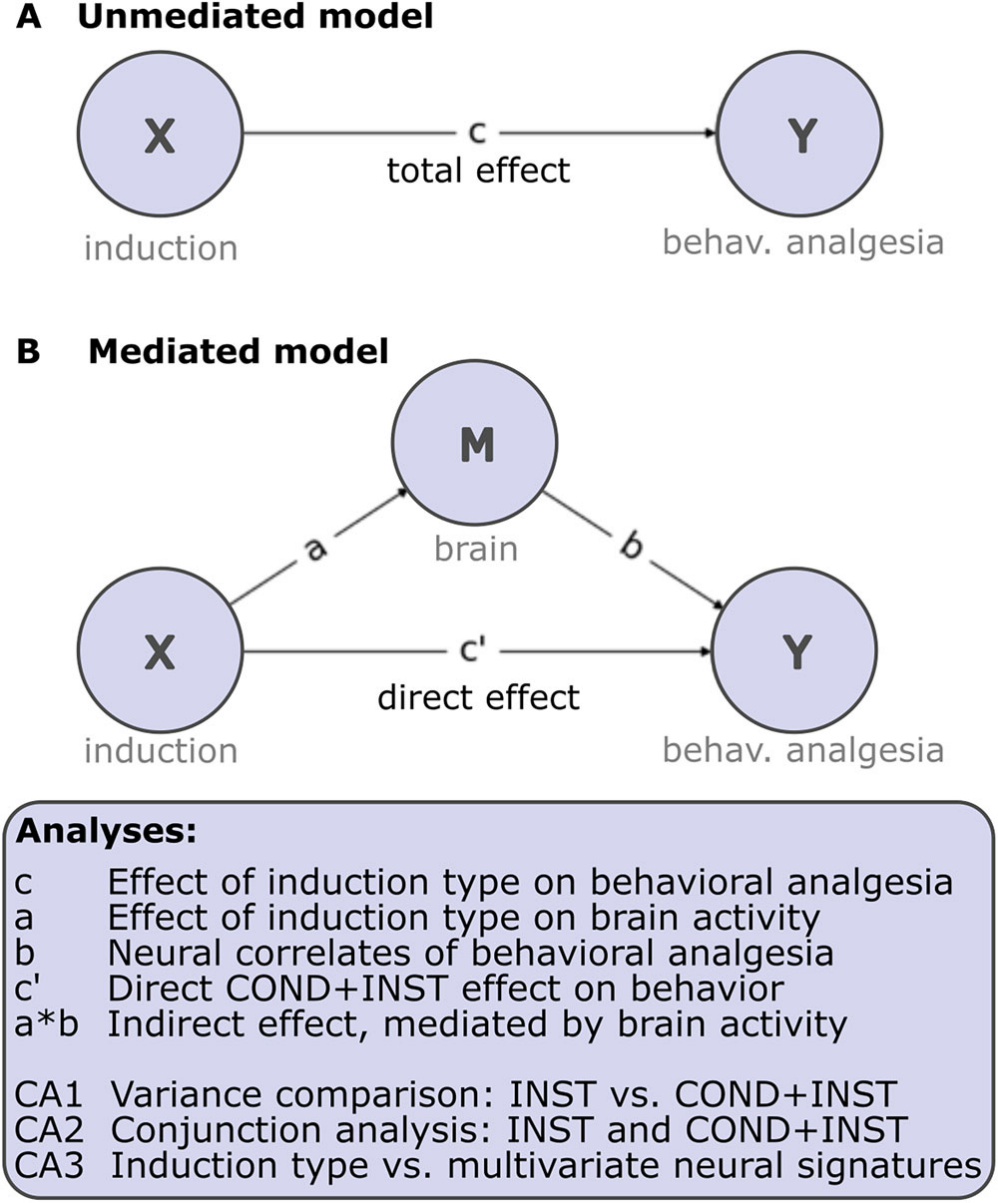
Analyses performed in the present manuscript. We fit an unmediated (**A**) and a mediated (**B**) model on placebo induction technique (X: INST or COND + INST), behavioral analgesia (Y: control-placebo rating difference on VAS 0-100) and the quantile-harmonized brain placebo response data (M: control-placebo contrast maps). This results in analyses quantifying the effect: c: induction type on behavioral analgesia, a induction type on brain activity, b: brain activity on behavioral analgesia, as well as the direct (c’) and indirect or mediated (a*b) effects. CA1: We compared the variances in the unharmonized behavioral placebo responses between INST and COND + INST; CA2: Complementary conjunction analysis to identify brain regions activating in both induction types. CA3: Effect of induction type on two multivariate neural signatures: the Neurologic Pain Signature (NPS) and the Stimulus Intensity-Independent Pain Signature (SIIPS).

To address heterogeneity between studies, we harmonized the brain data (control –placebo contrast images) by converting the values to their quantile distance from zero, independently for each voxel within each study (see Methods for details). We then examined the total effect of induction type (*X*) on behavioral analgesia (*Y*), referred to as the “total effect” (path c, Fig. 1A). Next, we conducted a mediation analysis (Fig. 1B) to identify the brain mediators (*M*) underlying this association. In our mediation framework, path a corresponds to the effect of induction type on brain placebo responses (*X* → *M*), and path b corresponds to the correlation between placebo-related brain activity and behavioral PA (*M* → *Y*). We evaluated the mediation effect a*b, i.e. the joint effects of induction type on brain placebo response (path a) and brain placebo response on behavioral PA (path b). We included age, sex and pain ratings in the control condition as covariates and modeled studies with dummy regressors orthogonalized to induction type.

We performed three further analyses (CA1-3), complementary to the brain mediation analyses: (i) we compared the variances in the unharmonized placebo responses across INST and COND + INST studies (accounting for age, sex and baseline pain rating); (ii) we conducted a conjunction analysis to identify brain regions that are involved both in INST and COND + INST and (iii) extracted the NPS and SIIPS scores independently for all participants and analyzed whether induction type has an effect on their association with behavioral PA.

### Path c: Behavioral results

**Fig. 2:**
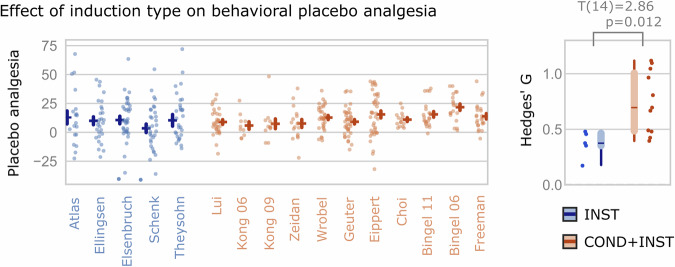
Applying conditioning together with verbal instructions results in stronger behavioral placebo effects than verbal instructions alone. Placebo analgesic effects in studies using verbal instruction alone (INST, shown in blue) and studies using a combination of verbal instructions and conditioning (COND + INST, shown in orange). On the left side, the y-axis shows the difference in the 0–100 VAS pain intensity rating between the placebo condition and the control condition. Greater values indicate a stronger behavioral placebo analgesia. Points indicate individual participants’ effects. Mean and standard error of the ratings are depicted by line plots. The standardized effect size difference is 0.377 (Hedges’ G, 95% CI = [0.0946, 0.6587]) which is statistically significant with t(14) = 2.86 and *p* = 0.006, one-tailed. For results corrected for age, sex and pain rating in the control condition, see Supplementary Table S5. On the right side, normalized effect size of rating scores (Hedge’s g) for each study, pooled by study type. A significant difference (t-test, t(14) = 2.86; *p* = 0.012 (two-tailed); k = 16 studies (including *n* = 409 participants)) in the pooled effect sizes indicates stronger placebo analgesia in the COND + INST conditions. Box-plots show the median (centre line), the interquartile range (25th–75th percentiles; box). Whiskers extend to the minimum and maximum values within 1.5× the interquartile range. All individual data points are visualized.

Mean ( ± standard error) PA was 9.2 ( ± 1.83) and 12.2 ( ± 0.82) points on a 0-100 visual analogue scale (VAS), respectively, indicating that combining instructions with conditioning led to an additional pain reduction of 3 points, on average (as compared to verbal instructions alone, see Fig. 2). These absolute effect size translated to a mean ( ± standard deviation across studies) standardized Hedges’ *g* effect of 0.367 ( ± 0.121) and 0.744 ( ± 0.278) for INST and COND + INST studies, indicating ‘small-to-moderate’ and ‘moderate-to-large’ effects, respectively. The difference 0.377 ( ± 0.303 was found to be statistically significant (*T*(14) = 2.86, *p* = 0.006, one-tailed). When corrected for age, sex, pain ratings in the control condition, and inter-study differences, the absolute effect size decreased to 1.34 VAS-points (*p* = 0.049, one-tailed; 95% CI: −0.247, 2.926). In the same model, we found that PA was overall significant (11.15 points on the VAS pooled across induction types; *p* < 0.001; 95% CI: 9.62, 12.67) and it varied significantly across studies (*F*(14) = 2.44, *p* = 0.003). Furthermore, we found that PA was statistically significantly associated with the reported pain intensity in the control conditions (β = 0.30 points more PA with every one-point increase in control pain; *p* < 0.0001, 95% CI: 0.22, 0.39) and it was weakly but statistically significantly correlated with age (β = 0.29 points less PA with every additional year; *p* = 0.048; 95% CI: −0.58, −0.002). See Supplementary Table S5 for details.

In our complementary analysis CA1, we tested whether the variances in the unharmonized placebo ratings are different with INST and COND + INST. First, we fit a mixed-effect model to explain the behavioral placebo effect with study as random effect and age, sex and control pain rating as fixed effects (see results in Supplementary Table S6). We then compared the residual variance between INST and COND + INST studies with Levene’s test and found that the variances of behavioral placebo effects with the two placebo induction types were significantly different (residual variances 393.4 and 155.1, for INST and COND + INST respectively; Levene’s test statistic: 27.2257, *p* < 0.001, df_1_ = 1, df_2_ = 407, Δσ_2_ = 238.3).

### Path a: effect of induction type on brain activity

**Fig. 3:**
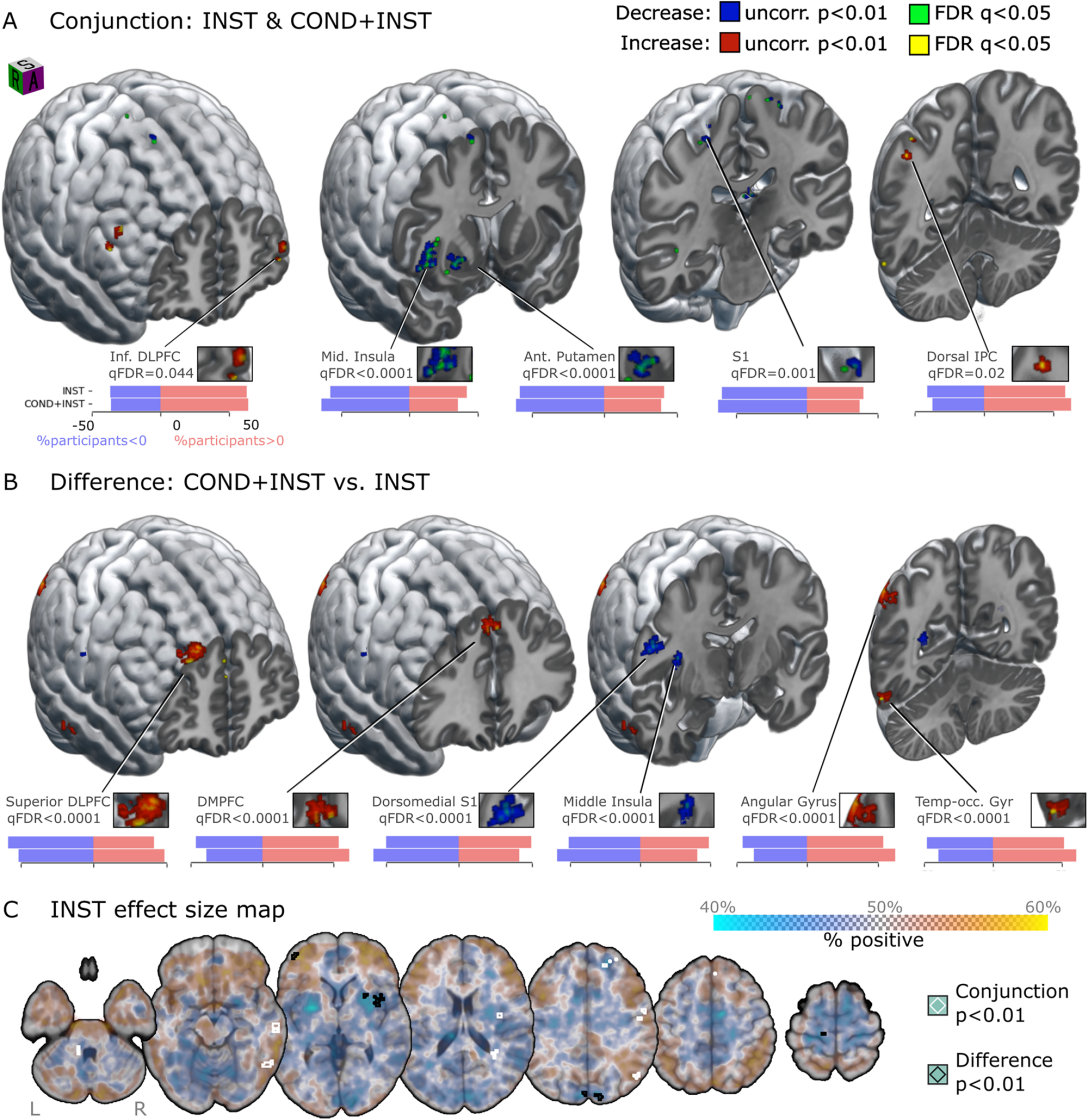
Path a: Neural correlates of placebo analgesia induction types. **A** Both INST and COND + INST were characterized by activations in the DLPFC and the inferior parietal cortex and deactivations in the putamen, insula and the isthmus cingulate cortex indicated by our conjunction analysis (one sided). The proportion of participants with positive (red) and negative (blue) activations in the INST and COND + INST studies is visualized by horizontal bars for selected regions. See Table 1 for a comprehensive list. **B** COND + INST resulted in significantly higher placebo-related activation in the middle temporal gyrus, DMPFC, DLPFC, dorsal inferior parietal cortex and decreased activation in middle insular and somatosensory areas as indicated by the voxelwise regression (path a in mediation analysis) of placebo induction type and brain response (two-sided). As in panel **A**, the proportion of participants with positive (red) and negative (blue) activations in the COND and COND + INST studies is visualized by horizontal bars for selected regions. See Table 2 for a comprehensive list. **C** Unthresholded effect size map in the INST-studies, overlayed with the contours of the conjunction (white) and the difference (black) maps. Due to our harmonization approach, effect size translates to the ratio of participants with positive activation as shown by the colorbar. The transparency of the color map for plotting the effect size is modulated by effect size magnitude, as shown on the colorbar (smaller effects are more transparent). The spatial orientation of the three-dimensional visualizations is denoted by a cube on panel **A**, with letters denoting the superior (S), right (R) and anterior (A) views. In the case of uncorrected results (*p* < 0.01), we only show clusters that contain at least one voxel surviving false discovery rate correction (q < 0.05). Path b: Correlation between placebo responses in the brain and behavioral analgesia.

First, we identified brain regions involved in PA both with INST and COND + INST with a statistical conjunction analysis (CA2). Our analysis revealed joint signal increases (Placebo > Control) in the DLPFC and the inferior parietal cortex (Fig. 3A, Table 1), all surviving FDR-correction (*q* < 0.05) for multiple comparisons. Joint FDR-significant decreases (Placebo <Control) were found in the putamen, insula and the cingulate isthmus. While the conjunction analysis is appropriate for identifying core regions commonly engaged across both induction methods, its statistical conservativeness may overlook meaningful activation patterns present in only one condition or marginally in both. Therefore, in a supplementary analysis (Supplementary Fig. S6 and Supplementary Table S7), we have evaluated the average response in all participants regardless of induction type (direct contrast), as well as the union of significant voxels (inclusive masking). This analysis largely corroborated our previously published results^15^ based on the restricted dataset used in the present study.

**Table 1:** Significant activations in the INST & COND + INST conjunction analysis

Cluster size (mm^3^)	X	Y	Z	%>0 INST	%>0 COND +INST	qFDR	Region	Network
**Placebo > Control (conjunction)**								
80	–44	50	2	63.1	64.0	0.04429	Left Rostral Inferior PLPFC	Cont
64	48	32	34	60.9	66.7	0.01053	Right Caudal DLPFC	Cont
32	54	30	22	59.7	63.4	0.00921	Right Caudal DLPFC	Cont
88	44	–52	42	58.9	62.9	0.01745	Right Dorsal Inferior Parietal Cortex	Cont
**Placebo < Control (conjunction)**								
40	26	12	2	38.6	37.7	0.00036	Right Putamen	N/A
56	22	–28	54	39.9	37.1	0.001	Right Dorsomedial Motor Cortex	SomMot
96	–6	–36	12	40.3	38.2	0.00569	Left Cingulate Isthmus	Cont
72	8	–86	34	40.3	36.4	0.03375	Right Cuneus	Vis
24	–26	–30	60	40.6	39.6	<0.0001	Left Dorsomedial S1	SomMot
24	–4	–86	34	40.8	37.7	0.03375	Left Cuneus	Vis
32	46	10	0	41.3	37.1	0.01839	Middle Insula	SalVentAtt
184	18	8	–4	41.3	39.0	< 0.0001	Right Putamen	N/A
40	–36	–8	30	41.3	38.2	0.02853	Left Middle Frontal Gyrus	Cont
24	18	–34	62	41.8	37.7	<0.0001	Right Dorsomedial S1	SomMot
24	6	–8	46	41.8	38.4	0.00137	Middle Cingulate Cortex	SalVentAttn
336	40	2	2	42.1	35.5	< 0.0001	Right Insular Cortex	SalVentAttn
24	10	8	64	42.3	40.0	0.03854	Right Dorsomedial Premotor Cortex	Cont

Only cluster peaks surviving FDR-correction, a cluster size ≥ 3 voxels (with *p <* 0.01) and a minimum distance of 20 mm to adjacent clusters are reported. X, Y and Z coordinates are reported in the MNI152 space, in units of mm. The percentages of participants with positive activation in the INST and COND* +* INST studies are shown in the columns “%>0 INST” and “%>0 COND* +* INST”, respectively (* <* 50% indicate more activity reductions, whereas values* >* 50% indicate more increases). Red and blue colors indicate more positive (activation) or more negative (deactivation) placebo effects, respectively. Regions are identified with the Mars brain atlas^70^ and the Yeo 7-network functional parcellation^71^, based on the MNI-coordinates of the peak.

Voxel-wise conjunction analysis (one-sided) with false discovery rate correction for multiple comparison.

*SalVentAttn* salience and ventral attention network, *Cont* frontoparietal executive control network.

Next, we directly compared activation during COND + INST-induced PA and INST-induced PA, corresponding to path a of the mediation analysis. COND + INST led to stronger activation in key modulatory areas associated with PA, such as DLPFC and dorsomedial prefrontal cortex (DMPFC), as well as in the temporo-occipital cortex, compared to INST (Fig. 3B; Table 2).

**Table 2:** Significant differences between placebo induction types INST and COND + INST

Cluster size (mm^3^)	X	Y	Z	%>0 INST	%>0 COND + INST	Diff.	qFDR	Region	Network
**COND + INST > INST**									
440	20	48	40	49.1	59.8	10.7	<0.0001	Right Rostral DLPFC	Default
	20	50	42	53.3	62.8	9.5	<0.0001	Right Rostral DLPFC	Default
	18	40	40	50.6	58.3	7.6	<0.0001	Right Rostral DLPFC	Default
	20	44	34	41.3	48.5	7.2	<0.0001	Right Rostral Superior DLPFC	Default
344	44	−70	44	50.9	60.3	9.4	<0.0001	Right Superior Visual Cortex	Vis
	44	−66	42	53.3	61.9	8.6	<0.0001	Right Superior Visual Cortex	Vis
	48	−66	44	54.0	62.2	8.2	<0.0001	Right Superior Visual Cortex	Vis
64	−4	32	42	52.1	61.1	9.0	<0.0001	Left Caudal DMPFC	Cont
72	60	−22	−14	51.8	60.6	8.8	<0.0001	Right Rostral MTG	Default
32	50	−62	48	51.8	60.5	8.7	<0.0001	Right Dorsal Inf. Parietal Cort.	Cont
72	56	−58	−16	51.8	60.4	8.6	<0.0001	Right Lateral Visual Cortex	DorsAttn
96	2	32	44	53.5	60.9	7.4	<0.0001	Right Caudal DMPFC	Cont
128	0	46	28	50.4	56.7	6.3	<0.0001	Left Rostral Medial PFC	Default
24	20	66	14	58.2	63.6	5.4	<0.0001	Right Rostral Superior DLPFC	Cont
**COND + INST < INST**									
32	−8	−44	−32	49.4	40.4	−9.3	<0.0001	Left Medial ITG	Cont
	−6	−44	−30	46.7	37.9	−8.8	<0.0001	Left Medial ITG	Cont
128	−22	−20	42	44.0	34.8	−9.3	0.00773	Left Dorsomedial Motor Cortex	Cont
	−20	−20	46	45.7	37.8	−8.0	<0.0001	Left Dorsomedial Motor Cortex	Cont
328	50	−6	28	50.1	41.0	−9.1	<0.0001	Right Dorsolateral M1	SomMot
	52	−10	32	49.9	41.4	−8.5	<0.0001	Right Dorsolateral S1	SomMot
	50	−12	28	49.9	41.5	−8.4	<0.0001	Right Dorsolateral S1	SomMot
32	−6	−44	−30	46.7	37.9	−8.8	<0.0001	Left Medial ITG	Cont
120	24	−48	10	45.5	36.7	−8.8	<0.0001	Right Rostral Medial Vis. Cort.	Cont
72	−28	−68	12	49.6	41.0	−8.7	<0.0001	Left Cuneus	Cont
32	34	−48	26	53.1	44.3	−8.7	<0.0001	Right Caudal MTG	Cont
24	−26	−50	24	45.5	37.0	−8.5	<0.0001	Left Medial Parietal Cortex	N/A
32	36	−8	18	48.9	40.8	−8.1	<0.0001	Right Middle Insula	SalVentAtt

Only cluster peaks surviving whole-brain voxel-wise FDR-correction, a cluster size ≥ 3 voxels (with *p* < .01) and a minimum distance of 20 mm to adjacent clusters are reported. X, Y and Z coordinates are reported in the MNI152 space, in units of mm. The percentage of participants with positive activation in the INST and COND + INST studies, and the difference, is shown in the rows “%>0 INST”, “%>0 COND + INST” and “Diff.”, respectively. The sign of the values is color coded; red: COND + INST > INST, blue COND + INST < INST. Regions are identified with the Mars brain atlas^70^ and the Yeo 7-network functional parcellation^71^, based on the MNI-coordinates of the peak.

Voxelwise regression (path a in mediation analysis) of placebo induction type and brain response (two-sided), with false discovery rate correction for multiple comparison.

*SomMot* somatomotor network, *Cont* frontoparietal executive control network, *Default* default mode network, *Vis* visual network, *DorsAttn* dorsal attention network.

In the case of DMPFC and the temporo-occipital cluster, increases were driven by activation during COND + INT, while DLPFC was rather driven by placebo-related deactivation within the INST condition (see horizontal bar plots on Fig. 3B,  C). The combination of verbal instructions and conditioning (COND + INST) was associated with significant signal decreases in sensory and motor areas, all of which were driven by de-activation in the COND + INST condition (Fig. 3B,  C). That is, only COND + INST produced significant decreases in S1 and mid-posterior insula. While the habenula has previously also been discussed to have an important role in pain modulation and placebo^15,28^, no differences in this region survived full-brain FDR correction for multiple comparisons. However, in a supplementary region-of-interest (ROI) analysis (Supplementary Table S8), we found that activity in the left habenula showed a significant placebo-related decrease with INST (harmonized effect size difference: −5.1%, *p* = 0.016) and COND + INST (−5.3%, *p* = 0.046), replicating our previous findings^15^.

While the primary focus of the present manuscript is the effect of induction type, we also conducted a supplementary analysis examining the effect of sex and age on placebo-related brain responses. We found that females, compared with males, showed stronger placebo-related deactivations in key pain-processing regions such as the anterior insula, ACC, and S1 (see Supplementary Fig. S7 and Supplementary Table S9 for details). These findings align with previous literature reporting sex-based differences in pain perception and modulation and underscore the importance of considering sex as a biological variable in pain and placebo research^29,30^. Furthermore, we identified five small clusters showing a significant age effect (all showing higher activation with increasing age, see Supplementary Fig. S8 and Supplementary Table S10). While a full mechanistic explanation of these results is beyond the scope of this meta-analysis, they highlight an important avenue for future investigation.

Furthermore, as the laterality of pain stimulation varied considerably across the involved studies (Supplementary Fig. S2), we conducted a supplementary analysis directly comparing placebo brain responses with left-sided versus right-sided stimulation, adjusted for age, sex and control pain ratings. Studies with bilateral stimulation (Bingel06) or without clear laterality (Elsenbruch, Theyson) were excluded, resulting in 13 studies with a total of 298 participants. This analysis revealed significant stimulation-side-dependent differences in activity mainly in the sensory and motor cortices, but also in parietal and frontal regions and the amygdala (Supplementary Fig. S9, Supplementary Table S11). These observed lateralization patterns were consistent with well-documented hemispheric specialization in sensory/nociceptive function.

**Fig. 4:**
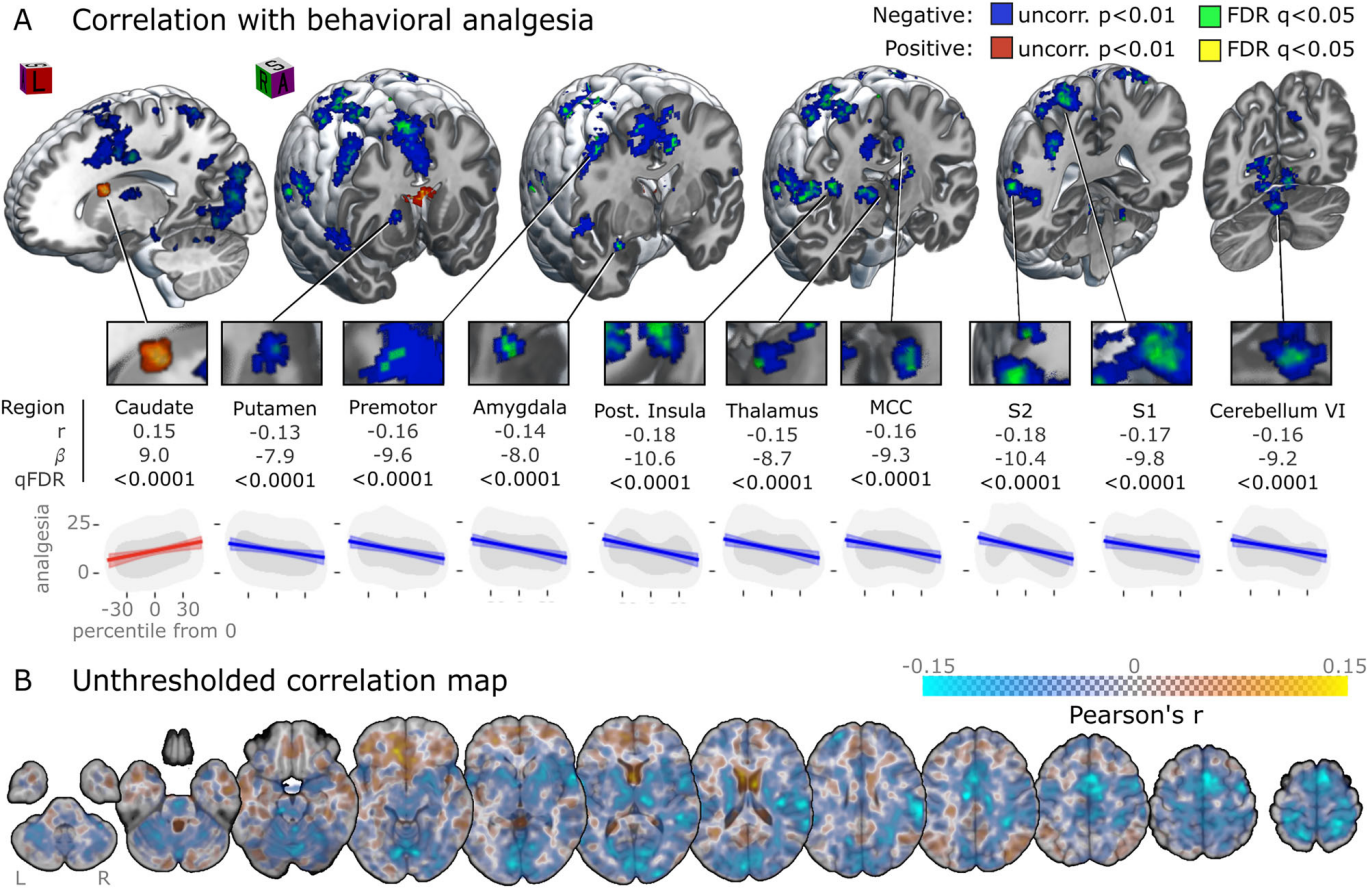
Path b: Correlation between placebo-related brain activity and behavioral placebo analgesia. **A** Areas where placebo-related activity reductions are significantly correlated with the degree of behavioral placebo analgesia (PA), shown in blue, include sensory-discriminative regions, mid-cingulate and premotor areas, cerebellum lobules VI and the amygdala. Only the caudate showed a significant placebo-related activity increase that positively scaled with behavioural analgesia. Correlations are depicted as regression lines (with 95% confidence intervals) overlayed on the data distribution (33 and 66% density iso-contours shown by the light and dark gray areas, respectively), for selected regions. Pearson’s correlation r, expected effect on ratings by a 10% change in population percentile *(β)* and FDR *q*-values are given for these regions (two-sided correlation test). See Table 3 for a comprehensive list. **B** Unthresholded correlation map. The transparency of the color map for plotting the effect size is modulated by effect size magnitude, as shown on the colorbar (smaller effects are more transparent). The spatial orientation of the three-dimensional visualizations is denoted by a cube on panel **A**, with letters denoting the superior (S), right (R) and anterior (A) views. In the case of uncorrected results (*p* < 0.01), we only show clusters that contain at least one voxel surviving FDR correction (*q* < 0.05).

**Fig. 5:**
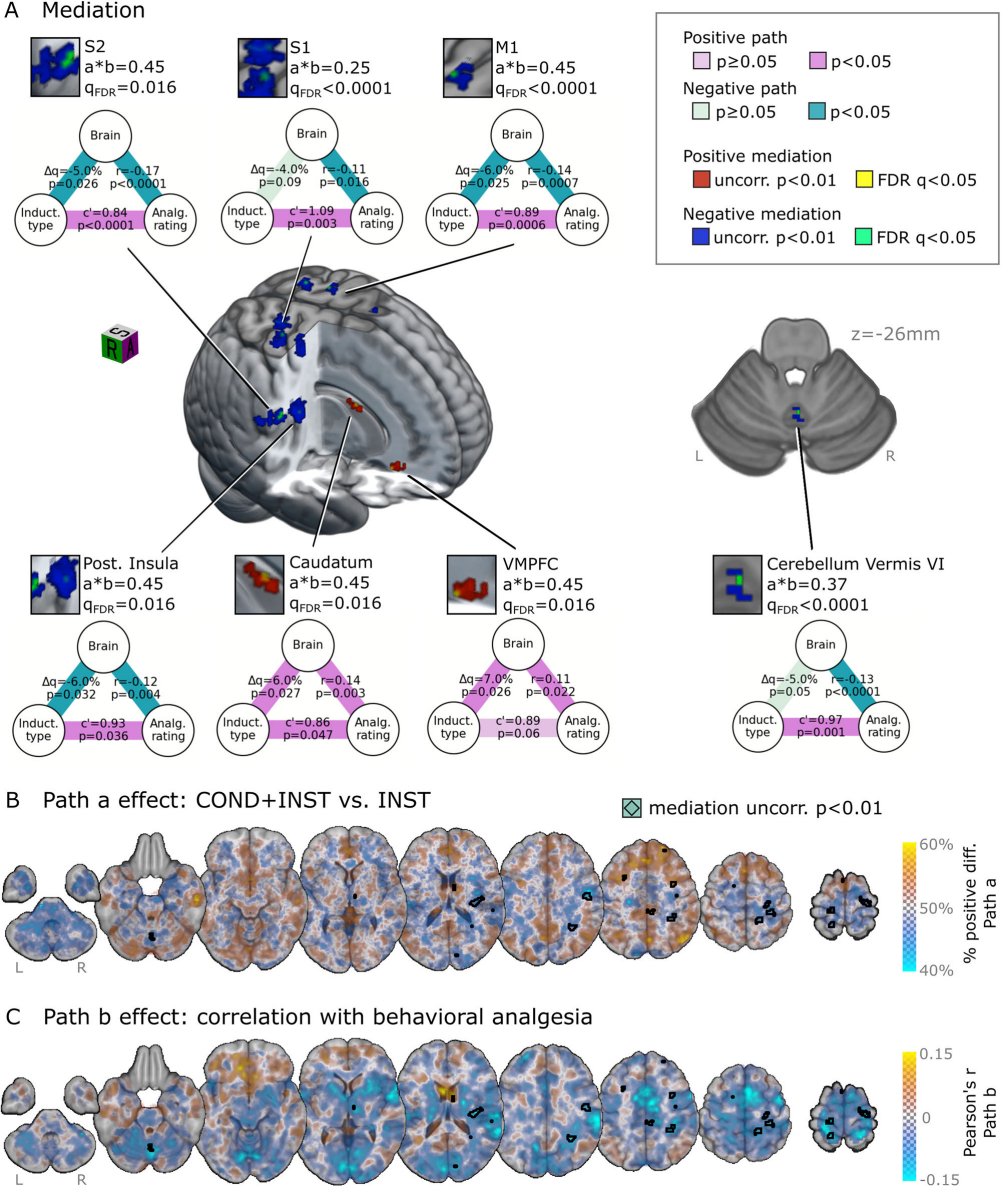
Brain mediators of placebo induction technique on behavioral placebo analgesia. **A** Areas where placebo induction type significantly mediates behavioral analgesia include the VMPFC (positive), caudate (positive), posterior insula (negative), S2 (negative) and S1 (negative). Red and yellow/blue and green shows regions where the effect of induction on behavioral analgesia is mediated by positive/negative, respectively, path a and path b effects. Mediation path diagrams are shown for selected regions. The path diagrams visualize the path a, path b and path c’ effects, and the corresponding uncorrected *p*-values. The mediation effect size (a x b) and the FDR *q*-value is given above the path diagrams. See Table 4 for a comprehensive list of regions with a significant mediation effect. Mediation analysis was implemented with linear models. The spatial orientation of the three-dimensional visualizations is denoted by a cube on panel **A**, with S, R and A denoting the superior (gray), right (right) and anterior (anterior) views. The z-coordinate of the cerebellar slice is given in mm. **B** Path a effect size (effect of induction type), with regions of significant mediation effect indicated with black contours. **C** Path b effect size (unthresholded brain-behavior correlation map), with black contours indicating significant positive mediation effect. On B and C, the transparency of the color map for plotting the effect size is modulated by the effect size magnitude, as shown on the color bars (smaller effects are more transparent). On all panels, in case of uncorrected results (*p* < 0.01), we only show clusters that contain at least one voxel surviving false discovery rate correction (*q* < 0.05).

Next, we evaluated the association between placebo brain responses and behavioral analgesia, controlling for induction type (path b of the mediation analysis; Fig. 4 and Table 3). We found widespread negative correlations in S1 and S2, posterior insular areas and MCC, premotor regions, amygdala, ventral thalamus and cerebellum (blue in Fig. 5). These results indicate larger PA with greater placebo-induced decreases in activity, and are consistent with findings in our previously published analysis^15^. In a supplementary analysis, we investigated whether a significant ‘induction type x analgesia’ interaction could be found in any voxels, but no voxels survived correction for multiple comparisons in this analysis. Nevertheless, we found a significant ‘induction type x analgesia’ interaction in the NPS (see below).

**Table 3:** Regions where placebo-related activity is significantly correlated with behavioral placebo analgesia

*Cluster size (mm*^*3*^*)*	*X*	*Y*	*Z*	*beta*	*r*	*qFDR*	*Region*	*Network*
**Positive Correlation**								
920	4	6	14	9.0	0.15	<0.0001	Right Caudate	Cont
**Negative Correlation**								
1088	38	−22	22	−10.6	−0.18	<0.0001	Right Ventral Inferior Parietal Cortex	SomMot
4160	62	−26	28	−10.4	−0.18	<0.0001	Right Ventral Inferior Parietal Cortex	SalVentAttn
3184	−14	−54	70	−9.7	−0.17	<0.0001	Left Medial Superior Parietal Cortex	DorsAttn
	−32	−36	68	−9.2	−0.16	0.00116	Left Dorsolateral S1	SomMot
	−12	−58	66	−9.0	−0.15	0.01304	Left Medial Superior Parietal Cortex	DorsAttn
	−36	−38	62	−6.5	−0.11	0.02825	Left Dorsolateral S1	SomMot
2368	−60	−28	42	−10.2	−0.17	<0.0001	Left Dorsal Inferior Parietal Cortex	DorsAttn
5056	44	−34	64	−9.8	−0.17	<0.0001	Right Dorsolateral S1	DorsAttn
	16	−54	64	−8.8	−0.15	<0.0001	Right Medial Superior Parietal Cort.	DorsAttn
	32	−36	70	−9.6	−0.16	<0.0001	Right Dorsolateral S1	SomMot
2344	56	18	−6	−10.2	−0.17	0.02979	Right Rostral STG	Cont
	52	18	−6	−8.1	−0.14	<0.0001	Right Rostral STG	SalVentAttn
	50	−4	4	−7.8	−0.13	<0.0001	Right Ventral Motor Cortex	SomMot
	58	−2	6	−8.0	−0.14	0.04023	Right Ventral Motor Cortex	SomMot
2024	64	−40	18	−9.2	−0.16	<0.0001	Right Caudal STG	DorsAttn
	48	−38	6	−8.5	−0.14	<0.0001	Right Caudal STG	Default
12,840	−4	−90	18	−9.4	−0.16	<0.0001	Left Cuneus	Vis
	2	−70	−26	−9.2	−0.16	<0.0001	Cerebellum VI anterior	N/A
	−4	−90	28	−7.3	−0.12	<0.0001	Left Cuneus	Vis
	−4	−82	8	−7.8	−0.13	<0.0001	Left Cuneus	Vis
13,296	−12	2	42	−9.3	−0.16	<0.0001	Left Dorsomedial Premotor Cortex	SomMot
	0	8	62	−9.6	−0.16	<0.0001	Left Dorsomedial Premotor Cortex	SalVentAttn
	6	−32	46	−7.7	−0.13	<0.0001	Right Posterior Cingulate Cortex	SomMot
	8	−4	50	−8.2	−0.14	<0.0001	Right Dorsomedial Motor Cortex	SomMot
800	−18	−14	16	−8.0	−0.14	<0.0001	Left Thalamus	N/A
928	16	−18	12	−8.7	−0.15	<0.0001	Right Thalamus	N/A
456	30	−96	8	−8.9	−0.15	<0.0001	Right Caudal Medial Visual Cortex	Vis
536	−14	−64	32	−8.8	−0.15	0.00906	Left Medial Parietal Cortex	Cont
368	32	−6	−14	−8.0	−0.14	<0.0001	Right Putamen	N/A
248	32	−46	22	−8.1	−0.14	<0.0001	Right Caudal MTG	N/A
1936	38	−28	44	−8.5	−0.14	<0.0001	Right Dorsolateral S1	DorsAttn
	32	−46	34	−8.3	−0.14	0.00953	Right Superior Parietal Cortex	DorsAttn
	18	−52	64	−8.4	−0.14	<0.0001	Right Dorsomedial S1	DorsAttn
1688	52	6	48	−8.4	−0.14	<0.0001	Right Dorsolateral Motor Cortex	SalVentAttn
384	−58	4	2	−8.0	−0.14	0.00213	Left Ventral Motor Cortex	SalVentAttn
344	18	10	8	−7.9	−0.13	<0.0001	Right Putamen	N/A
448	−26	54	28	−7.9	−0.13	<0.0001	Left Rostral Dorsal Prefrontal Cortex	Default

Only cluster peaks surviving FDR-correction, a cluster size ≥ 20 voxels (with *p* < 0.01) and a minimum distance of 20 mm to adjacent clusters are reported. X, Y and Z coordinates of the peak activation are reported in MNI152 space, in units of mm. Effect size (beta) can be interpreted as the predicted VAS difference between the participants with the least and most activated region, within a study. Effect size is converted to Pearson’s correlation coefficient (*r*; red: positive, blue: negative). Regions are identified with the Mars brain atlas^70^ and the Yeo 7-network functional parcellation^71^, based on the MNI-coordinates of the peak.

Voxel-wise correlation analysis (two-sided) with false discovery rate correction for multiple comparison.

*DorsAtt*n dorsal attention network, *Default* default mode network, *SomMot* somatomotor network, *Cont* frontoparietal executive control network, *Vis* visual network, *SalVentAttn* salience and ventral attention network.

### Brain mediators of the effect of induction type on behavioral placebo analgesia

When evaluating the mediation effect i.e. the joint effects of induction type on brain placebo response (path a) and brain placebo response on behavioral PA (path b), we found FDR-significant mediation (a*b) in several locations (Fig. 5, Table 4). We distinguished two types of mediation effects: We refer to ‘positive mediation’ where more behavioral analgesia in COND + INST is mediated by more brain activity (i.e., both path a and b are positive). By ‘negative mediation’ we refer to cases, when more analgesia in COND + INST is mediated by less brain activity (i.e. both path a and b are negative). Placebo brain responses in the VMPFC and right caudate were found to be positive mediators of the effect of induction type on behavioral analgesia. These regions exhibit a combination of greater activation with COND + INST and a positive relationship between placebo-induced activity and analgesia. The most pronounced negative mediators were regions in the posterior insula, S1 and S2 indicating placebo analgesia being mediated by stronger deactivation of pain-related activity. These regions exhibit a combination of reduced activation with COND + INST and greater analgesia with more placebo-induced deactivation.

**Table 4:** Regions where placebo induction type significantly mediates behavioral analgesia

*Cluster size (mm*^*3*^*)*	*X*	*Y*	*Z*	*a beta*	*b corr*	*a×b*	*qFDR*	*Region*	*Network*
Positive									
40	8	2	16	6.1	0.14	0.5	< 0.0001	Right Caudate	N/A
104	10	26	–16	7.5	0.11	0.4	0.01642	Right VMPFC	Cont
Negative									
880	54	–18	24	–5.5	–0.17	0.5	< 0.0001	Right Dorsal Inferior Parietal Cort.	SalVentAttn
176	–16	–46	68	–6.5	–0.16	0.6	0.00759	Left Dorsomedial S1	SomMot
112	22	–38	64	–3.9	–0.16	0.4	0.01068	Right Dorsomedial S1	SomMot
552	34	–18	18	–7.3	–0.16	0.6	0.02415	Right Ventral Inferior Parietal Cort.	SomMot
160	30	–38	44	–6.7	–0.14	0.5	0.01642	Right Superior Parietal Cortex	DorsAttn
152	–16	–30	70	–5.1	–0.14	0.4	0.01642	Left Dorsomedial S1	SomMot
496	–62	–26	22	–6.3	–0.14	0.5	0.02374	Left Ventral Inferior Parietal Cortex	SalVentAttn
200	26	–20	68	–6.0	–0.14	0.4	0.03642	Right Dorsomedial Motor Cortex	Cont
48	2	–60	–26	–5.3	–0.13	0.4	< 0.0001	Cerebellum VI anterior	N/A
64	–14	0	62	–5.1	–0.13	0.3	< 0.0001	Left Dorsomedial Premotor Cortex	Cont
568	–26	–70	12	–6.8	–0.13	0.5	< 0.0001	Left Cuneus	Cont
272	12	–78	20	–5.7	–0.13	0.4	0.01637	Right Rostral Medial Visual Cortex	VisCent
120	40	–14	18	–6.1	–0.12	0.4	0.00125	Right Ventral Inferior Parietal Cort.	SomMot
16	–16	–2	42	–7.2	–0.12	0.5	0.02142	Left Dorsomedial Premotor Cortex	Cont
40	–20	–78	10	–5.7	–0.12	0.4	0.04147	Left Cuneus	VisCent
824	34	–50	28	–5.1	–0.11	0.3	< 0.0001	Right Dorsal Inferior Parietal Cortex	Cont
16	28	–32	56	–5.5	–0.11	0.3	< 0.0001	Right Dorsolateral S1	SomMot
104	28	–32	60	–4.3	–0.11	0.2	< 0.0001	Right Dorsolateral S1	SomMot
40	38	–10	42	–6.3	–0.10	0.3	< 0.0001	Right Dorsolateral Motor Cortex	SomMot
96	–52	–42	6	–7.3	–0.10	0.4	< 0.0001	Left Caudal STG	Default
176	30	–30	52	–5.4	–0.10	0.3	0.00559	Right Dorsolateral S1	SomMot

Only cluster peaks surviving FDR-correction and with a minimum distance of 20 mm to adjacent clusters are reported. X, Y and Z coordinates of the peak activation are reported in the MNI152 space, in units of mm. Path a effect size (a) can be interpreted as the predicted VAS difference between the participants with the least and most activated region. Path b (b) is given as Pearson’s correlation coefficient. Effect sizes are color coded; red: positive mediator, blue: negative mediator. Regions are identified with the Mars brain atlas^70^ and the Yeo 7-network functional parcellation^71^, based on the MNI-coordinates of the peak. *DorsAttn* dorsal attention network, *Default* default mode network, *SomMot* somatomotor network, *Cont* frontoparietal executive control network, *Vis* visual network, *SalVentAttn* salience and ventral attention network.

To clarify any remaining risks to power and inference, we quantified voxel-wise minimal detectable effects (MDEs) for each of the three inference paths by deriving detectability thresholds from the corresponding sign-flip null distributions. The resulting MDE maps, shown in Supplementary Fig. S12, indicate high sensitivity across most brain voxels, with reduced detectability largely confined to peripheral cerebellar and orbitofrontal regions, consistent with susceptibility-related fMRI signal dropout.

### Effects of conditioning on multivariate pain signatures

**Fig. 6:**
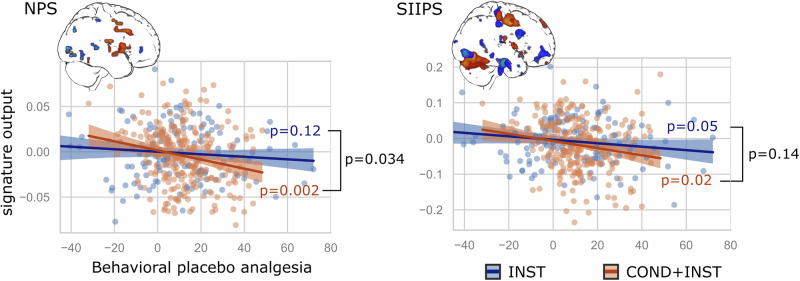
NPS, but not SIIPS, predicts behavioral analgesia induced by COND + INST than by INST alone. The scatter plots show that the relationship between analgesia and NPS response was significantly stronger for COND + INST (orange; r = −0.19, β = −0.0005, 95% CI = [−0.0008, −0.0001], *p* = 0.002) than for INST-alone (blue; r = −0.10, β = −0.0001, 95% CI = [−0.0004, −0.0001], *p* = 0.121). The difference in these associations was confirmed by a significant interaction effect (black; T(409) = −1.83, β = −0.0002, 95% CI = [−0.0004, −0.00001], *p* = 0.034). No such interaction was observed for the SIIPS (COND + INST: r = −0.15, β = −0.0005, 95% CI = [−0.001, 0.00001], *p* = 0.0502; INST: r = −0.16, β = −0.0008, 95% CI = [−0.001, −0.00005], *p* = 0.02, interaction: T(390) = −1.17, β = −0.0002, 95% CI = [−0.0007, 0.0002] *p* = 0.14) Regression lines and 95% confidence intervals are plotted separately for the INST and COND + INST induction techniques. Blue and orange *p*-values correspond to the INST and COND + INST induction techniques, corrected for age and sex. Black *p*-values correspond to the interaction effect between induction technique and behavioral analgesia; with the significant *p*-value for NPS indicating that the association between the NPS and behavioral analgesia is significantly stronger in case of COND + INST. Glass brains represent the predictive weights of the signatures (red: positive weight, blue: negative weight). Regression model was used (two-sided) for estimation.

In the final analysis, we investigated the extent to which the placebo analgesic effect, induced by either COND-INST or INST, correlated with changes in the engagement of two established pain signatures in the brain: the Neurologic Pain Signature (NPS, Fig. 6 left) and the Stimulus Intensity Independent Pain Signature (SIIPS, Fig. 6 right). Pooled over induction types, both the NPS and SIIPS exhibited a statistically significant negative correlation with behavioral analgesia (NPS: *T*(390) = −3.169, *r* = −0.159, *p* = .002, β = −0.0003, 95% CI = [−0.0005, −0.0001], two-tailed; SIIPS: (*T*(390) = −3.109, *r* = −.155, *p* = 0.003, β = −0.0007, 95% CI = [−0.0001, −0.0002], two-tailed), with no significant difference between them. When contrasting induction types, we observed a stronger NPS-analgesia association with COND + INST (*T*(252) = −3.025, *r* = 0.19, *p* = 0.002, β = −0.0005, 95% CI = [−0.0008, −0.0001], two-tailed) than with INST only, (*T*(136) = −1.184, *r* = 0.1, *p* = .121, β = −0.0001, 95% CI = [−0.0004, −0.0001], two-tailed). The difference between induction types was statistically significant (interaction: *T*(409) = −1.83, *p* = 0.034, β = −0.0002, 95% CI = [−0.0004, −0.00001], two-tailed). Detailed results are presented in Supplementary Table S12. The SIIPS showed no statistically significant interaction with induction type. For detailed results, see Supplementary Table S13.

## Discussion

In this systematic, individual-level meta-analysis, we examined placebo analgesia (PA) and its neural correlates when induced in healthy volunteers through either a combination of conditioning and verbal instructions (COND + INST) or verbal instructions alone (INST).

First, we confirmed that applying conditioning together with verbal instructions results in stronger PA. Second, we identified regions involved in the placebo condition regardless of induction technique, and regions where the placebo response significantly differs between induction techniques. Third, we corroborated and complemented previous results about the association between placebo response in the brain and behavioral PA, both on a voxel-wise basis and as reflected by two multivariate brain signatures. Finally, we identified brain mediators of the effect of different induction techniques on PA.

Adding conditioning to verbal instructions increased the PA effect by 33% and doubled the standardized effect size (*g* = 0.74 vs. 0.37). This is consistent with a previous meta-analysis showing a stronger behavioral effect of conditioned PA (^3^; comparison of 14 studies with sample sizes between 24 and 136 each; Cohen’s *d* = 0.85 for INST (14 studies) and *d* = 1.45 COND + INST (three studies)) and subsequent studies comparing the effect of verbal instruction with the combined use of conditioning and verbal instruction. It is important to place the magnitude of our behavioral findings in the appropriate context. The observed VAS increase in analgesia with conditioning, while statistically robust, is below thresholds typically considered a minimal clinically important difference (MCID) in pain research involving patients (e.g. refs. ^31,32^). This should be interpreted in light of our study’s mechanistic goal and inclusive analytical approach. We analyzed data from all participants, rather than pre-selecting for ‘placebo responders’, which necessarily lowers the average group effect size. Our findings therefore reflect the average benefit of adding conditioning across a heterogeneous population of healthy volunteers in an experimental setting, rather than the potential effect size in a clinical population or a responder subgroup.

At the neural level, placebo analgesia induced by both INST and COND + INST was characterized by deactivation of regions involved in nociceptive processing, such as the insula, putamen and posterior cingulate cortex. Both induction techniques also led to activation in the dorsal inferior parietal cortex (IPC) and in the inferior rostral part of the DLPFC, an area that is a key component of the descending pain modulatory network^33^ and has been implicated in orchestrating top-down pain modulation in several studies investigating PA^8,34,35^.

By contrast, the combination of conditioning and instructions also recruited the DMPFC as a second top-down modulatory region. Further clusters were identified in the angular gyrus in the parietal cortex and superior parts of the DLPFC, which seemed to be caused by the absence of deactivation, rather than active engagement (Fig. 3B). The recruitment of these additional descending pain modulatory regions with COND + INST was accompanied with stronger decrease in key sensory brain regions with this induction technique.

While both induction types clearly involve learning, (see ref. ^36^), one obvious difference is the felt experience of pain reduction that is the hallmark of the conditioning procedure in a placebo manipulation. In the context of placebo analgesia, this typically involves a covert reduction in stimulation intensity, which is intended to simulate the pain relief expected from an analgesic treatment. In contrast, verbal instructions only suggest that the perceived pain intensity will be lower, without providing this first-hand experience of reduced pain. It could therefore be argued that conditioning provides a more accurate idea of pain reduction than can be conveyed by verbal instructions alone. The effect of this difference can be understood through conceptualizing perception as inference process, which have become a prominent perspective on perception more generally^37,38^. In this framework, prior information provided by instruction or conditioning acts as a reference point (or prior, in Bayesian terminology) against which sensory input is evaluated. Priors play a pivotal role in shaping the perceptual process of an individual. Stronger, more precise priors exert greater influence on the underlying inferential process, shaping the perceived pain experience to align more closely with these expectations, even when the sensory evidence remains unchanged. This suggests that the effectiveness of a strategy lies in its ability to generate a robust prior, which in turn, leads to a more pronounced influence on subsequent perception. According to the Bayesian causal multi-source integration model, this depends not only on the quantity of prior information but also on the degree of agreement among information from various sources^39^. When multiple sources provide consistent and complementary information (such as verbal instructions and experienced pain relief in the context of combined instructions and conditioning) and are inferred to share a common cause, these inputs are integrated during the inferential process and effectively increase the confidence in the prior. This, in turn, is likely to reduce variability across participants when instructions and conditioning are combined. On the contrary, the priors induced by instructions only—without providing any hands-on experience—are likely more driven by individual differences in personal history and varying subjective assumptions, explaining the higher variance we observed in behavioral placebo effects with INST, as compared COND + INST. Future studies should also examine whether the magnitude and variability of pain relief experienced during the conditioning phase relates to the degree of subsequent neural deactivation, which would provide additional support for the Bayesian predictive coding framework proposed here^40^.

Interestingly, our findings of stronger engagement of prefrontal regions by instructions paired with conditioning than instruction alone also align with observations from studies using dynamic reversal learning paradigms. In these paradigms, one strategy (e.g., conditioning) is used to induce learning and subsequent testing assesses whether another strategy (e.g., instructions) can reverse the learned effect. Studies employing this approach have demonstrated that the presence of instructions increases activation in the DLPFC^18,21^, similarly to our study. Further, on the behavioral and peripheral-physiological level, instructions can modulate conditioned fear and autonomic responses (see refs. ^18,19,41,42^), as well as brain responses associated with learning, including the VMPFC and striatum^18,19,21^, two regions that were also found to mediate the effects of induction method on placebo analgesia in our study. Interestingly, prior work suggests that the amygdala learns similarly regardless of instructions^18,43^. Testing whether this finding extends to placebo analgesia would require studies that investigate effects of conditioning in the absence of instructions, which has not been tested with neuroimaging other than in the context of pain-predictive cues^19^. This approach offers a promising way to explore the joint, dynamic impact of instructions and learning and may help to find the most robust and persistent way to induce placebo analgesia.

It could be argued that only some of the regions engaged during COND + INST are in fact causally related to the stronger analgesic effect. To test this assumption, we examined the correlation between brain responses and reported placebo analgesia across individuals. As expected, placebo responses in the brain were negatively associated with placebo analgesia across a widespread network of pain processing regions, including the thalamus, operculum, insula, middle cingulum and sensory regions. In contrast, no positive correlations survived correction for multiple comparisons across the whole brain.

Although there was no significant voxel-level interaction between brain-behavior correlation and induction type, we observed that the NPS, a weighted multivariate signature^13^ linked to nociception, predicted behavioral analgesia in the COND + INST condition more accurately than in the INST only condition. This suggests that the COND + INST condition induces stronger top-down modulation of nociceptive processing, potentially explaining its enhanced analgesic effect. This is also in line with the outcome of our whole-brain analysis showing stronger deactivation in nociceptive regions including the posterior insula and S1 in the condition combining instructions with conditioning.

Finally, we identified brain regions where the effect of induction type on behavioral placebo analgesia was mediated by brain placebo effects. The strongest ‘positive mediators’ were the VMPFC and the caudate, suggesting that these regions play a crucial role in translating the induction type into observable behavior. The VMPFC has long been associated with value-based processing^44,45^, cognitive control^46^, emotion regulation^47^, and belief updating^48^. Importantly, alterations in VMPFC activity and connectivity have been reported for different types of chronic pain^49^, particularly during the transition from acute to chronic pain states. This connection is particularly relevant to our findings, as the belief-updating mechanisms we observe in placebo analgesia may represent similar neural processes that, when dysregulated, contribute to the learned behavioral components underlying chronic pain symptomatology.

The differential engagement of the VMPFC and caudate in placebo responses likely reflects the integration of richer, self-referential memories into expectations when conditioning is involved. The VMPFC shows increased activation during the retrieval of autobiographical memories, anchoring personal experiences within a narrative framework^50^. Lesions in this region impair imagining detailed future scenarios, underscoring its role in weaving memories into prospective thinking^48,51^, and lead to stronger reliance on instructions in the context of pain-predictive cues^48^. This suggests, that in the context of placebo expectations, conditioning does more than reinforce expectancy—it likely integrates learned associations into a broader self-referential memory framework. Conditioning paired with instructions encourages participants to incorporate episodic experiences of the process (e.g., relief or positive outcomes) into their placebo expectations. This creates richer, more personally meaningful priors, in the Bayesian sense, that more strongly guide perception and behavior compared to abstract instructions alone.

Furthermore, the VMPFC is known to contain expected value signals that inform our choices^52^. While INST, in itself, can establish a general prior belief about the expected effects of the (sham) treatment, only having hands-on experiences makes it possible to estimate the subjective “value” (efficacy) of the treatment. If, during the test phase, general expectations about the upcoming event (induced by verbal instruction) and concrete memories regarding the subjective value of the applied treatment (induced by conditioning) are inferred to be causally related, the two types of priors reinforce each other^39^. This causal inference process will result in a stronger top-down modulatory force, consistent with the observed decreased activity in the ‘negative mediators’: the posterior insula, S2, and S1.

The caudate, part of the dorsal striatum, may complement this by integrating reward-related learning and supporting goal-directed behavior (e.g., ref. ^53^). Conditioning may strengthen the role of the caudate in encoding the association between placebo cues and anticipated relief. Unlike the habit-focused putamen, the caudate facilitates goal-directed processes that align with purposeful expectancy of benefit. Together, the VMPFC and caudate, connected via corticostriatal circuits, may facilitate the integration of self-referential memory (VMPFC) and reinforcement history (caudate) into placebo expectations.

Our findings raise the question of whether the larger effect in the combined condition is simply due a stronger modulation of predictive processes (i.e., regardless of the technique or whether different types of learning are combined), due to the combination of different learning strategies (e.g., conditioning and verbal instruction), or due to the unique contribution of conditioning. In an early study, Colloca and colleagues demonstrated that increasing the number of conditioning sessions could enhance the placebo effect, implying that reinforcing learning through additional repetitions may strengthen the overall outcome^24^. Schafer et al. (2015)^25^ also showed that after several days of conditioning, placebo effects became larger and more resistant to changes in expectations; placebo effects persisted even when participants knew they had been taking a placebo and no longer expected relief. To investigate the influence of different strategies,^54^ recently compared the effect of different combinations of conditioning, instruction, and observational learning on PA induction. Their results showed that the combination of all three strategies was superior to the use of any single strategy, but, surprisingly, it did not exceed the effectiveness of the combination of only two strategies—and interestingly, the combination of conditioning and instructions did not outperform conditioning alone. It is important to highlight that our study did not specifically delineate the neural mechanisms of conditioning alone. While conditioning alone has been a prevalent method for modulating pain perception and pain-related fear in a plethora of studies^55,56^, these investigations did not combine the conditioning procedure with treatment expectation. The results of our comparison between instructions and instructions combined with conditioning can therefore not necessarily be attributed to conditioning; instead, they are likely to be the result of an interaction between the two methods of inducing PA. Indeed, there are studies showing that conditioning alone, without suggestion or treatment expectations, shows limited efficacy in inducing PA^57^.

Another limitation of our study is that the findings are based on a cohort of relatively young, healthy adults. As both brain function^58^ and placebo effects^59^ can change across the lifespan, the generalizability of these specific neural mediators requires further investigation. Indeed, our own results showed a significant effect of age on behavioral analgesia (*β* = −0.2898, *T* = −1.981, *p* = 0.048), as well as significant positive associations with placebo brain responses and age (Supplementary Fig. S8, Supplementary Table S10). These findings underscore the need for future research systematically examining placebo mechanisms across different age groups, including patient populations affected by neurodegenerative diseases^60^.

A further limitation is that the laterality of pain stimulation varied considerably across the included studies (Supplementary Fig. S2). In our supplementary analysis examining the effects of stimulation side on placebo brain responses, we did not find significant differences in regions that exhibited significant mediation effects in our main analysis (including the VMPFC, caudate, S2, insula, or cerebellar vermis), suggesting that the findings of our mediation analysis are unlikely to be strongly influenced by laterality.

Heterogeneity across the studies included in this meta-analysis extends beyond stimulation laterality, encompassing differences in duration, intensity, and conditioning protocols and includes diverse pain stimuli. While identifying consistent neural effects across these varied methodologies strengthens the generalizability of our core findings, this heterogeneity is also a source of variance. We have accounted for this in our statistical models by including study-level covariates, which absorb the variance attributable to these protocol differences along with other site-specific effects. Furthermore, we assessed the covariance between induction type and these methodological variables and found no evidence for significant associations (see Supplementary Fig. S10 and Supplementary Table S14 for details). However, we cannot fully exclude the possibility that residual confounding may have contributed to our results, warranting cautious interpretation. Fully disentangling the specific influence of such methodological parameters would require a different, more targeted meta-analytic approach, and importantly, more neuroimaging studies that systematically vary these parameters.

Like any meta-analysis of published literature, our results could be influenced by publication bias, wherein studies with significant findings are more likely to be published. While no evidence of publication bias was detected in our dataset (see Supplementary Fig. S11, we cannot entirely rule out its presence. However, our primary aim is not to estimate the absolute effect size of placebo analgesia across studies, but to elucidate the neural mechanisms underlying the different induction techniques, given that the placebo effect has been successfully induced. Studies without a behavioral effect, while important for estimating overall prevalence, would be uninformative for our specific research question.

Finally, our use of mediation analysis entails important interpretive caveats. While this analysis provides a powerful framework for identifying potential neural pathways linking induction type to analgesia, it does not establish causality for paths linking observed variables (as opposed to experimentally randomized variables, which afford stronger causal interpretation^61^). A key assumption for making causal claims is the absence of unmeasured confounding between the mediator (here, brain activity) and the outcome (here, behavioral analgesia). In a complex psycho-biological context such as this, it is impossible to rule out the influence of all potential unmeasured confounders, such as stable personality traits or prior beliefs not captured in our model. Therefore, we do not make directional causal claims about the observed associations between regional brain activity and analgesia. However, because placebo vs. control conditions were randomly assigned, it is reasonable to infer that placebo effects on brain activity are causal. Our mediation results, which depend jointly on both effects, should thus be interpreted as identifying statistically significant pathways consistent with a proposed mechanistic model, highlighting promising targets for future studies explicitly designed to test for causal directionality.

In conclusion, our meta-analysis provides robust evidence for distinct neural correlates of PA induced by instructions alone and by a combination of instructions and conditioning. Our findings suggest that conditioning engages neural mechanisms beyond those of verbally induced PA. This improved understanding of the efficacy and underlying mechanisms of different placebo induction techniques not only informs ways to maximize and exploit placebo effects in experimental studies. These findings may provide preliminary insights relevant to future research on targeted interventions in clinical settings aimed at optimizing analgesic strategies, including in populations that may show altered placebo-related modulation, such as patients with neurodegenerative diseases^62,63^.

## Methods

### Data acquisition

The present study is a systematic meta-analysis of individual participant data across *k* = 16 peer-reviewed, within-participant placebo neuroimaging studies (see Supplementary Table S2 for an overview of all included studies). Data from the included studies, together with data from four more between-participant studies, served as a basis for previous meta-analyses, examining placebo effects on the NPS^14^ and across the entire brain^15^. In contrast to these previous analyses, the scope of the present analysis was restricted to within-participant studies, allowing us to consider the individual magnitude of PA when investigating the neural correlates of PA induced by verbal instructions alone (INST) or by verbal instructions together with conditioning (COND-INST). Our analysis included single-participant data from a total of *n* = 415 participants (*n* = 409 after excluding participants with missing pain ratings). The present research complies with all relevant ethical regulations, including written consent, as all individual studies obtained ethical approval from their respective institutions.

A detailed description of the data collection procedures can be found in Zunhammer et al. (2018)^14^. In brief, we first performed a systematic literature search to identify experimental functional magnetic resonance imaging (fMRI) studies of PA. Eligible studies met the following criteria: (a) published in English in a peer-reviewed journal; (b) constituted an empirical investigation; (c) involved human participants; (d) employed functional neuroimaging of the brain during evoked pain; and (e) induced pain under matched placebo and control conditions. For the present study, we added that (f) studies had to be conducted within-participant, with each participant serving as their own control and (g) availability of pain ratings in both the placebo and the control conditions. Authors of all identified studies were contacted and invited to share their data. We collected single-participant, first-level, whole-brain standard space summary images of pain response (statistical parametric maps) from the original analyses, corresponding pain ratings separately for placebo and control conditions, experimental design parameters, and demographic data. From studies that applied conditioning, we only used data from the test phase (and not the conditioning phase). Our original data acquisition procedure^14,15^ identified 96 published articles, of which 28 were selected as eligible according to above criteria a-e. Authors of 20 studies provided data (including 71% of eligible studies and 79.4% of eligible participants). In the present analysis, we excluded studies with a between-participant design (according to criteria f and g), leading to a final dataset consisting of *k* = 16 studies and *n* = 415 individuals (*n* = 409 after excluding participants with missing pain ratings). Details on data acquisition are provided in Supplementary Methods 1-2, Supplementary Table S3 and Supplementary Fig. S5, as well as in refs. ^14,15^. The included studies induced PA either by verbal instructions alone (INST; *k* = 5, *n* = 147) or by a combination of verbal instructions and conditioning (COND-INST; *k* = 11, *n* = 268, see Supplementary Table S2). Seven studies used a topical cream as a placebo, five used an intravenous infusion, and two used sham acupuncture. One study each used either sham transcutaneous nerve stimulation (TENS) or a placebo nasal spray. The majority of studies employed thermal stimulation (*k* = 11), followed by laser or distension (each *k* = 2) and electrical pain (*k* = 1; see Supplementary Figs. S1-S3 for details). 35.4% of the participants were female (as assessed via self-report; see Supplementary Fig. 4 for the sex distribution across studies). Details on the conditioning procedures used in the different studies can be found in Supplementary Table S1.

### Risk of bias assessment

We used the Cochrane risk of bias tool^27^ to evaluate the risk of bias for studies included in the present meta-analysis (details are in Supplementary Methods 3 and Supplementary Table S4). We assessed biases from selection (which arises via insufficient randomization), performance (via insufficient blinding of participants or treatment providers), detection (via insufficient blinding of analysts), attrition (by missing data), reporting (via underreporting of nonsignificant studies), and sequence (which is potentially introduced by within-participant designs).

### Harmonization

#### Behavioral data

To construct harmonized measures of the behavioral placebo response across studies, we first converted pain intensity ratings to a score between 0–100 and then subtracted the placebo condition ratings from the control condition ratings (positive values correspond to analgesia). Additionally, we obtained study-wise standardized effect size summaries of the behavioral PA by calculating Hedges’ g scores, as is common in meta-analyses^64^. Similarly to the Cohen *d*, Hedges *g* is based on the mean difference between conditions divided by standard deviation, but with an additional correction for small sample bias. Specifically, as only within-participant studies were included, we used Hedges *g*_rm_, which is based on the SD of within-participant differences corrected for within-participant correlations (for details see refs. ^14,15^).

### Neuroimaging data

To address center effects and differences in image acquisition procedures across studies, we harmonized the brain data (first-level control-placebo contrast images) using quantile transformation, applied independently to each voxel within each study. Quantile transformation, similar to ranking, is a widely used data harmonization technique^64^ that assigns each data point its quantile within the study, resulting in a value between 0 and 1. This process forces the transformed data to follow a uniform distribution, thereby eliminating differences in data distributions across studies. However, the conventional form of quantile transformation also removes all information about study-specific means, making it unsuitable for comparing effects across studies (e.g., assessing the overall effects of induction techniques on PA, as in path A of a mediation analysis). This limitation can be overcome by using a zero-preserving quantile transformation, which involves converting the original values to their quantile distance from zero. Specifically, we calculated:

1 $${q}_{0}({x}_{i,j})=\,\frac{{{rank}}_{i}({x}_{i,j})\,-\,0.5}{{n}_{x}}\,-\,\frac{{\sum }_{k=1}^{{n}_{x}}I({x}_{i,k} < 0)}{{n}_{x}}$$

where ${q}_{0}$ is the zero-preserving quantile transformation, ${x}_{i,j}$ is the value in voxel a given voxel in participant j from study i, ${{rank}}_{i}(.)$ denotes the rank transformation across all participants in study i, using average ranking for ties, and $I({x}_{i,k} < 0)$ is an indicator function that equals 1 if ${x}_{i,k}$ is positive and 0 otherwise. This equation describes how each voxel value in the individual level placebo brain images is transformed by ranking it within its study, normalizing the rank (to get quantiles), and adjusting for the proportion of negative values in the batch.

### Data analysis

#### Standardized behavioral summary data

The effect of induction technique on the standardized effect size summaries of behavioral PA across studies was investigated by a *T*-test comparing the Hedges’ *g*-values between the two groups of studies.

### Mediation analysis

To investigate the neural correlates of the effect of induction type on behavioral PA, we performed a voxel-wise mediation analysis, with the main aim of evaluating the indirect effects of induction type (X) on behavioral placebo analgesia (Y) through placebo-related neural activity as a mediator variable (*M*). Throughout our mediation analysis, we corrected for age, sex and control pain rating, and modelled studies with dummy regressors orthogonalized to induction type. As COND + INST vs. INST compares different groups of studies that could vary in methodological variables like stimulus type, we assessed the covariance between induction type and various methodological variables like the type, location and duration of pain stimulation or the repetition and echo times of the respective fMRI sequences. We found no significant association with any of these variables (see Supplementary Fig. S10 and Supplementary Table S14 for details). The mediation analysis was implemented using ordinary least squares linear models with a custom analysis code written in Python (see section on Code Availability).

First, the mediator M (brain data) is modeled as a function of the independent variable X (induction type) and covariates using linear regression. This step estimates the effect of induction type on brain activity (path a). Second, the outcome Y (behavioral PA) is modeled as a function of the mediator M (brain activity) and covariates using linear regression. This step estimates the correlation of brain activity changes with behavioral placebo analgesia across participants (path b). Finally, the indirect effect (also known as the average causal mediation effect or ACME) is calculated as the product of the coefficients from path a and path b.

Furthermore, we estimated the group-mean brain activity, assuming dummy coding for the two groups of studies. The reference group mean is derived from the intercept, and the other group’s mean is adjusted by the group difference.

The significance of the path a and path b effects, the indirect (mediation) effect and the group-mean activations were assessed by repeating the whole analysis on 10 000 surrogate datasets, each constructed by flipping the sign of the brain data for randomly selected participants and rerunning the whole procedure, including harmonization and mediation analysis. Note that the approach is equivalent to permutation testing on with considering the two brain maps (placebo and control pain) of a participant as an exchangeability group.

The actual (unflipped) effect estimates were contrasted to the resulting null-distribution to obtain uncorrected *p*-values which were adjusted for multiple comparisons across the brain by false discovery rate (FDR) correction. As the precision of the estimation of very low *p*-values with permutation testing is limited by the number of permutations, we used tail approximation to calculate more accurate *p*-values^65^. Specifically, the sign flipping-based null-distribution was subject of tail approximation (in each voxel independently), by fitting a generalized Pareto distribution to the tail of the distribution^66^. The optimal tail ratio was selected from a pre-defined set of tail rations (from 0.1 to 0.3, with increments of 0.05), based in the goodness of fit, as measured by the Kolmogorov-Smirnov statistic^67^. *P*-values were then approximated based on the fitted Pareto distribution:

2 $$p=(1-F({observed}{{\_}}{value}\,-\,{tail}\,{{\_}}{threshold}))\times {tail}{{\_}}{ratio}$$

Where *F*(x) is the Cumulative Distribution Function of the generalized Pareto distribution.

### CA1: Variance analysis

We investigated whether the variance of the behavioral placebo response (unharmonized but converted to VAS 0-100) differs between INST and COND + INST in a two-step approach. First, we fitted a mixed effect model that explains behavioral analgesia with age, sex and control pain rating (all centered) as fixed effects and study as random effect. We then performed Levene’s test of equal variances on the residuals of this model, to compare the variance of the residuals in the INST vs. COND + INST studies. The mixed-effects model was implemented using the *statsmodels* package (v0.14.2) in Python with Restricted Maximum Likelihood (REML) estimation, and Levene’s test was conducted using the *scipy.stats* package (v1.13.1).

### CA2: Conjunction analysis

We performed a conjunction analysis on the two group-mean maps (mean placebo brain response to INST and COND + INST) by taking the maximum *p*-value from the two images in each voxel, and correcting for FDR across brain voxels, as described by Nichols an colleagues^68^. Specifically, for each voxel we combined the two component tests (INST_mean and CONDINST_mean) by taking the minimum of their -log_10_(*p*) values, which is equivalent to testing the conjunction via the maximum component *p*-value (*p*_conj_ = max(*p*_*1*_*, p*_*2*_)). The resulting conjunction map was evaluated both at uncorrected thresholds (*p* < 0.01 and *p* < 0.05) and after Benjamini-Hochberg false-discovery-rate correction (*q* < 0.05); effect direction was assigned from the sign of the corresponding combined beta map.

### CA3: Pain signatures

To test whether placebo analgesia induced by the two techniques scaled with changes in the overall activity in pain-related brain regions, we correlated the placebo analgesic effect with activation changes in two established neural signatures of pain, namely the Neurologic Pain Signature (NPS;^13^) and the Stimulus Intensity Independent Pain Signature (SIIPS;^26^), separately for COND-INST and INST. Engagement of the NPS was been shown to robustly correlate with pain induced by different levels of noxious input^13,14,69^, whereas activity in the SIIPS reflects stimulus-independent variations in reported pain intensity^26^. When comparing the outputs of the investigated neural signatures (NPS and SIIPS), we first computed the output of the signatures for each participant as the dot product of the signatures’ predictive coefficient map and the participants’ harmonized brain imaging data. The resulting signature scores were then fed into a linear model, signature response ~ induction type * behavioral PA + *CTR pain rating + age + sex + study*. As in the mediation analysis, studies were modelled with dummy regressors orthogonalized to induction type. For all coefficients of interest, we performed bootstrapping (with 10,000 bootstrap samples) to obtain 95% confidence intervals and *p*-values.

### Visualization

MRIcroGL (v28.5.2017) and the python package *nilearn* was used to create illustrations of statistical parametric maps. Activation peaks were summarized using the Mars brain atlas and the Yeo 7-network functional parcellation, based on the MNI-coordinates of the peak. All result maps follow the neurological convention, where the left side corresponds to left hemisphere in coronal and axial sections.

On Figs. 3–5, when visualizing uncorrected results (*p* < 0.01), we only show clusters that had at least one voxel that survived false discovery rate correction (*q* < 0.05).

Activation peaks were summarized using the Mars brain atlas^70^ and the Yeo 7-network functional parcellation^71^, based on the MNI-coordinates of the peak.

### Reporting summary

Further information on research design is available in the Nature Portfolio Reporting Summary linked to this article.

## Supplementary information

Supplementary Information

Reporting Summary

Transparent Peer Review file

## Data Availability

The derivative/summary data generated in this study are publicly available on GitHub (https://github.com/pni-lab/placebo-conditioning-meta-analysis; 10.5281/zenodo.18837986). Individual-level raw data included in this meta-analysis are available only under restricted access due to legal and ethical constraints linked to the original studies (including participant-consent and ethics-approval limits). Access may be requested by contacting ulrike.bingel@uk-essen.de and providing a detailed description of the planned scientific research, including any reproducibility analyses. A response to data requests will be provided within 4 weeks. Upon approval of the proposed research plan, data may be shared under a data-use agreement.

## Code availability

Analysis code for carrying out data cleaning is available at: https://github.com/mzunhammer/PlaceboImagingMetaAnalysis. The complete code for reproducing the analyses and figures presented in the manuscript are available at: https://github.com/pni-lab/placebo-conditioning-meta-analysis10.5281/zenodo.18837986).
