# Supplementary material for: Meta-analytic evidence for distinct neural correlates of conditioned versus verbally induced placebo analgesia
Source: Nat Commun. 2026 Jul 17;17:6538. doi: 10.1038/s41467-026-74743-0 (PMC13379574; doi:10.1038/s41467-026-74743-0)
Supplement: Supplementary file 1 — Supplementary Information [file 41467_2026_74743_MOESM1_ESM.pdf]

Supplementary Information

for the manuscript entitled

Meta-analytic evidence for distinct neural correlates of conditioned vs verbally induced placebo analgesia

by

Tamas Spisak, Helena Hartmann, Matthias Zunhammer, Balint Kincses,
Katja Wiech, Tor D. Wager, & Ulrike Bingel on behalf of the Placebo Imaging Consortium

Supplementary Figures

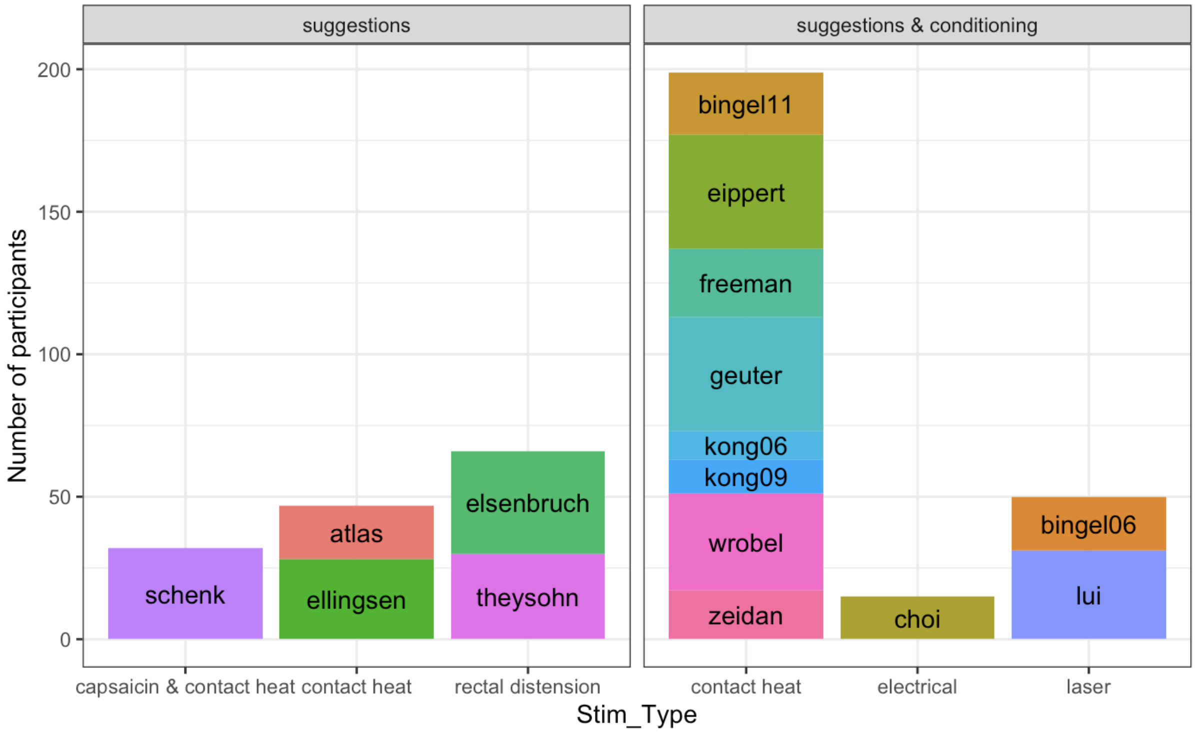

Supplementary Figure S1. Number of participants and studies grouped by the type of pain stimulation. *The data is based on k = 16 studies and n = 409 participants. Stacked bar plots depict the number of participants for each study, separated by the type of placebo induction and the type of pain stimulation.*

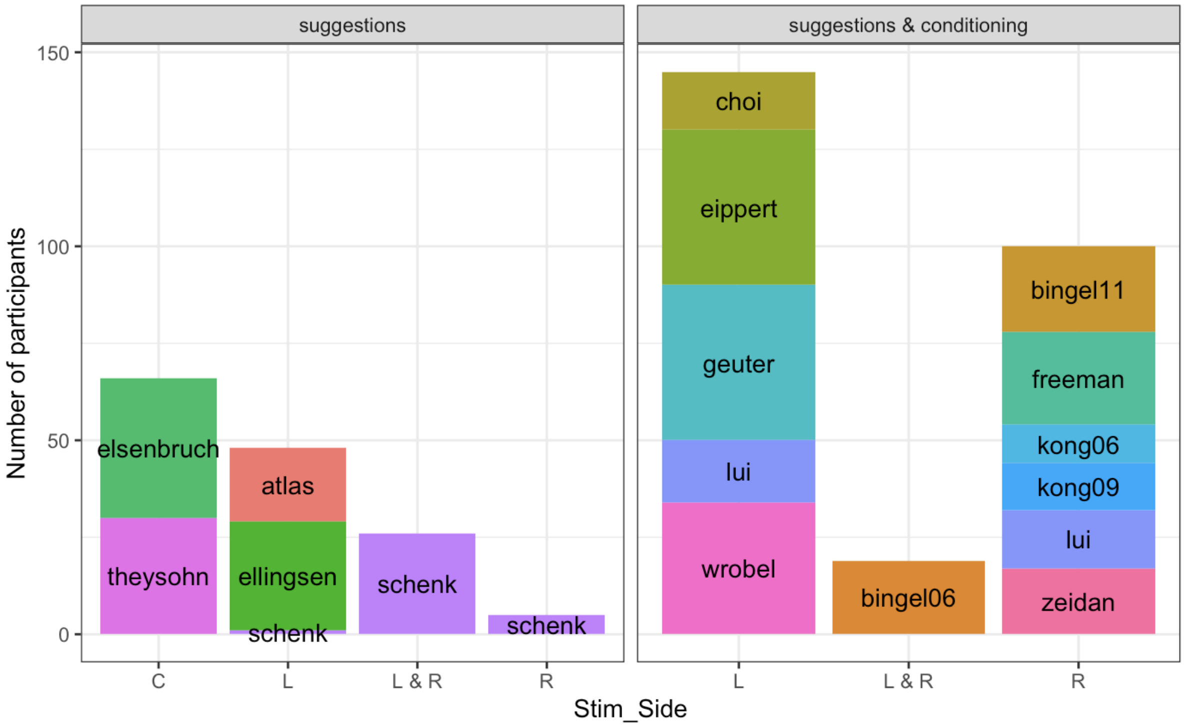

Supplementary Figure S2. Number of participants and studies grouped by the laterality of pain stimulation. *L = left, R = right, C = no laterality (rectal distension studies). The data is based on k = 16 studies and n = 409*

participants. Stacked bar plots depict the number of participants for each study, separated by the type of placebo induction and the laterality of pain stimulation.

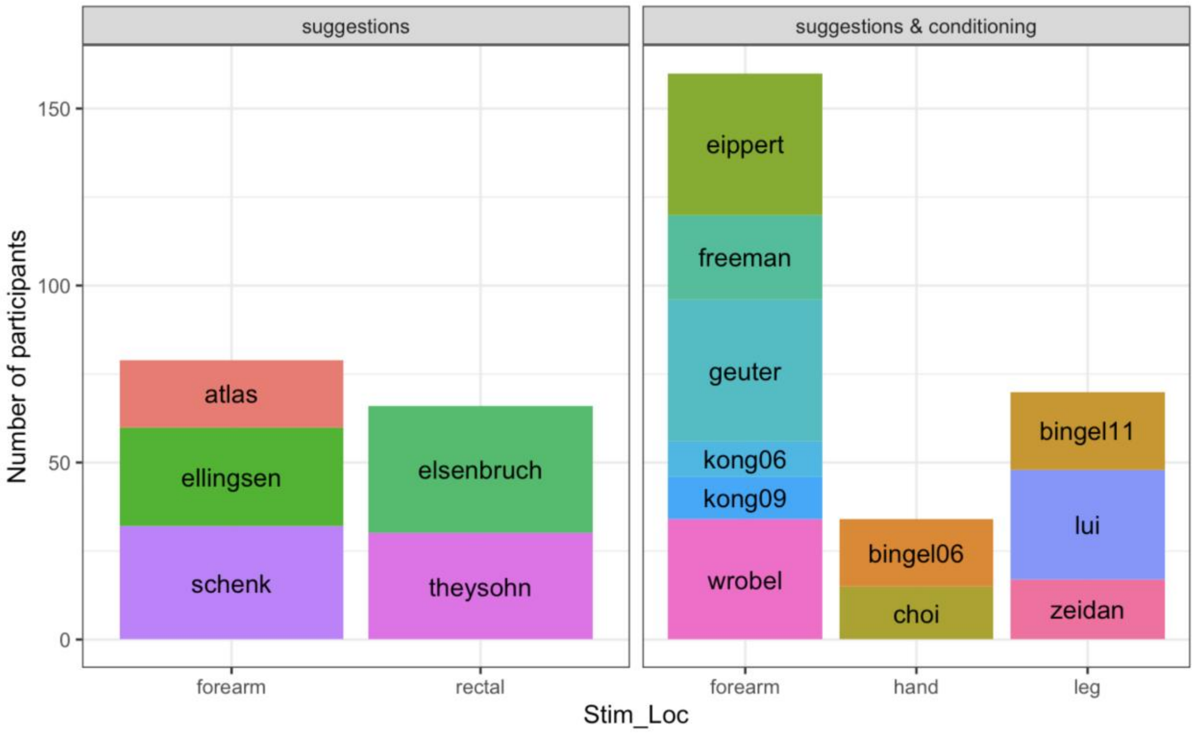

Supplementary Figure S3. Number of participants and studies grouped by the location of pain stimulation. *The data is based on k = 16 studies and n = 409 participants. Stacked bar plots depict the number of participants for each study, separated by the type of placebo induction and the location of pain stimulation.*

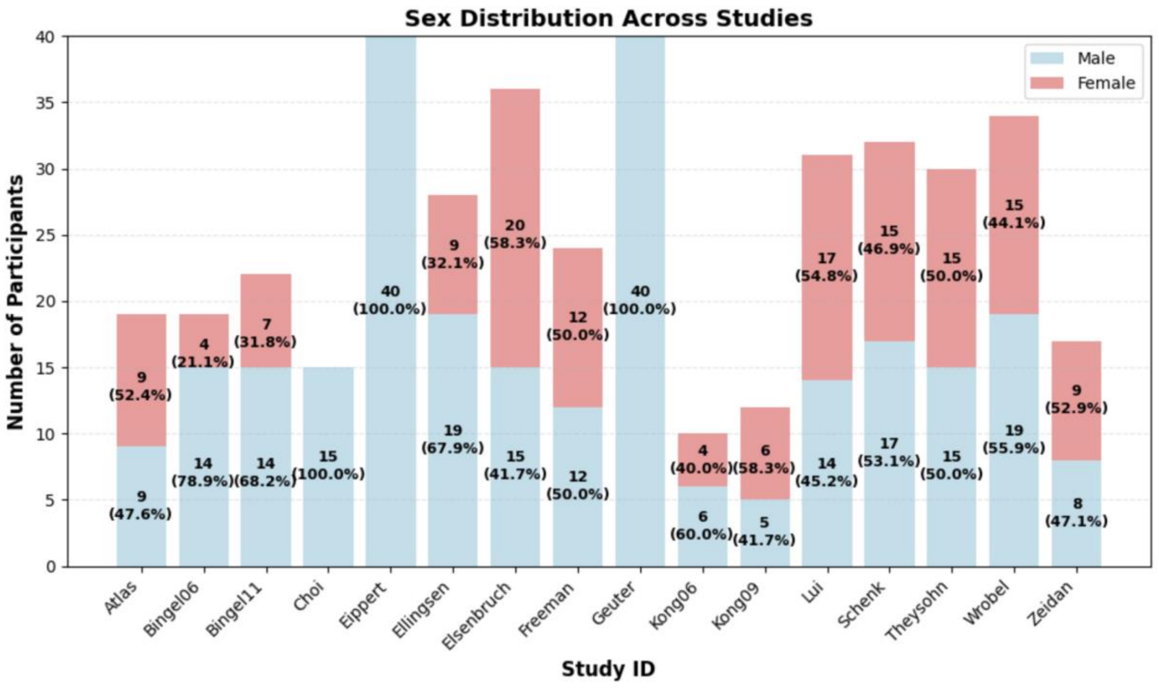

Supplementary Figure S4. Sex distribution across the studies. *The data is based on k = 16 studies and n = 409 participants. Stacked bar plots depict the number of participants for each study, separated by participant sex.*

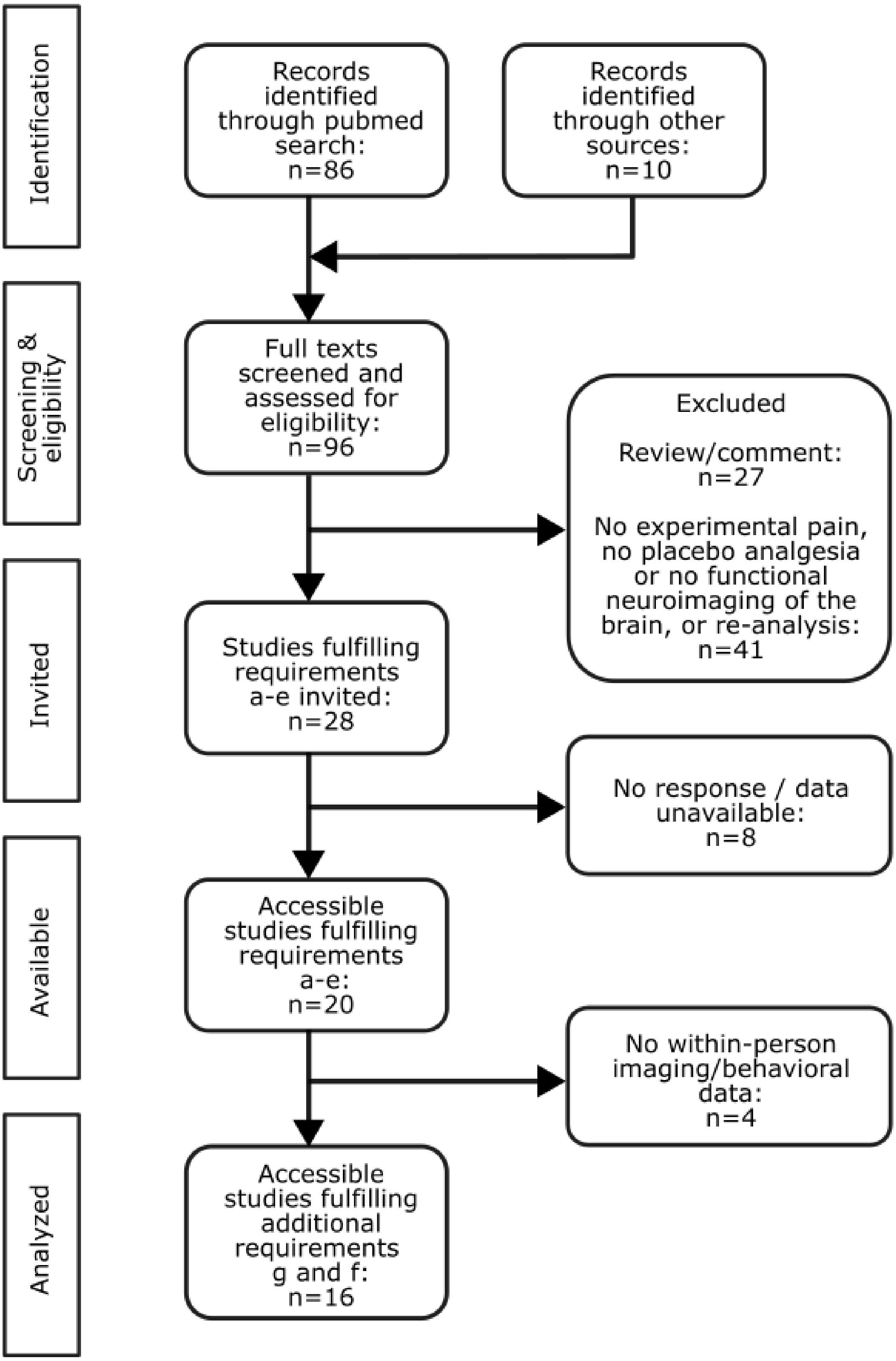

Supplementary Figure S5. PRISMA Flowchart of data acquisition. *The flowchart depicts the data collection process. It involved record identification, eligibility screening, invitation to share data, and summarized available and analyzed studies.*

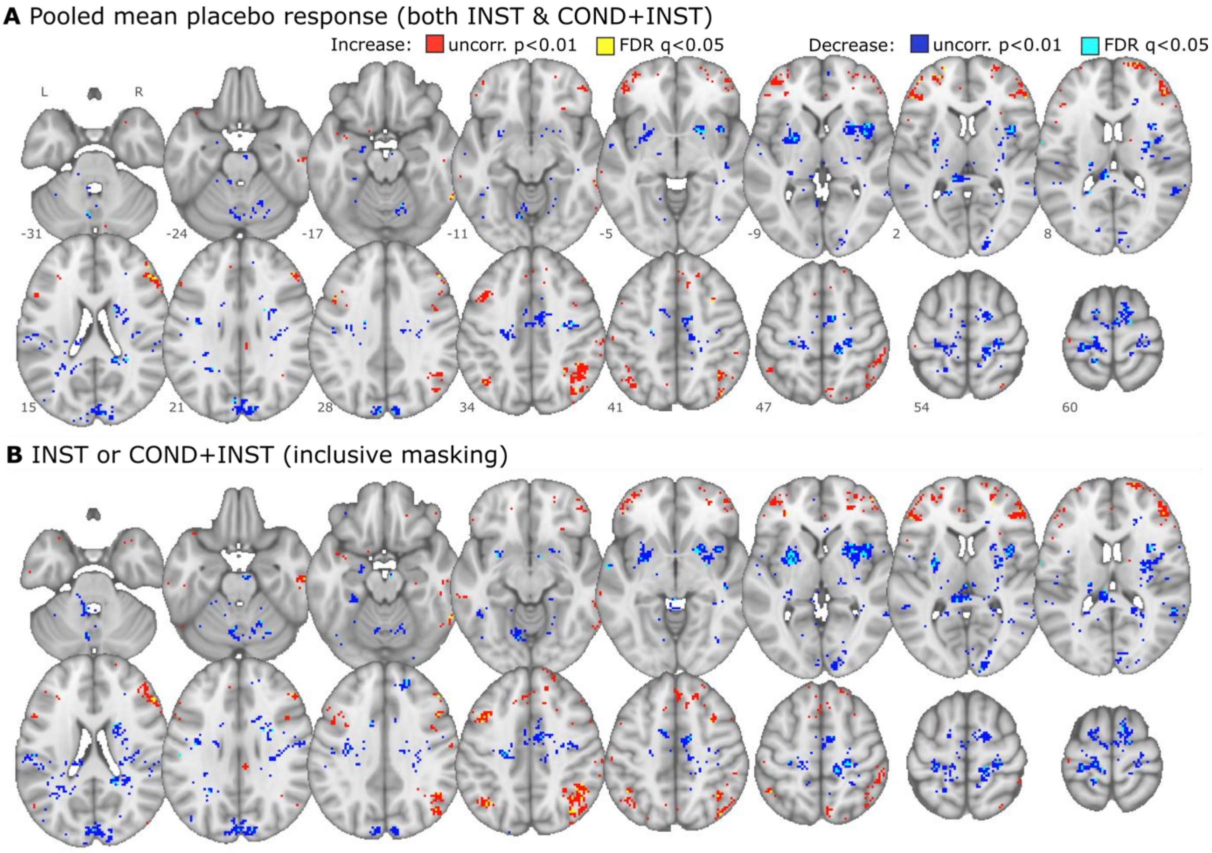

Supplementary Figure S6. Group-level brain activation for placebo analgesia. *A: Pooled across all participants (regardless of induction type). B: The union of INST and COND+INST (inclusive masking). All results are adjusted for age, sex and control pain ratings. Images are shown in neurological convention (left is left). Standard-space Z-coordinates are shown for each slice on the first panel. The second panel visualizes the same slices. See* ***Supplementary Table S6*** *for a comprehensive list of activations significant after FDR correction.*

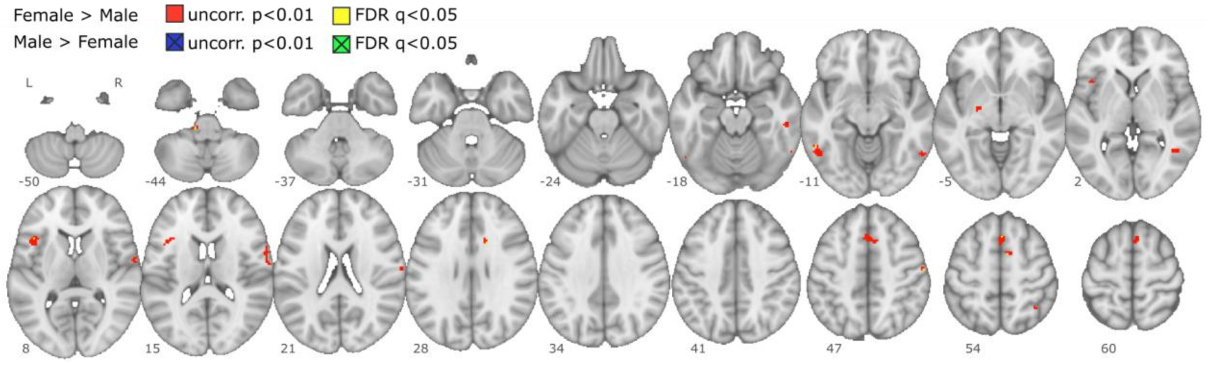

Supplementary Figure S7. Sex distribution across studies. *Sex differences in placebo analgesia. We found multiple clusters where placebo-related brain activity was significantly higher in females (FDR q < 0.05), including regions like the anterior insula, DMPFC, ACC, S1 and basal ganglia. In the case of uncorrected results (p < .01, red), we only show clusters that contain at least one voxel surviving false discovery rate correction (q < 0.05). No voxels showed increased activity in males after FDR-correction. See* ***Supplementary Table S7*** *for a comprehensive list of activations significant after FDR correction.*

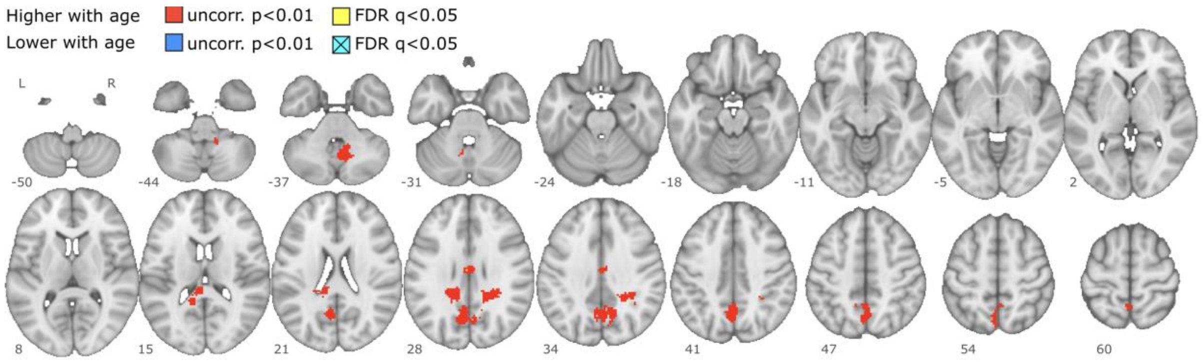

Supplementary Figure S8. Association between placebo analgesia and age, adjusted for induction type, sex and control pain ratings*. We found multiple clusters where placebo-related brain activity showed a significant positive association with age in 6 different peak locations (FDR q < 0.05) in the right cerebellum, left precuneus, left and right posterior cingulate cortices and left middle cingulate cortex. In the case of uncorrected results (p < .01, red), we only show clusters that contain at least one voxel surviving false discovery rate correction (q < 0.05). No voxels showed negative association with age after FDR-correction. See* ***Supplementary Table S6*** *for a comprehensive list of activations significant after FDR correction.*

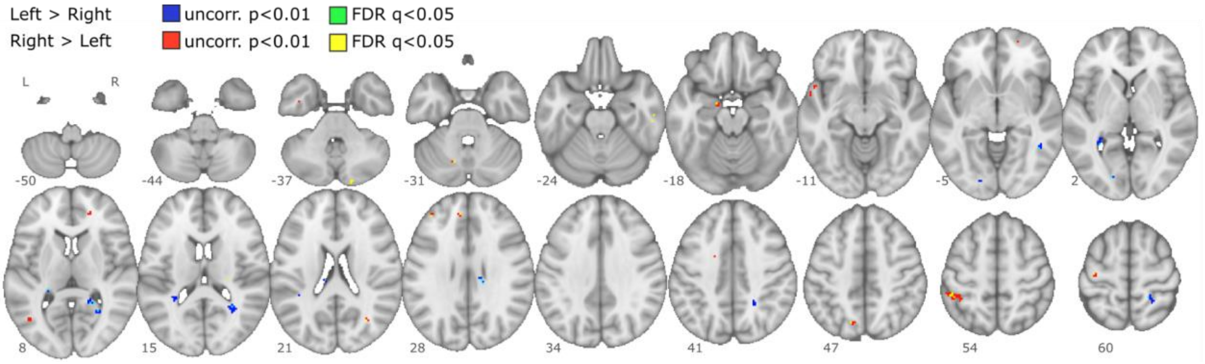

Supplementary Figure S9. Differences in placebo analgesia in relation to stimulation laterality, adjusted for age, sex and control pain ratings. *In this analysis, we excluded studies with bilateral stimulation (Bingel06) or without clear laterality (Elsenbruch, Theyson), resulting in 13 studies with a total of 298 participants. We found multiple clusters where placebo-related brain activity was significantly related to stimulation side (FDR q < 0.05), including mostly sensory and motor areas, but also parietal and frontal regions and the amygdala. The lateralization of differences generally reflected the well documented hemispheric specialization in sensory/nociceptive function. In the case of uncorrected results (p < .01, red and blue), we only show clusters that contain at least one voxel surviving false discovery rate correction (q < 0.05). Images are shown in neurological convention (left is left). Standard-space Z-coordinates are shown for each slice. See* ***Supplementary Table S5*** *for a comprehensive list of activations significant after FDR correction.*

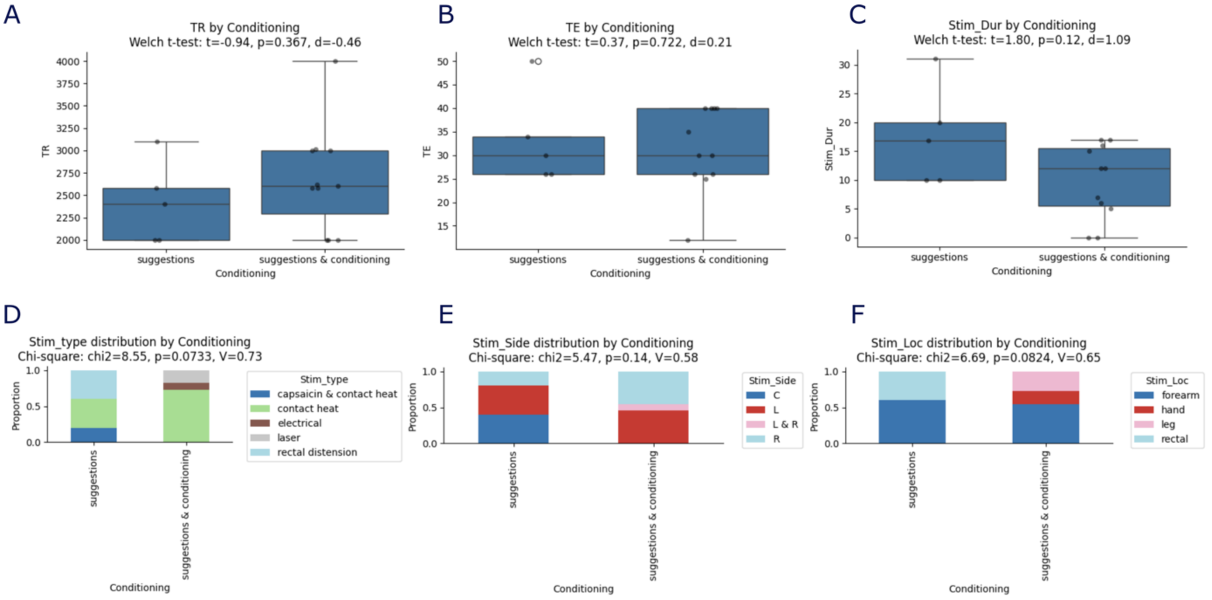

Supplementary Figure S10. The association of induction type with potential confounders. *A: repetition time, B: echo time, C: stimulus duration, D: stimulus type, E: stimulation side, F: stimulus location. For continuous variables (A-C), we used a Welch T-test. For categorical variables (D-F), we used a* χ^2*-test. See also* ***Supplementary Table S13***.

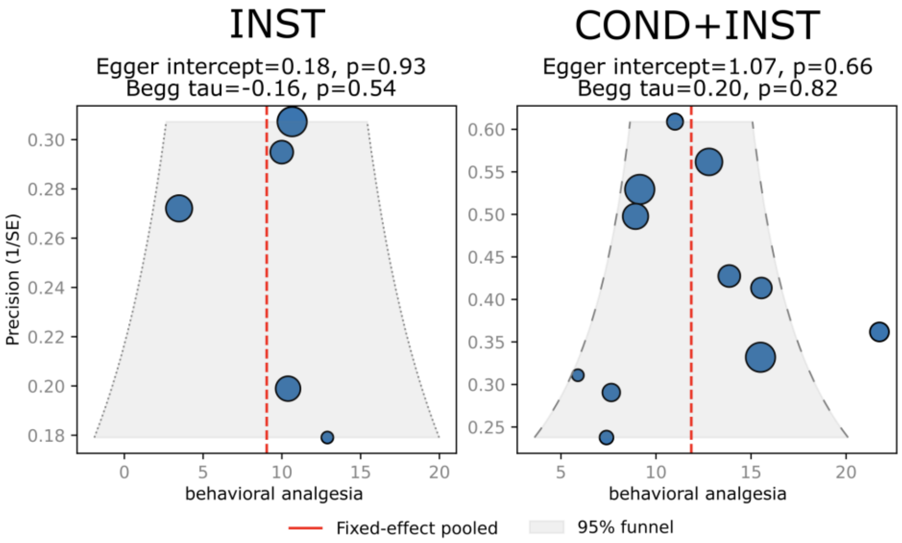

Supplementary Figure S11. Funnel plots of behavioral analgesia by induction type (INST; COND+INST). *Each point is a study with marker size representing study sample size. The x-axis shows behavioral analgesia (higher values = stronger behavioral placebo analgesia), the y-axis shows precision (inverse standard error). The red dashed line marks the fixed-effect pooled estimate; the shaded region is the 95% funnel. Egger's regression and Begg's rank-correlation tests indicate no evidence of funnel asymmetry in either group of studies (all p's > .05; see exact values in panel headers).*

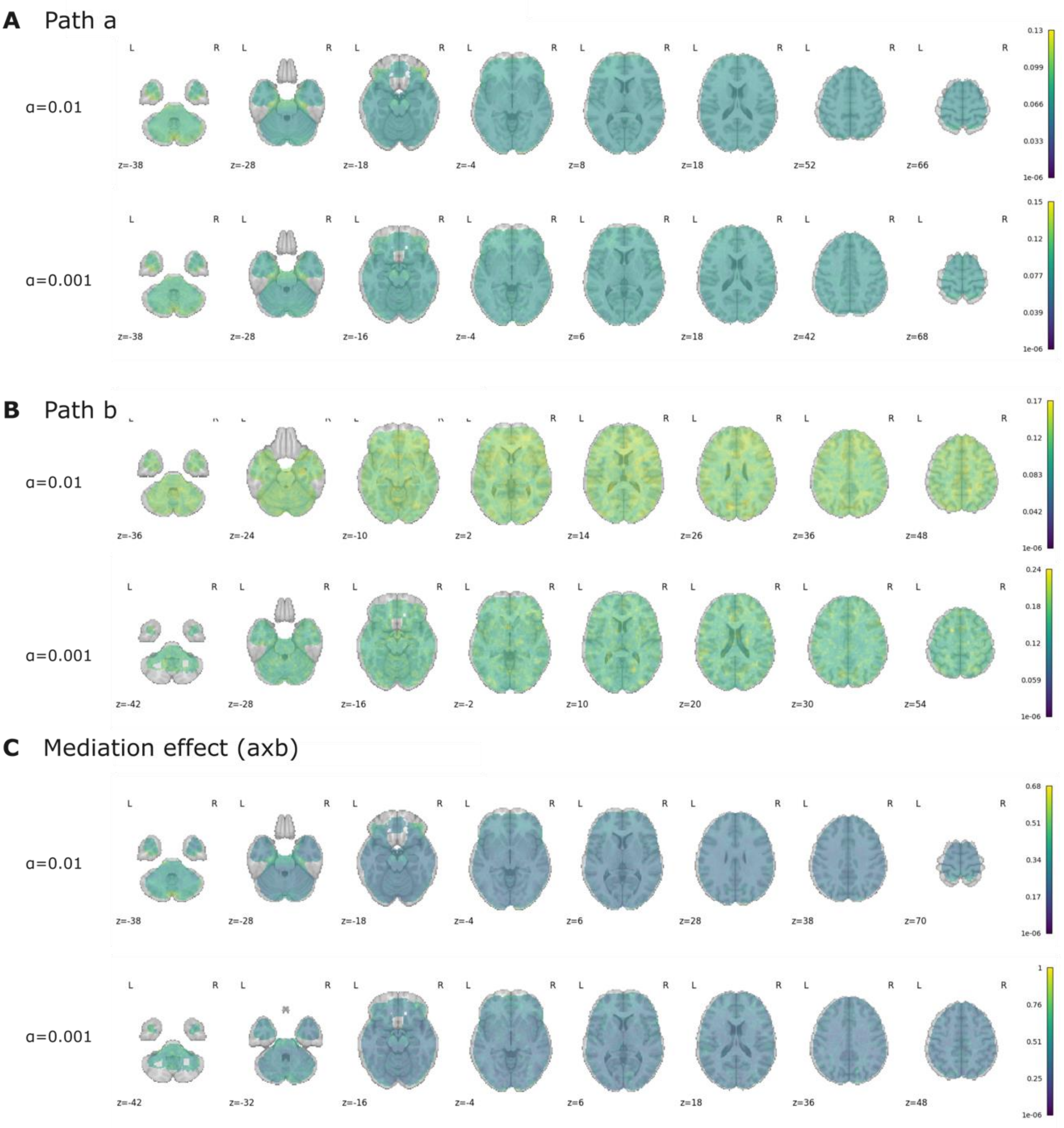

Supplementary Figure S12. Voxel-wise minimal detectable effects (MDEs) for the mediation analysis paths. *(A) Path a (X→M): induction type effect (COND+INST vs. INST) on quantile-harmonized placebo brain responses; MDE is shown in the harmonized quantile units (approximately a percentage-point shift in the proportion of participants with positive placebo responses; (B) Path b (M→Y): association between harmonized brain responses and behavioral placebo analgesia; MDE is shown as partial correlation r after residualizing the same covariates as in the main model. (C) Indirect (a×b): mediation (ACME) effect; MDE is shown in VAS points. For each voxel and path, MDE at level α was defined as the two-sided detectability threshold $MDE_v(\alpha) = Q_{1-\alpha}(|E_{null,v}|)$, i.e., the 1-$\alpha$ quantile of the absolute effect under the sign-flip null distribution (1,000 sign-flip samples), generated by randomly flipping the sign of participant placebo–control contrast maps and rerunning the full harmonization and mediation model (including study regressors orthogonalized to induction type and covariates age, sex, and control pain). Axial slices (z-coordinates shown) display MDE maps for α = .01, and .001 (rows); lower MDE values indicate higher sensitivity. To clarify any remaining risks to power and inference, we quantified voxel-wise MDEs for each inference path from the corresponding sign-flip null distributions. For two-sided significance level p < .01 (the cluster-forming threshold used to visualize our results), the median MDE across all brain voxels was β = 6.5% in terms of percentile points for Path A (on the effect-size scale used in the manuscript), r = .131 for Path B (correlation), and a×b = 0.249 for the indirect (mediation) effect. In the manuscript tables X-Y reporting p < .01 clusters with FDR-significant peaks, the corresponding absolute peak effects ranged from β = 5.4-10.7, |r| = .11-.18, and a×b = 0.2-0.6, robustly*

exceeding the corresponding detectability thresholds and including peaks detectable even at α = .01 and α = .001. Detectability thresholds were remarkably homogenous across brain voxels and inference paths, with slightly higher thresholds in cerebellar and orbitofrontal regions (overall maximal MDE values: β = 13.0%, r = .17, $a \times b$ = 0.68), suggesting that in certain parts of these regions, our study may have missed small effects. In sum, the resulting MDE maps indicate high sensitivity across most brain voxels, with reduced detectability largely confined to peripheral cerebellar and orbitofrontal regions, consistent with susceptibility-related fMRI signal dropout.

Supplementary Tables

Supplementary Table S1. *Types of conditioning separate for each included study.*

Study	Induction type	Treatment	Cond type	Cond session	Cond length
Atlas et al. (2012)		IV-infusion	NA	NA	NA
Bingel et al. (2006)	V + C	Topical cream	Response	Same session	2x4 stimuli per hand
Bingel et al. (2011)	V + C	IV-infusion	Response	Extra session[a]	No info
Choi et al. (2011)	V + C	IV-infusion	Response	Same session	No info
Eippert et al. (2009)	V + C	Topical cream	Response	Extra & same session[b]	6 stimuli per session
Ellingsen et al. (2013)	V	Nasal spray	NA	NA	NA
Elsenbruch et al. (2012)	V	IV-infusion	NA	NA	NA
Freeman et al. (2015)	V + C	Topical cream	Response	Extra session[c]	3 stimuli
Geuter et al. (2013)	V + C	Topical cream	Response	Same session[d]	12 stimuli[e]
Kong et al. (2006)	V + C	Sham acupuncture	Response	Extra & same session[f]	6/12 stimuli per session
Kong et al. (2009)	V + C	Sham acupuncture	Response	Extra & same session[g]	8/6 stimuli per session
Lui et al. (2010)	V + C	Sham TENS	Response	Same session	2x12 stimuli
Schenk et al. (2014)	V	Topical cream	NA	NA	NA
Theysohn et al. (2014)	V	IV-infusion	NA	NA	NA
Wrobel et al. (2014)	V + C	Topical cream	Response	Extra & same session[h]	12/6 stimuli per session
Zeidan et al. (2015)	V + C	Topical cream	Response	Extra sessions[i]	10 stimuli per session

Note. IV = intra-venous; TENS = transcutaneous electrical nerve stimulation; V = verbal; C = conditioning; NA = not applicable; Response = response conditioning where pain is lowered in placebo condition. [a] Cond session min. 24hrs before test session. [b] Cond on consecutive Day 1 in the lab and Day 2 in the scanner before test session. [c] Sessions separated by 2-14 days. [d] Two sessions around one week apart for weak and strong placebo. [e] Six stimuli each outside and inside the scanner. [f] Sessions min. four days apart. [g] Sessions min. four days apart. [h] Cond on consecutive Day 1 and Day 2 outside of the scanner. [i] Four-day placebo conditioning.

Supplementary Table S2. *Summary of included within-participant placebo neuroimaging studies.*

Study	Sample size (*n*)	Mean age (years)	Sex (% male)	Pain stimulus	Induction type	Treatment
Atlas et al. (2012)	21	25	48	Heat	Verbal	IV-infusion
Bingel et al. (2006)	19	24	79	Laser	Verbal + Cond	Topical cream
Bingel et al. (2011)	22	28	68	Heat	Verbal + Cond	IV-infusion
Choi et al. (2011)	15	25	100	Electro	Verbal + Cond	IV-infusion
Eippert et al. (2009)	40	26	100	Heat	Verbal + Cond	Topical cream
Ellingsen et al. (2013)	28	26	68	Heat	Verbal	Nasal spray
Elsenbruch et al. (2012)	36	26	42	Distension	Verbal	IV-infusion
Freeman et al. (2015)	24	27	50	Heat	Verbal + Cond	Topical cream
Geuter et al. (2013)	40	26	100	Heat	Verbal + Cond	Topical cream
Kong et al. (2006)	10	27	60	Heat	Verbal + Cond	Sham acupuncture
Kong et al. (2009)	12[a]	26	42	Heat	Verbal + Cond	Sham acupuncture
Lui et al. (2010)	31	23	45	Laser	Verbal + Cond	Sham TENS
Schenk et al. (2014)	32	26	53	Cap + Heat	Verbal	Topical cream
Theysohn et al. (2014)	30	35	50	Distension	Verbal	IV-infusion
Wrobel et al. (2014)	38	26	58	Heat	Verbal + Cond	Topical cream
Zeidan et al. (2015)	17[a]	28	47	Heat	Verbal + Cond	Topical cream

Note. Total *n* = 415 participants (409 after excluding individuals with missing pain ratings); Cap = capsaicin; IV = intra-venous; TENS = transcutaneous electrical nerve stimulation; Cond = conditioning. [a] Multi-arm trial; only the placebo arm was retained, which included within-person placebo and control conditions.

Supplementary Table S3. *Study screening, eligibility checking, and retrieval.*

#	First author	Year	PMID	Source	Comment	n
**Eligible, included**						
1	Atlas	2012	22674280	MA	Included	21

2	Bingel	2006	16364549	MS	Included	19
3	Bingel	2011	21325618	MA	Included	22
4	Choi	2011	21546858	MS	Included	15
5	Eippert	2009	19709634	MS	Included	40
6	Ellingsen	2013	24127578	MS	Included	28
7	Elsenbruch	2012	22136749	MS	Included	36
8	Freeman	2015	25776211	Rec	Included	24
9	Geuter	2013	23201367	MS	Included	48
10	Lui	2010	20943318	MS	Included	33
11	Kessner	2014	25275613	MS	Included	39
12	Kong	2006	16407533	MS	Included	16
13	Kong	2009	19159691	MS	Included	12
14	Rütgen	2015	26417092	Rec	Included	102
15	Schenk	2014	24076046	Rec	Included	32
16	Theysohn	2014	25346054	MS	Included	33
17	Wager (Study I)	2004	14976306	MS	Included	25
18	Wager (Study II)	2004	14976306	MS	Included	24
19	Wrobel	2014	24796219	MS	Included	44
20	Zeidan	2015	26586819	Rec	Included	20
**Eligible, not available**						
21	Craggs	2014	24412799	MS	Responded, data unavailable	15
22	Lee	2012	22541443	MS	No response	34
23	Lu	2010	19962240	MS	No response	14
24	Nemoto	2007	17287994	MA	No response	10
25	Petrovic	2002	11834781	MA	Responded, data unavailable	9
26	Price	2007	16963184	MS	Responded, data unavailable	9
27	Sevel	2015	25659463	MS	Responded, data unavailable	24
28	Watson	2009	19523766	MS	Responded, data unavailable	11
**Eligible, published after study-search**						
29	Fehse	2015	25933389	PS	Not sought	30
30	Schenk	2017	28883019	PS	Not sought	48
31	van der Meulen	2017	28338955	PS	Not sought	30
32	Gollub	2018	29325883	PS	Not sought	45
33	Linnman	2018	29255671	PS	Not sought	18
34	Yue	2018	29025005	PS	Not sought	25
**Assessed for eligibility, not eligible**						
35	Chae	2009	19533753	MS	Placebo & pain conditions not separable	na
36	Craggs	2007	17904390	MS	Re-analysis of 16963184	na
37	Craggs	2008	18804916	MS	Re-analysis of 16963184	na
38	Eippert	2009	19833962	MS	Spinal	na
39	Jensen	2014	25452576	MS	No treatment context (cued expectancy)	na
40	Kotsis	2012	22747652	MS	Re-analysis of 22136749	na
4	Leech	2013	24093551	MS	No experimental pain (cough)	na
42	Huber	2013	23664683	MS	Re-analysis of 20943318	na
43	Petrovic	2010	20399560	MS	Re-analysis of 11834781	na
44	Schmid	2015	24833636	MS	Re-analysis of 25346054	na
45	Wager	2011	21228154	MS	Re-analysis of 14976306	na
46	Zhang	2013	23123362	MS	No experimental pain in fMRI	na
**Screened, not eligible**						
47	Amanzio	2013	22125184	MS	Review/comment	na
48	Beauregard	2009	19023697	MS	Review/comment	na
49	Benedetti	2007	17379417	MS	Review/comment	na
50	Berna	2011	21815494	MS	Review/comment	na
51	Bingel	2010	20376600	MS	Review/comment	na

52	Blom	2011	21734437	MS	No experimental placebo, no fMRI	na
53	Büchel	2014	24656247	MS	Review/comment	na
54	Colloca	2008	17960416	MS	Review/comment	na
55	Columbo	2015	25758451	MS	No experimental placebo, no fMRI	na
56	Dalakas	1995	7611640	MS	No experimental placebo, no fMRI	na
57	Dobrila-Dintinjana	2011	22220463	MS	Review/comment	na
58	Dukart	2014	24379394	MS	No experimental placebo	na
59	Gamus	2015	25796668	MS	Review/comment	na
60	Ghahreman	2011	21539702	MS	No experimental placebo, no fMRI	na
61	Grabowski	2010	20677441	MS	Review/comment	na
62	Gupta	2011	21250799	MS	No experimental placebo, no fMRI	na
63	Hashmi	2012	22531485	MS	No experimental pain	na
64	Hashmi	2012	22985900	MS	No experimental pain	na
65	Höller	2009	19573501	MS	Review/comment	na
66	Howell	2010	20839687	MS	No experimental placebo, no fMRI	na
67	Hróbjartsson	2011	21524568	MS	Review/comment	na
68	Johnson	2004	15134003	MS	Review/comment	na
69	Khalili-Mahani	2015	25554429	MS	No experimental pain in fMRI	na
70	Kong	2007	18019605	MS	Review/comment	na
71	Li	2010	21280461	MS	Review/comment	na
72	Li	2014	24817188	MS	No experimental placebo	na
73	Lidstone	2007	17334853	MS	Review/comment	na
74	Lu	2011	21751434	MS	Review/comment	na
75	Martini	2015	25523008	MS	No fMRI	na
76	Miura	2013	23711332	MS	No experimental pain	na
77	Murray	2013	23880289	MS	Review/comment	na
78	Nandhagopal	2008	18413571	MS	Review/comment	na
79	Petersen	2014	25281929	MS	No fMRI	na
80	Petrovic	2005	15953423	MS	No experimental pain	na
81	Qiu	2009	19784082	MS	Review/comment	na
82	Rainville	2006	16513275	MS	Review/comment	na
83	Rigatelli	2008	18759545	MS	Review/comment	na
84	Ritter	2014	24672009	MS	No experimental placebo	na
85	Sant'Anna	2014	25372920	MS	No experimental placebo	na
86	Sarinopoulos	2006	16472720	MS	No experimental pain	na
87	Scott	2007	17640532	MS	No experimental pain in fMRI	na
88	Scott	2008	18250260	MA	Pharmacological PET	na
89	Stein	2012	22959599	MS	No experimental placebo	na
90	Su	2010	21290837	MS	Review/comment	na
91	Theis	2004	15354245	MS	Review/comment	na
92	Wager	2007	17578917	MA	Pharmacological PET	na
93	Wager	2013	24761154	MS	Review/comment	na
94	Werndle	2015	24819624	MS	No experimental placebo	na
95	Wiech	2014	25093555	MS	No fMRI	na
96	Wu	2014	24268723	MS	No experimental placebo	na
97	Xu	2014	25069206	MS	Review/comment	na
98	Yelle	2009	19692600	MS	No experimental placebo	na
99	Yilmaz	2010	20817354	MS	No experimental placebo	na
100	Yu	2014	24578196	MS	No experimental pain	na
101	Zhang	2011	21332487	MS	No experimental pain	na
102	Zubieta	2009	19338509	MS	Review/comment	na

Note. The *n* shown for eligible studies refer to participants that completed testing according to the original manuscripts. Abbreviations: fMRI = functional Magnetic Resonance Imaging; MS = study identified in an initial medline search; na = not assessed; MA = study identified in previous meta-analyses; PS = study identified in post-hoc search; Rec = study added late after recommendation by collaborators during data acquisition,

Supplementary Table S4. *Risk of bias assessment for NPS response according to the Cochrane risk-of-bias tool.*

			Selection Bias	Performance Bias	Detection Bias	Attrition Bias			Sequence Bias	
**#**	**First author**	**Date**	**Allocation to treatment**	**Blinding of subjects and treatment providers**	**Analyst blinding**	**subjects available / entered testing (%)**	**subjects available / completed testing (%)**	**subjects available / included in original analysis (%)**	**Sequence of placebo sessions per protocol**	**% participants where control was tested before placebo**
1	Atlas	2012	WIS	No, but unlikely to confound effects of induction type	No, but study did not analyze the effect of induction type directly	87.5	100.0	100.0	balanced	42.9
2	Bingel	2006	WIS			95.0	100.0	100.0	alternating	50.0
3	Bingel	2011	WIS			95.7	100.0	100.0	pre-post	100.0
4	Choi	2011	WIS			100.0	100.0	100.0	?	?
5	Eippert	2009	WIS			83.3	100.0	100.0	balanced	55.0
6	Ellingsen	2013	WIS			93.3	100.0	100.0	balanced	53.6
7	Elsenbruch	2012	WIS			100.0	100.0	100.0	balanced	55.6
8	Freeman	2015	WIS			63.2	100.0	100.0	alternating	50.0
9	Geuter	2013	WIS			76.9	83.3	100.0	balanced	46.3
11	Kong	2006	WIS	Blinded subjects		41.7	62.5	62.5	alternating	50.0
12	Kong	2009	WIS	No, but unlikely to confound effects of induction type		?	?	100.0	alternating	50.0
13	Lui	2010	WIS			86.1	93.9	100.0	alternating	50.0
15	Schenk	2014	WIS			82.1	100.0	100.0	balanced	53.1
16	Theysohn	2014	WIS			83.3	90.9	100.0	balanced	60.0
19	Wrobel	2014	WIS			76.0	86.4	100.0	balanced	42.1
20	Zeidan	2015	WIS			85.0	85.0	89.5	pre-post	100.0
Total			-	-	-	84.4[1]	95.2[1]	98.7	-	54.1[1]

Note. Red cells denote parameters indicating high risk of bias, yellow cells unknown risk of bias and green cells low risk. * placebo responder pre-selection was performed amongst participants. Abbreviations: ? = unknown/missing data; [1] excluding studies with unknown values; NA = not applicable; NPS = neurologic pain signature; WIS = within-subject study design.

Supplementary Table S5. *Full table with regression coefficients, standard errors, T-scores, p-values and confidence intervals for the statistical test modelling the effect of induction type on behavioral placebo analgesia.*

	coef	***std err***	***T***	***p(|T|>0)***	***p(T>0)***	***95% CI (coef)***	
intercept	11.1459	0.774	14.394		**< .0001**	9.623	12.668
placebo induction	1.3394	0.807	1.660		**.049**	-0.247	2.926
control pain rating	0.3049	0.041	7.404	**< .0001**		0.224	0.386
age	-0.2898	0.146	-1.981	**.048**		-0.577	-0.002
sex	2.2475	1.920	1.170	.243		-1.528	6.023
Atlas	-0.1930	0.677	-0.285	.776		-1.523	1.137
Bingel 06	2.1391	0.721	2.966	**.003**		0.721	3.557
Bingel 11	0.8278	0.713	1.161	.246		-0.574	2.230
Choi	-0.7059	0.738	-0.957	.339		-2.157	0.745
Eippert	0.3153	0.686	0.460	.646		-1.033	1.664
Ellingsen	0.5167	0.635	0.813	.416		-0.732	1.766
Elsenbruch	0.2861	0.598	0.479	.632		-0.889	1.461
Freeman	0.5094	0.710	0.717	.474		-0.887	1.906
Geuter	-1.7419	0.690	-2.525	**.012**		-3.098	-0.385
Kong 06	-1.7226	0.754	-2.284	**.023**		-3.206	-0.240
Kong 09	-1.4959	0.752	-1.989	**.047**		-2.974	-0.018
Lui	1.2096	0.766	1.579	.115		-0.297	2.716
Schenk	-1.6031	0.611	-2.625	**.009**		-2.804	-0.402

Theysohn	0.9933	0.667	1.489	.137	-0.318	2.305
Wrobel	0.1456	0.683	0.213	.831	-1.196	1.488
Zeidan	-0.2074	0.734	-0.282	.778	-1.651	1.236

Note. Bold values indicate statistically significant two-sided p *values (*p *< 0.05); multiple-testing correction was not applied.*

Supplementary Table S6. *Results of the random effect model that explains placebo rating differences with age, sex and control pain rating as fixed effects and study as random effect.*

	coef	***std err***	***z***	***p(|T|>0)***	***95% CI (coef)***	
intercept	11.052	1.267	8.726	**< .0001**	8.570	13.535
sex	2.310	1.832	1.261	.207	-1.281	5.901
age	-0.301	0.140	-2.146	**.032**	-0.576	-0.026
control pain rating	0.273	0.041	6.728	**< .0001**	0.193	0.352
study	15.044	0.653				

Note. The residuals of this model were subject to Levene's test of equal variances to compare INST vs. COND+INST studies. Bold values indicate statistically significant two-sided *p* values (*p* < 0.05); multiple-testing correction was not applied.

Supplementary Table S7. *Regions with statistically significant placebo response, pooled across all participants, regardless of induction type.*

Cluster Size (mm^3)	***X***	***Y***	***Z***	***Effect***	***Peak Stat uncorr***	***Peak Stat FDR***	***Mars region***	***yeo_network***
416	54	30	24	0.098	< .0001	< .0001	R Caudal DLPFC	Cont
328	46	-70	38	0.088	< .0001	< .0001	R Superior Visual Cortex	Default
512	46	-56	36	0.102	< .0001	< .0001	R Dorsal Inferior Parietal Cortex	Cont
1256	38	2	12	-0.089	< .0001	< .0001	R Ventral Motor Cortex	SalVentAttn
1176	28	-26	60	-0.103	< .0001	< .0001	R Dorsomedial S1	SomMot
696	28	12	2	-0.113	< .0001	< .0001	R Putamen	N/A
320	16	66	14	0.128	< .0001	< .0001	R Rostral DLPFC	Cont
576	16	-10	66	-0.085	< .0001	< .0001	R Dorsomedial M1	SomMot
896	12	-86	16	-0.077	< .0001	< .0001	R Caudal Medial Visual Cortex	VisCent
448	10	-6	54	-0.106	< .0001	< .0001	R Dorsomedial M1	Cont
496	-6	-36	10	-0.100	< .0001	< .0001	L Isthmus Cingulate Cortex	N/A
736	-24	-34	64	-0.098	< .0001	< .0001	L Dorsomedial S1	SomMot
840	-22	0	2	-0.099	< .0001	< .0001	L Pallidum	N/A
	-28	-6	8	-0.100	< .0001	< .0001	L Putamen	N/A
632	-38	54	-2	0.111	< .0001	< .0001	L Rostral DLPFC	Cont
368	38	-10	40	-0.101	< .0001	.001	Right Dorsolateral M1	SomMot

Note. Only cluster peaks surviving FDR-correction and with a minimum distance of 20 mm to adjacent clusters and a minimum cluster extent of 40mm^3 are reported. X, Y and Z coordinates of the peak activation are reported in the MNI152 space, in units of mm. Regions are identified with the Mars brain atlas [17] and the Yeo 7-network functional parcellation [18], based on the MNI-coordinates of the peak. Abbreviations: DorsAttn = dorsal attention network, Default = default mode network, SomMot = somatomotor network, Cont = frontoparietal executive control network, Vis = visual network, SalVentAttn = salience and ventral attention network.

Supplementary Table S8. *Habenula region-of-interest (ROI) analysis.*

	left habenula		***right habenula***	
	**coef**	***p***	**coef**	***p***
INST mean	-0.051	**.016**	-0.026	.229
COND+INST mean	-0.053	**.046**	-1.75	.154
Path A	-0.002	.899	-0.017	.451
Path B	0.351	.878	-1.75	.534
Mediation effect	0.004	.853	0.03	.432

Note. Bold values indicate statistically significant two-sided p *values (*p *< 0.05); multiple-testing correction was not applied.*

The habenula has previously also been implicated in pain modulation and placebo mechanisms [19,20]. We obtained the habenula ROI from the subcortical brain atlas of [21] and divided it into bilateral components (by x = 0). Within all voxels of each ROI, we averaged the voxel-wise path a, path b and mediation (a*b) regression coefficients, as well as the group-mean estimates for INST and COND+INST (see Mediation Analysis in the main manuscript). To construct the null distribution for statistical inference, we used the ROI-wide averages of the voxel-wise null-distribution estimates, calculated based on sign-flipped 10 000 surrogate datasets, separately for path a, b, a*b and the INST and COND+INST group means. Similarly to the voxel-wise analysis, we calculated *p*-values by fitting a generalized Pareto distribution on the tail of the null distributions. We found that activity in the left habenula showed a significant placebo-related decrease with INST (harmonized effect size difference: -5.1%, *p* = .016) and COND+INST (-5.3%, *p* = .046), replicating our previous findings [19]. No statistically significant effects were observed in the right habenula. The ROI analysis also found no significant path a, path b, or mediation effects in either the left or right habenula.

Supplementary Table S9. *Regions with statistically significant sex differences.*

Cluster Size (mm^3)	*X*	*Y*	*Z*	*beta*	*qFDR*	*Region*	*Network*
528	-42	18	8	-0.136	< 0.0001	L anterior insula	SalVentAttn
800	4	16	58	-0.119	< 0.0001	R DMPFC	Cont
40	-20	-12	-8	-0.117	0.0129	L Pallidum	N/A
56	54	-28	-20	-0.114	< 0.0001	R Rostral ITG	Cont
128	62	6	16	-0.114	< 0.0001	R M1	SomMot
88	44	-54	4	-0.109	0.04906	R Caudal MTG	Vis
120	36	-54	56	-0.107	< 0.0001	R Superior Parietal Cortex	DorsAttn
72	16	14	28	-0.104	< 0.0001	R MCC	Cont
176	8	2	54	-0.102	< 0.0001	R DMPFC	SalVentAttn
280	-54	-50	-12	-0.097	< 0.0001	L Lateral Visual Cortex	DorsAttn
24	-16	26	-16	-0.096	< 0.0001	L Ventromedial OFC	Cont
56	58	-14	48	-0.096	< 0.0001	R Dorsolateral S1	SomMot

Note. Only cluster peaks surviving FDR-correction and with a minimum distance of 20 mm to adjacent clusters are reported. X, Y and Z coordinates of the peak activation are reported in the MNI152 space, in units of mm. Regions are identified with the Mars brain atlas [17] and the Yeo 7-network functional parcellation [18], based on the MNI-coordinates of the peak. Abbreviations: L = left, R = right, DorsAttn = dorsal attention network, Default = default mode network, SomMot = somatomotor network, Cont = frontoparietal executive control network, Vis = visual network, SalVentAttn = salience and ventral attention network.

Supplementary Table S10. *Regions with statistically significant age effects.*

Cluster Size (mm^3)	*X*	*Y*	*Z*	*Beta*	*Placebo response*	*q_{FDR}*	*r*	*Region*	*Network*
8	12	-44	-42	0.007	-0.026	<0.0001	0.143	R Cerebellum	N/A
8	-4	-54	68	0.008	0.057	<0.0001	0.091	L Precuneus	DorsAttn
248	-12	-42	24	0.008	-0.042	<0.0001	0.106	L Posterior Cingulate Cortex	Cont
16	2	-58	36	0.008	0.004	0.00377	0.116	R Posterior Cingulate Cortex	Default
24	-2	-14	28	0.009	-0.021	0.0038	0.202	Left Mid Cingulate Cortex	Cont

Note. Only cluster peaks surviving FDR-correction and with a minimum distance of 20 mm to adjacent clusters are reported. X, Y and Z coordinates of the peak activation are reported in the MNI152 space, in units of mm. Regions are identified with the Mars brain atlas [17] and the Yeo 7-network functional parcellation [18], based on the MNI-coordinates of the peak. Abbreviations: L = left, R = right, DorsAttn = dorsal attention network, Default = default mode network, Cont = frontoparietal executive control network.

Supplementary Table S11. *Regions with statistically significant stimulation side differences.*

Cluster Size (mm³)	***X***	***Y***	***Z***	***Diff.***	***qFDR***	***Region***	***Network***
176	22	-50	38	-11.0	< 0.0001	R Medial Parietal Cortex	Cont
176	46	-52	-8	-15.9	< 0.0001	R Lateral Visual Cortex	DorsAttn
88	-16	-86	-2	-10.1	< 0.0001	L Rostral Medial Visual Cortex	Vis
104	24	-48	8	-13.0	< 0.0001	R Rostral Medial Visual Cortex	Cont
160	-32	-48	0	-13.3	< 0.0001	L Rostral Medial Visual Cortex	Cont
24	12	-14	32	-12.3	< 0.0001	R MCC	Cont
64	10	-40	18	-8.8	< 0.0001	R PCC	Cont
32	-12	-24	20	-8.9	< 0.0001	L Thalamus	N/A
216	16	-28	28	-8.9	< 0.0001	R PCC	Cont
64	14	-20	64	-2.2	< 0.0001	R Dorsomedial S1	Cont
192	-28	-46	20	-14.8	< 0.0001	L Ventral Inferior Parietal Cortex	N/A
48	-38	-40	20	-5.9	< 0.0001	L Ventral Inferior Parietal Cortex	Cont
360	32	-50	12	-9.4	< 0.0001	R Rostral Medial Visual Cortex	Cont
24	38	-24	24	-4.4	< 0.0001	R Ventral IPC	SomMot
216	20	-42	60	-9.1	< 0.0001	R Superior Parietal Cortex	SomMot
152	-52	10	-10	9.7	< 0.0001	L Rostral STG	SalVentAttn
48	26	-24	12	-2.6	< 0.0001	R Thalamus	N/A
24	32	-66	22	4.2	< 0.0001	R Superior Visual Cortex	Cont
64	-14	-68	48	14.3	< 0.0001	L Medial Superior Parietal Cortex	DorsAttn
32	-14	-66	-30	-2.1	< 0.0001	L Rostral Medial Visual Cortex	N/A
32	-38	42	28	8.0	0.00121	L Rostral Inferior DLPFC	SalVentAttn
56	20	56	-6	8.3	< 0.0001	R Ventral OFC	Cont
544	-44	-42	52	9.3	< 0.0001	L Superior Parietal Cortex	SomMot
216	18	-86	-38	10.3	< 0.0001	R Caudal Medial Visual Cortex	N/A
80	-16	-6	-18	10.9	< 0.0001	Left Amygdala	N/A
96	-42	-64	4	9.1	< 0.0001	Left Lateral Visual Cortex	DorsAttn
64	-36	-18	60	4.7	0.00085	Left Dorsolateral M1	Cont
64	-8	44	28	11.7	< 0.0001	Left Rostral DMPFC	Default
56	-38	-4	-40	7.4	< 0.0001	L Rostral ITG	Cont
40	-18	0	42	9.0	< 0.0001	Left Dorsolateral Premotor Cortex	Cont

Note. Only cluster peaks surviving FDR-correction and with a minimum distance of 20 mm to adjacent clusters are reported. X, Y and Z coordinates of the peak activation are reported in the MNI152 space, in units of mm. Regions are identified with the Mars brain atlas [17] and the Yeo 7-network functional parcellation [18], based on the MNI-coordinates of the peak. Abbreviations: L = left, R = right, DorsAttn = dorsal attention network, Default = default mode network, SomMot = somatomotor network, Cont = frontoparietal executive control network, Vis = visual network, SalVentAttn = salience and ventral attention network.

Supplementary Table S12. *Full table including the regression coefficients, standard errors, T-scores, p-values and confidence intervals for the statistical test modelling the interaction effect between induction type and behavioral analgesia on NPS scores. Bold values indicate statistically significant two-sided p values (p < 0.05); multiple-testing correction was not applied.*

	coef	***std err***	***T***	***p***	***95% CI (coef)***	
intercept	0.0011	0.002	0.670	.72	-0.0026	0.0047
placebo induction	5.17e-05	0.002	0.028	.52	-0.0037	0.00386
analgesia	-0.0004	0.001	-3.559	**.0005**	-0.0006	-0.00016
analgesia:induction	-0.0002	8.89e-05	-1.825	**.034**	-0.000344	-0.000001
control pain rating	-6.414e-05	8.42e-05	-0.762	.21	-0.0002	0.0001
age	-0.0006	0.001	-2.060	**.014**	-0.001	-0.0001
sex	0.0001	0.004	0.040	.51	-0.0069	0.0072
Atlas	0.0007	0.001	0.566	.572	-0.002	0.003
Bingel 06	-0.0013	0.001	-0.918	.359	-0.004	0.001
Bingel 11	0.0011	0.001	0.808	.420	-0.002	0.004
Choi	-0.0018	0.001	-1.259	.209	-0.005	0.001
Eippert	0.0010	0.001	0.791	.429	-0.002	0.004

Ellingsen	0.0004	0.001	0.360	.719	-0.002	0.003
Elsenbruch	-0.0014	0.001	-1.215	.225	-0.004	0.001
Freeman	0.0034	0.001	2.499	**.013**	0.001	0.006
Geuter	0.0021	0.001	1.566	.118	-0.001	0.005
Kong 06	0.0012	0.001	0.856	.393	-0.002	0.004
Kong 09	-0.0011	0.001	-0.767	.444	-0.004	0.002
Lui	-0.0003	0.001	-0.209	.834	-0.003	0.003
Schenk	-0.0021	0.001	-1.753	.080	-0.004	0.000
Theysohn	0.0026	0.001	2.006	**.046**	5.11e-05	0.005
Wrobel	-0.0028	0.001	-2.126	**.034**	-0.005	-0.000
Zeidan	-0.0026	0.001	-1.836	.067	-0.005	0.000

Supplementary Table S13. *Full table including the regression coefficients, standard errors, T-scores, p-values and confidence intervals for the statistical test modelling the interaction effect between induction type and behavioral analgesia on SIIPS scores. Bold values indicate statistically significant two-sided p values (p < 0.05); multiple-testing correction was not applied.*

	coef	***std err***	***T***	***p***	***95% CI (coef)***	
intercept	0.0063	0.004	-1.455	.074	-0.015	0.0023
placebo induction	-0.0017	0.004	-0.419	.359	-0.010	0.0065
analgesia	-0.0008	0.001	-3.208	**.004**	-0.0013	-0.00019
analgesia:induction	-0.0002	0.001	-1.168	.145	-0.0007	0.0002
control pain rating	-9.871e-05	0.001	-0.523	.28	-0.0005	0.0003
age	-8.955e-05	0.001	-0.142	.44	-0.0011	0.0013
sex	-0.0032	0.008	-0.386	.34	-0.019	0.013
Atlas	-0.0016	0.003	-0.547	.585	-0.007	0.004
Bingel 06	-0.0039	0.003	-1.218	.224	-0.010	0.002
Bingel 11	0.0042	0.003	1.363	.174	-0.002	0.010
Choi	0.0034	0.003	1.089	.277	-0.003	0.010
Eippert	-0.0015	0.003	-0.520	.603	-0.007	0.004
Ellingsen	-0.0014	0.003	-0.512	.609	-0.007	0.004
Elsenbruch	0.0012	0.003	0.455	.649	-0.004	0.006
Freeman	0.0043	0.003	1.419	.157	-0.002	0.010
Geuter	0.0059	0.003	1.971	.049	1.32e-05	0.012
Kong 06	-0.0060	0.003	-1.836	.067	-0.012	0.000
Kong 09	-0.0032	0.003	-0.974	.331	-0.010	0.003
Lui	0.0009	0.003	0.258	.796	-0.006	0.007
Schenk	-0.0015	0.003	-0.575	.565	-0.007	0.004
Theysohn	0.0030	0.003	1.045	.297	-0.003	0.009
Wrobel	-0.0051	0.003	-1.736	.083	-0.011	0.001
Zeidan	-0.0022	0.003	-0.686	.493	-0.008	0.004

Supplementary Table S14. *Results of the statistical tests (two-sided) assessing the association between induction type and potential confounders.*

	test	***statistic***	***p***
stimulus type	χ2	8.55	.073
stimulus location	χ2	6.69	.082
stimulation side	χ2	5.47	.140
stimulus duration	Welch T	1.80	.12
repetition time (TR)	Welch T	-0.94	.367
echo time (TE)	Welch T	0.37	.722

Supplementary Methods

1. Original study identification

Studies were identified through the following sources:

a) an initial online-search of the electronic bibliographic database MEDLINE via PubMed on May 21st 2015 using the search term: *((placebo effect[Title/Abstract]) OR placebo analgesia[Title/Abstract]) AND fMRI OR PET.*
b) by enriching initial search results with studies identified in an earlier meta-analysis of author TDW. [22,23]. Search results in these preceding peak-voxel-based meta-analyses were obtained by "identified using literature searches in PubMed and Google Scholar, the authors' personal libraries, and examining references of relevant papers."
c) through recommendations by collaborating investigators.

Studies identified are listed in **Supplementary Table S11**, the data-acquisition process is illustrated in **Supplementary Figure S8**.

Authors MZ, UB, and TDW screened the titles and abstracts of all records retrieved; studies that provisionally met eligibility criteria were assessed for eligibility by examining the full text. Study eligibility was determined in a joint discussion of authors MZ, UB, and TDW. Agreement between reviewers was accomplished in a joint discussion. There were no studies where the decision for inclusion/exclusion was a matter of ambiguity (see **Supplementary Table S12**).

2. Post-hoc study identification

An exploratory post-hoc literature search was performed on March 10th 2018 to account for the fact that considerable time had passed between the initial study search and the completion of the meta-analysis. We searched pubmed and Thomson Reuters Web of Science from the beginning of 2015 to the present day using the following (extended) search terms:

Pubmed:
(placebo effect OR "placebo analgesia" OR "placebo effect"[MeSH]) AND ("functional magnetic resonance imaging" OR fMRI OR PET OR "functional neuroimaging" OR ASL OR fMRI[MeSH] OR "functional neuroimaging"[MeSH]) AND (pain OR pain[MeSH] OR analgesia OR noci*) NOT (Review[Filter] OR Editorial[Filter] OR Comment[Filter])

Web of Science (WoS, searching: all databases):
TS=("placebo effect" OR "placebo analgesia") AND TS=(pain OR analgesia OR noci*) AND TS=("functional magnetic resonance imaging" OR fMRI OR POET OR "functional neuroimaging" OR ASL)

Refined by: [excluding] DOCUMENT TYPES: (REVIEW OR EDITORIAL OR CASE REPORT)

After removing duplicates, author MZ screened titles abstracts and assessed full-texts for eligibility. The post-hoc analysis indicated that at least six eligible studies [24–29] (with a total *N* of 196) were published after the initial study search in 2015 and therefore missed by the present meta-analysis (**Supplementary Table S12**).

3. Risk-of-bias assessment

Aim of this risk-of-bias assessment was to assess the studies included in our meta-analysis in terms of internal validity, regarding the effect of placebo interventions on (a) pain ratings and neural responses. Author MZ evaluated each study with respect to selection bias, performance bias, attrition bias, detection bias, report bias, and biases introduced by the use of within-subject designs (sequence effects) using to the Cochrane risk of bias tool [30]. All judgments were based on single-subject raw data, information taken from the published manuscripts, or personal communication with the study authors, following this order of priority.

Selection bias

Problems due to non-random allocation are considered minor in within-subject designs as all participants undergo both treatments [31]. Therefore, risk of selection bias was low in most studies included, for both pain ratings and neural responses. In summary, selection bias due to non-random allocation of participants to placebo/control conditions was judged as low for both ratings and neural responses in most studies (**Supplementary Table 12**).

Performance bias

Awareness of the allocated experimental condition by participants and personnel is considered the major source of performance bias in clinical trials [30]. However, the issue of blinding in experimental placebo research is different than in standard drug and device studies: Giving participants instructions that they are being treated and suggestions about treatment efficacy are core features of the placebo manipulation itself [32]. Further, the treatment provider and her/his behavior are considered major factors driving the placebo effect [33]. Placebo studies with blinded study participants or treatment providers [32] may underestimate the placebo effects typical for clinical settings. On the other hand, the fact that full blinding is conceptually difficult in experimental placebo studies does not imply that performance bias is not a problem [32]. The lack of blinding in placebo studies makes it difficult to discern placebo effects on core experiences and symptoms from placebo effects on participants' judgments and reporting behavior [32]. Thus, so-called "demand characteristics" (participants' tendency to report what they believe they *should* report, independent of experience) and other biases in judgment and decision-making can influence behavioral placebo effects, which is a major reason to also examine physiological outcomes.

None of the included studies blinded participants or treatment providers (**Supplementary Table S3**), with the exception of one between-group study that blinded subjects in respect to group allocation [34]. The risk that pain ratings overestimate placebo effects on processes that contribute to core pain experiences (independent of judgments) is therefore high. However, the effect of induction type on behavioral and neural measures is likely free of "demand characteristics". It is therefore unlikely that a lack of blinding affected induction type related differences systematically. We therefore conclude that the risk for performance bias in our main analyses is low.

Detection bias

It is a common problem in neuroimaging research that image pre-processing pipelines and statistical analysis involve numerous analysis choices. These do not only tempt analysts to cherry-pick favorable results (i.e., "*p*-hacking"), but also pose a multiple comparison problem [35]. Blinding of analysts to the nature of experimental conditions and pre-specification of analysis parameters reduces this type of bias.

No included study reported blinding of its analysts (**Supplementary Table S12**). We therefore judged the risk for detection bias as high for pain ratings, since allocated interventions and pain ratings were available to the original assessors. Moreover, the pre-processing pipelines and first-level models of imaging analyses varied considerably (**Supplementary Table S12**). Since our meta-analysis relies on the original first-level analyses, choices by the original analysts may affect neural responses. It is however unlikely that choices in the original imaging analysis pipelines biased neural effects of induction type. We therefore judge the chance for over-estimating placebo effects on brain responses due to detection bias as low.

Attrition bias

Study drop-out and exclusion of participants may systematically affect study outcomes, especially when one experimental condition is affected more than another, or when participants are selected based on outcomes. **Supplementary Table S12** provides a general overview on the amount of missing imaging data in respect to different experimental stages of the original studies. For one study [11] insufficient information was available to determine the amount of missing data. For the remaining studies, we found that our meta-analysis included 84% of participants included in the original studies before exclusions, 95% of participants successfully completing fMRI testing, and 99% of subjects included in the original analysis. Main reasons for the discrepancy between participants tested and participants completing measurements were problems with neuroimaging and pain stimulation equipment, which are unlikely to affect placebo effects systematically. Main reasons for the discrepancy between participants completing measurements and participants analyzed in the original studies were exclusions due to imaging artifacts and due to excessive head movements, which are also unlikely to affect placebo effects systematically. Data from 6 out of 16 subjects in one [10] and 2 out of 19 subjects in another study [16] were unavailable due to failure of data-storage media. Missing data for pain ratings was

also missing for brain analyses, with the exception of Atlas et al. 2012[1] where pain ratings, but not imaging data, were lost for two subjects. Given the relatively low attrition rate and the fact that most studies are within-subject studies, where missing participants affect all experimental conditions alike, we conclude that attrition bias is unlikely to affect the outcomes of our meta-analysis.

Study reporting bias

The underreporting of studies with non-significant ("negative") results is a prevailing problem in biomedical research[36] that has been suggested to affect experimental placebo research[32]. Underreporting of studies with non-significant behavioral placebo effects may inflate the effect sizes for pain ratings of the current meta-analysis. While previously multiple meta-analyses aimed to estimate the absolute effect size of placebo analgesia across studies (e.g. [37]), which may indeed be confounded by publication biases - the aim of the current study was to elucidate the neural mechanisms underlying the placebo effect once it has been successfully induced. To investigate these mechanisms, it is necessary to include studies that observed a behavioral effect. Studies without a behavioral effect, while important for estimating overall prevalence, would be uninformative for our specific research question.

We assessed small-study effects/publication bias using funnel plots of study-specific behavioral analgesia estimates (effect size on the x-axis) against their precision (1/SE). For visualization, we overlaid the fixed effect pooled estimate with its 95% funnel and scaled point size by sample size. Funnel asymmetry was tested using Egger's regression [39] (OLS of effect/SE on precision with an intercept; two sided test of intercept = 0) and Begg & Mazumdar's rank correlation test [40] (Kendall's tau between effects and SEs). Analyses were performed separately for the INST and COND+INST subgroups, using $\alpha = 0.05$. We found no evidence of publication bias. See **Supplementary Figure S11** for details.

Other biases: unbalanced testing sequence in within-subject designs

Sequence effects (e.g. habituation or sensitization) may confound treatment-effects in within-subject designs when the order of experimental conditions is not balanced or randomized. An overview on the sequence of treatment conditions in within-subject studies is provided in **Supplementary Table S12**. Raw data on the sequence of conditions was available for all but three studies. No information about testing sequence was available for one study [4]. Several studies tested placebo and control conditions in an alternating fashion, reducing the risk of sequence confound [2,8,10,11,38]. Two studies tested placebo and control conditions in a fixed pre-placebo (control) vs. post-placebo sequence [3,16]. All remaining studies had balanced designs in respect to the sequence of placebo and control. Overall, sample imbalance for studies was low: placebo conditions were tested after control conditions in 54% of participants. Based on these figures we judged the overall risk of bias due to unbalanced sequence of testing as low.

Risk-of-bias summary

In summary, we conclude that the risk of bias is low when focusing on rating differences between INST and COND+INST. While in terms of the overall pain ratings, previously we identified high risk of bias in the same meta-analysis dataset [41], we believe it is unlikely that the overall bias would emerge as a systematic difference in responses induced with or without conditioning. For neural effects of induction type, we also conclude that the risk of bias is low. The main reason for this decision was that the neural differences between INST and COND+INST are not directly affected by reporting and communication biases.

References

1. Atlas, L. Y. *et al.* Dissociable influences of opiates and expectations on pain. *J. Neurosci.* **32**, 8053–8064 (2012).
2. Bingel, U., Lorenz, J., Schoell, E. D., Weiller, C. & Büchel, C. Mechanisms of placebo analgesia: rACC recruitment of a subcortical antinociceptive network. *Pain* **120**, 8–15 (2006).
3. Bingel, U. *et al.* The effect of treatment expectation on drug efficacy: Imaging the analgesic benefit of the opioid remifentanil. *Sci. Transl. Med.* **3**, (2011).
4. Choi, J. C. *et al.* Placebo effects on analgesia related to testosterone and premotor activation. *Neuroreport* **22**, 419–423 (2011).
5. Eippert, F. *et al.* Activation of the opioidergic descending pain control system underlies placebo analgesia. *Neuron* **63**, 533–543 (2009).
6. Ellingsen, D. M. *et al.* Placebo improves pleasure and pain through opposite modulation of sensory processing. *Proc. Natl. Acad. Sci. U. S. A.* **110**, 17993–17998 (2013).
7. Elsenbruch, S. *et al.* Neural mechanisms mediating the effects of expectation in visceral placebo analgesia: An fMRI study in healthy placebo responders and nonresponders. *Pain* **153**, 382–390 (2012).
8. Freeman, S. *et al.* Distinct neural representations of placebo and nocebo effects. *Neuroimage* **112**, 197–207 (2015).
9. Geuter, S., Eippert, F., Hindi Attar, C. & Büchel, C. Cortical and subcortical responses to high and low effective placebo treatments. *Neuroimage* **67**, 227–236 (2013).
10. Kong, J. *et al.* Brain activity associated with expectancy-enhanced placebo analgesia as measured by functional magnetic resonance imaging. *J. Neurosci.* **26**, 381–388 (2006).
11. Kong, J. *et al.* Expectancy and treatment interactions: A dissociation between acupuncture analgesia and expectancy evoked placebo analgesia. *Neuroimage* **45**, 940–949 (2009).
12. Lui, F. *et al.* Neural bases of conditioned placebo analgesia. *Pain* **151**, 816–824 (2010).
13. Schenk, L. A., Sprenger, C., Geuter, S. & Büchel, C. Expectation requires treatment to boost pain relief: An fMRI study. *Pain* **155**, 150–157 (2014).
14. Theysohn, N. *et al.* Are there sex differences in placebo analgesia during visceral pain processing? A fMRI study in healthy subjects. *Neurogastroenterol. Motil.* **26**, 1743–1753 (2014).
15. Wrobel, N., Wiech, K., Forkmann, K., Ritter, C. & Bingel, U. Haloperidol blocks

dorsal striatum activity but not analgesia in a placebo paradigm. *Cortex* **57**, 60–73 (2014).
16. Zeidan, F. *et al.* Mindfulness meditation-based pain relief employs different neural mechanisms than placebo and sham mindfulness meditation-induced analgesia. *J. Neurosci.* **35**, 15307–15325 (2015).
17. Auzias, G., Coulon, O. & Brovelli, A. MarsAtlas: A cortical parcellation atlas for functional mapping. *Hum. Brain Mapp.* **37**, 1573–1592 (2016).
18. Yeo, B. T. T. *et al.* The organization of the human cerebral cortex estimated by intrinsic functional connectivity. *J. Neurophysiol.* **106**, 1125–1165 (2011).
19. Zunhammer, M., Spisak, T., Wager, T. D., Bingel, U. & The Placebo Imaging Consortium. Meta-analysis of neural systems underlying placebo analgesia from individual participant fMRI data. *Nat. Commun.* **12**, 1391 (2021).
20. Brooks, J. & Tracey, I. From nociception to pain perception: imaging the spinal and supraspinal pathways. *J. Anat.* **207**, 19–33 (2005).
21. Pauli, W. M., Nili, A. N. & Tyszka, J. M. A high-resolution probabilistic in vivo atlas of human subcortical brain nuclei. *Sci. Data* **5**, 180063 (2018).
22. Wager, T. D. & Atlas, L. Y. The neuroscience of placebo effects: Connecting context, learning and health. *Nat. Rev. Neurosci.* **16**, 403–418 (2015).
23. Atlas, L. Y. & Wager, T. D. A meta-analysis of brain mechanisms of placebo analgesia: Consistent findings and unanswered questions. in *Handbook of Experimental Pharmacology Vol. 225. Placebo* 37–69 (Springer, 2014). doi:10.1007/978-3-662-44519-8_3.
24. Fehse, K. & Maikowski, L. Placebo responses to original vs generic ASA brands during exposure to noxious heat: a pilot fMRI study of neurofunctional correlates. *Pain Med.* **16**, 1967–1974 (2015).
25. van der Meulen, M., Kamping, S. & Anton, F. The role of cognitive reappraisal in placebo analgesia: An fMRI study. *Soc. Cogn. Affect. Neurosci.* **12**, 1128–1137 (2017).
26. Schenk, L. A., Sprenger, C., Onat, S., Colloca, L. & Büchel, C. Suppression of Striatal Prediction Errors by the Prefrontal Cortex in Placebo Hypoalgesia. *J. Neurosci.* **37**, 9715–9723 (2017).
27. Gollub, R. L. *et al.* A Functional Neuroimaging Study of Expectancy Effects on Pain Response in Patients With Knee Osteoarthritis. *J. Pain* (2018) doi:10.1016/j.jpain.2017.12.260.
28. Linnman, C. *et al.* Molecular and functional PET-fMRI measures of placebo analgesia in episodic migraine: Preliminary findings. *NeuroImage Clin.* **17**, 680–690 (2018).
29. Yue, Y. & Collaku, A. Correlation of Pain Reduction with fMRI BOLD Response in Osteoarthritis Patients Treated with Paracetamol: Randomized, Double-Blind, Crossover Clinical Efficacy Study. *Pain Med.* 355–367 (2017) doi:10.1093/pm/pnx157.
30. The Cochrane Collaboration. *Cochrane Handbook for Systematic Reviews of Interventions Version 5.1.0 [updated March 2011]*. (2011).
31. Paludan-Müller, A., Teindl Laursen, D. R. & Hróbjartsson, A. Mechanisms and direction of allocation bias in randomised clinical trials. *BMC Med. Res. Methodol.* **16**, 133 (2016).
32. Hróbjartsson, A., Kaptchuk, T. J. & Miller, F. G. Placebo effect studies are susceptible to response bias and to other types of biases. *J. Clin. Epidemiol.* **64**, 1223–9 (2011).
33. Benedetti, F. Placebo and the new physiology of the doctor-patient

relationship. *Physiol. Rev.* **93**, 1207–46 (2013).
34. Kessner, S. *et al.* The effect of treatment history on therapeutic outcome: psychological and neurobiological underpinnings. *PLoS One* **9**, e109014 (2014).
35. Gelman, A. & Loken, E. The garden of forking paths: Why multiple comparisons can be a problem, even when there is no “fishing expedition” or “p-hacking” and the research hypothesis was posited ahead of time. *Psychol. Bull.* **140**, 1272–1280 (2014).
36. Schmucker, C. *et al.* Extent of non-publication in cohorts of studies approved by research ethics committees or included in trial registries. *PLoS One* **9**, 1–25 (2014).
37. Petersen, G. L. *et al.* The magnitude of nocebo effects in pain: A meta-analysis. *Pain* **155**, 1426–1434 (2014).
38. Huber, A., Lui, F. & Porro, C. A. Hypnotic susceptibility modulates brain activity related to experimental placebo analgesia. *Pain* **154**, 1509–1518 (2013).
39. Egger, M., Davey Smith, G., Schneider, M. & Minder, C. Bias in meta-analysis detected by a simple, graphical test. *BMJ* **315**, 629–634 (1997).
40. Begg, C. B. & Mazumdar, M. Operating characteristics of a rank correlation test for publication bias. *Biometrics* **50**, 1088–1101 (1994).
41. Zunhammer, M., Bingel, U. & Wager, T. D. Placebo effects on the neurologic pain signature: A meta-analysis of individual participant functional magnetic resonance imaging data. *JAMA Neurol.* 1–10 (2018) doi:10.1001/jamaneurol.2018.2017.

PRISMA 2020 Main Checklist

**TITLE**			
Title	1	Identify the report as a systematic review.	Page 1
**ABSTRACT**			
Abstract	2	See the PRISMA 2020 for Abstracts checklist	
**INTRODUCTION**			
Rationale	3	Describe the rationale for the review in the context of existing knowledge.	Page 5
Objectives	4	Provide an explicit statement of the objective(s) or question(s) the review addresses.	Page 5
**METHODS**			
Eligibility criteria	5	Specify the inclusion and exclusion criteria for the review and how studies were grouped for the syntheses.	Page 20
Information sources	6	Specify all databases, registers, websites, organisations, reference lists and other sources searched or consulted to identify studies. Specify the date when each source was last searched or consulted.	Supplementary Table S3
Search strategy	7	Present the full search strategies for all databases, registers and websites, including any filters and limits used.	Supplement Pages 19-20
Selection process	8	Specify the methods used to decide whether a study met the inclusion criteria of the review, including how many reviewers screened each record and each report retrieved, whether they worked independently, and if applicable, details of automation tools used in the process.	Supplement Page 20, Supplementary Tables S3 and S5

(continued)

Data collection process	9	Specify the methods used to collect data from reports, including how many reviewers collected data from each report, whether they worked independently, any processes for obtaining or confirming data from study investigators, and if applicable, details of automation tools used in the process.	Page 20, Supplement Pages 19-20, Supplementary Table S5
Data items	10a	List and define all outcomes for which data were sought. Specify whether all results that were compatible with each outcome domain in each study were sought (e.g. for all measures, time points, analyses), and if not, the methods used to decide which results to collect.	Pages 21-22
	10b	List and define all other variables for which data were sought (e.g. participant and intervention characteristics, funding sources). Describe any assumptions made about any missing or unclear information.	Pages 21-22, Supplementary Tables S1-S4
Study risk of bias assessment	11	Specify the methods used to assess risk of bias in the included studies, including details of the tool(s) used, how many reviewers assessed each study and whether they worked independently, and if applicable, details of automation tools used in the process.	Page 21, Supplement Pages 20-23
Effect measures	12	Specify for each outcome the effect measure(s) (e.g. risk ratio, mean difference) used in the synthesis or presentation of results.	Pages 22-24
Synthesis methods	13a	Describe the processes used to decide which studies were eligible for each synthesis (e.g. tabulating the study intervention characteristics and comparing against the planned groups for each synthesis (item 5)).	Pages 21-24

(continued)

	13b	Describe any methods required to prepare the data for presentation or synthesis, such as handling of missing summary statistics, or data conversions.	Pages 21-24
	13c	Describe any methods used to tabulate or visually display results of individual studies and syntheses.	Page 24
	13d	Describe any methods used to synthesize results and provide a rationale for the choice(s). If meta-analysis was performed, describe the model(s), method(s) to identify the presence and extent of statistical heterogeneity, and software package(s) used.	Pages 21-24
	13e	Describe any methods used to explore possible causes of heterogeneity among study results (e.g. subgroup analysis, meta-regression).	Supplementary Tables S8-S11
	13f	Describe any sensitivity analyses conducted to assess robustness of the synthesized results.	Supplementary Figure S12
Reporting bias assessment	14	Describe any methods used to assess risk of bias due to missing results in a synthesis (arising from reporting biases).	Page 21, Supplement Pages 20-23
Certainty assessment	15	Describe any methods used to assess certainty (or confidence) in the body of evidence for an outcome.	NA
**RESULTS**			
Study selection	16a	Describe the results of the search and selection process, from the number of records identified in the search to the number of studies included in the review, ideally using a flow diagram.	Supplementary Figure S5, Supplementary Table S3
	16b	Cite studies that might appear to meet the inclusion criteria, but which were excluded, and explain why they were excluded.	Supplementary Table S3
Study characteristics	17	Cite each included study and present its characteristics.	Supplementary Tables S1-4

(continued)

Risk of bias in studies	18	Present assessments of risk of bias for each included study.	Page 6, Supplement Pages 20-23
Results of individual studies	19	For all outcomes, present, for each study: (a) summary statistics for each group (where appropriate) and (b) an effect estimate and its precision (e.g. confidence/credible interval), ideally using structured tables or plots.	Pages 6-13
Results of syntheses	20a	For each synthesis, briefly summarise the characteristics and risk of bias among contributing studies.	Page 6, Supplement Pages 20-23
	20b	Present results of all statistical syntheses conducted. If meta-analysis was done, present for each the summary estimate and its precision (e.g. confidence/credible interval) and measures of statistical heterogeneity. If comparing groups, describe the direction of the effect.	Pages 6-13
	20c	Present results of all investigations of possible causes of heterogeneity among study results.	Supplementary Tables S8-S11
	20d	Present results of all sensitivity analyses conducted to assess the robustness of the synthesized results.	Supplementary Figure S12
Reporting biases	21	Present assessments of risk of bias due to missing results (arising from reporting biases) for each synthesis assessed.	Page 6, Supplement Pages 20-23
Certainty of evidence	22	Present assessments of certainty (or confidence) in the body of evidence for each outcome assessed.	Pages 6-13
**DISCUSSION**			
Discussion	23a	Provide a general interpretation of the results in the context of other evidence.	Pages 14-17
	23b	Discuss any limitations of the evidence included in the review.	Pages 17-18
	23c	Discuss any limitations of the review processes used.	Pages 17-18

(continued)

	23d	Discuss implications of the results for practice, policy, and future research.	Page 19
**OTHER INFORMATION**			
Registration and protocol	24a	Provide registration information for the review, including register name and registration number, or state that the review was not registered.	NA
	24b	Indicate where the review protocol can be accessed, or state that a protocol was not prepared.	NA
	24c	Describe and explain any amendments to information provided at registration or in the protocol.	NA
Support	25	Describe sources of financial or non-financial support for the review, and the role of the funders or sponsors in the review.	Page 29
Competing interests	26	Declare any competing interests of review authors.	Page 30
Availability of data, code and other materials	27	Report which of the following are publicly available and where they can be found: template data collection forms; data extracted from included studies; data used for all analyses; analytic code; any other materials used in the review.	Page 24

From: Page MJ, McKenzie JE, Bossuyt PM, Boutron I, Hoffmann TC, Mulrow CD, et al. The PRISMA 2020 statement: an updated guideline for reporting systematic reviews. MetaArXiv. 2020, September 14. DOI: 10.31222/osf.io/v7gm2. For more information, visit: www.prisma-statement.org
