# Supplementary material for: Meta-analytic evidence for distinct neural correlates of conditioned versus verbally induced placebo analgesia
Source: Nat Commun. 2026 Jul 17;17:6538. doi: 10.1038/s41467-026-74743-0 (PMC13379574; doi:10.1038/s41467-026-74743-0)
Supplement: Supplementary file 2 — Reporting Summary [file 41467_2026_74743_MOESM2_ESM.pdf]

nature portfolio

Corresponding author(s): Tamas Spisak

Last updated by author(s): Mar 4, 2026

Reporting Summary

Nature Portfolio wishes to improve the reproducibility of the work that we publish. This form provides structure for consistency and transparency in reporting. For further information on Nature Portfolio policies, see our Editorial Policies and the Editorial Policy Checklist.

Statistics

For all statistical analyses, confirm that the following items are present in the figure legend, table legend, main text, or Methods section.

n/a	Confirmed	
☐	☒	The exact sample size (*n*) for each experimental group/condition, given as a discrete number and unit of measurement
☐	☒	A statement on whether measurements were taken from distinct samples or whether the same sample was measured repeatedly
☐	☒	The statistical test(s) used AND whether they are one- or two-sided<br>*Only common tests should be described solely by name; describe more complex techniques in the Methods section.*
☐	☒	A description of all covariates tested
☐	☒	A description of any assumptions or corrections, such as tests of normality and adjustment for multiple comparisons
☐	☒	A full description of the statistical parameters including central tendency (e.g. means) or other basic estimates (e.g. regression coefficient) AND variation (e.g. standard deviation) or associated estimates of uncertainty (e.g. confidence intervals)
☐	☒	For null hypothesis testing, the test statistic (e.g. *F*, *t*, *r*) with confidence intervals, effect sizes, degrees of freedom and *P* value noted<br>*Give P values as exact values whenever suitable.*
☒	☐	For Bayesian analysis, information on the choice of priors and Markov chain Monte Carlo settings
☐	☒	For hierarchical and complex designs, identification of the appropriate level for tests and full reporting of outcomes
☐	☒	Estimates of effect sizes (e.g. Cohen's *d*, Pearson's *r*), indicating how they were calculated

Our web collection on statistics for biologists contains articles on many of the points above.

Software and code

Policy information about availability of computer code

Data collection	The present study is a systematic meta-analysis of individual participant data across k = 16 peer-reviewed, within-participant placebo neuroimaging studies. No specific software was used for data collection.
Data analysis	Analysis code for carrying out data cleaning is available at: https://github.com/mzunhammer/PlaceboImagingMetaAnalysis. The complete code for reproducing the analyses and figures presented in the manuscript are available at: https://github.com/pni-lab/placebo-conditioning-meta-analysis.

For manuscripts utilizing custom algorithms or software that are central to the research but not yet described in published literature, software must be made available to editors and reviewers. We strongly encourage code deposition in a community repository (e.g. GitHub). See the Nature Portfolio guidelines for submitting code & software for further information.

Data

Policy information about availability of data

All manuscripts must include a data availability statement. This statement should provide the following information, where applicable:

- Accession codes, unique identifiers, or web links for publicly available datasets
- A description of any restrictions on data availability
- For clinical datasets or third party data, please ensure that the statement adheres to our policy

The derivative/summary data generated in this study are publicly available on GitHub (https://github.com/pni-lab/placebo-conditioning-meta-analysis; DOI: 10.5281/zenodo.18837986). Individual-level raw data included in this meta-analysis are available under restricted access due to legal and ethical constraints linked

to the original studies (including participant-consent and ethics-approval limits). Access can be requested by contacting ulrike.bingel@uk-essen.de. Data may be shared for approved scientific/reproducibility purposes under a data-use agreement and for a defined access period.

Research involving human participants, their data, or biological material

Policy information about studies with human participants or human data. See also policy information about sex, gender (identity/presentation), and sexual orientation and race, ethnicity and racism.

Reporting on sex and gender	We report on the % of male participants in each single study, and included sex as a covariate in all analyses.
Reporting on race, ethnicity, or other socially relevant groupings	We included age as a covariate in all analyses.
Population characteristics	All relevant characteristics of each individual sample in the meta-analysis have been extracted and used as moderator in the present meta-analysis.
Recruitment	No recruitment was part of the study.
Ethics oversight	The present research complies with all relevant ethical regulations, including written consent, as all individual studies obtained ethical approval from their respective institutions.

Note that full information on the approval of the study protocol must also be provided in the manuscript.

Field-specific reporting

Please select the one below that is the best fit for your research. If you are not sure, read the appropriate sections before making your selection.

☒ Life sciences ☐ Behavioural & social sciences ☐ Ecological, evolutionary & environmental sciences

For a reference copy of the document with all sections, see nature.com/documents/nr-reporting-summary-flat.pdf

Life sciences study design

All studies must disclose on these points even when the disclosure is negative.

Sample size	Our analysis included single-participant data from a total of n = 415 participants. Since this is a meta-analysis of individual participant data the sample size was not determined a-priori but based on the available/shared data.
Data exclusions	Our data acquisition procedure identified 96 published articles, of which 28 were selected as eligible. In the present analysis, we excluded studies with a between-participant design, leading to a final dataset consisting of k = 16 studies and n = 415 individuals (n = 409 after excluding participants with missing pain ratings).
Replication	The complete code for reproducing the analyses and figures presented in the manuscript are available at: https://github.com/pni-lab/placebo-conditioning-meta-analysis.
Randomization	The meta-analysis included only within-subject studies, and thus no randomized group allocation. However, placebo vs. control conditions were randomly assigned. Problems due to non-random allocation are considered minor in within-subject designs as all participants undergo both treatments.
Blinding	Meta-analysts were not blinded to the group (placebo vs control) labels as it was deemed difficult/futile. Summary results for all included studies were already published. The analysts involved were intimately familiar with the results of these published studies. In many cases one look at study-level summary images would have unblinded analysts.

Reporting for specific materials, systems and methods

We require information from authors about some types of materials, experimental systems and methods used in many studies. Here, indicate whether each material, system or method listed is relevant to your study. If you are not sure if a list item applies to your research, read the appropriate section before selecting a response.

Materials & experimental systems

n/a	Involved in the study
☒	☐ Antibodies
☒	☐ Eukaryotic cell lines
☒	☐ Palaeontology and archaeology
☒	☐ Animals and other organisms
☒	☐ Clinical data
☒	☐ Dual use research of concern
☒	☐ Plants

Methods

n/a	Involved in the study
☒	☐ ChIP-seq
☒	☐ Flow cytometry
☐	☒ MRI-based neuroimaging

Plants

Seed stocks	NA
Novel plant genotypes	NA
Authentication	NA

Magnetic resonance imaging

Experimental design

Design type	Participant-level meta-analysis based on functional neuroimaging experiments, mix of block- and event-related designs. Each individual study included a task-based within-subject design.
Design specifications	Different for each individual study, see Zunhammer et al (2021) Table 1/Supplementary Tables 2-7, and Supplementary Tables 1-4 in this work.
Behavioral performance measures	Pain ratings in placebo and control conditions, which are subtracted to calculate a difference score (the placebo effect).

Acquisition

Imaging type(s)	Functional imaging
Field strength	4x 1.5T, 12x 3T
Sequence & imaging parameters	Various, see Zunhammer et al (2021) Supplementary Table 4
Area of acquisition	Whole brain

Diffusion MRI ☐ Used ☒ Not used

Preprocessing

Preprocessing software	Various, see Zunhammer et al (2021) Supplementary Table 5
Normalization	Various, see Zunhammer et al (2021) Supplementary Table 5
Normalization template	Various, see Zunhammer et al (2021) Supplementary Table 5
Noise and artifact removal	Various, see Zunhammer et al (2021) Supplementary Table 5
Volume censoring	Various, see Zunhammer et al (2021) Supplementary Table 5

Statistical modeling & inference

Model type and settings	Voxel-wise mediation analysis, corrected for age, sex and control pain rating, and modeling studies with dummy regressors orthogonalized to induction type. The mediation analysis was implemented using ordinary least squares linear models with a custom analysis code written in Python (see section on Code Availability). P-values were derived from sign-flipped null modeling.

Effect(s) tested

Path A (induction type effect on brain response), Path B (brain response effect on behavioral placebo analgesia), Path C (induction type effect on behavioral placebo analgesia) mediation effect (A*B), pooled placebo brain response, INST & COND +INST conjunction. Supplementary analyses: age, stimulation laterality, sex.

Specify type of analysis: ☒ Whole brain ☐ ROI-based ☐ Both

Statistic type for inference

(See Eklund et al. 2016)

Voxel-wise beta coefficients, mass-univariate mediation analysis, permutation (sign flipping)-based null modeling.

Correction

False discovery rate, tail approximation via Generalized Pareto distribution.

Models & analysis

n/a	Involved in the study
☒	☐ Functional and/or effective connectivity
☒	☐ Graph analysis
☐	☒ Multivariate modeling or predictive analysis

Multivariate modeling and predictive analysis

We applied two multivariate signatures: the NPS and the SIIPS. No new predictive models were trained in the present study.
