# Supplementary material for: Meta-analytic evidence for distinct neural correlates of conditioned versus verbally induced placebo analgesia
Source: Nat Commun. 2026 Jul 17;17:6538. doi: 10.1038/s41467-026-74743-0 (PMC13379574; doi:10.1038/s41467-026-74743-0)
Supplement: Supplementary file 3 — Transparent Peer Review file [file 41467_2026_74743_MOESM3_ESM.pdf]

nature portfolio

Peer Review File

Meta-analytic evidence for distinct neural correlates of conditioned versus verbally induced placebo analgesia

Corresponding Author: Dr Tamas Spisak

This file contains all reviewer reports in order by version, followed by all author rebuttals in order by version.

Version 0:

Reviewer comments:

Reviewer #1

(Remarks to the Author)
The manuscript by Spisak et al. presents noteworthy results from a meta-analysis across 16 studies involving placebo conditioning responses to painful stimuli. From the large sample size of over 400 data sets of behavioral response and fMRI data, the authors effectively compared 2 placebo paradigms of instruction alone vs. instruction plus conditioning. They confirmed greater behavioral efficacy of the instruction plus conditioning placebo and identified unique contributors to the 2 placebo conditions, providing novel and in-depth insight of brain mechanisms of placebo response. The work supports established literature and builds significantly on what has been previously shown in prior placebo studies. The conclusions are supported, and the methodology appears to be sound, with enough detail and provided code and images to reproduce the work. A few suggestions for revisions are listed below:
- In the abstract lines 99-100, the statement about the NIPS is somewhat unclear. It would be helpful to adjust this statement slightly and to add a bit more context in the discussion (lines 520-521), in particular, to add comments on why the brain's placebo profile is less similar to the SIIPS (in contrast to it being more related to NIPS).
- Figure 3's legend reference does not seem to match the brain regions presented since somatosensory shows "higher" response but it is listed after "increased" with the other regions. Also, in this legend, there seems to be missing the Table reference after "See ____ for a comprehensive list" maybe because it was split there to insert the table.
- The habenula ROI analysis seems to be somewhat disjointed within the manuscript. While it is presented as one of the listed aims and the results are adequately described, the rationale for including the habenula ROI is missing from the introduction/first section of the results. And then the habenula results are not described much in the discussion. Since the habenula results seem to be minimal in terms of explaining the placebo response and mediation analysis relationships, the authors might consider moving it to the supplement (and still include some additional statements to explain why they investigated the habenula ROI).
- In the figures with the horizontal brain images (slices), left/right needs to be indicated.
- Typo on page 25 line 629 "as" makes the statement a fragment, rather than a sentence.
- While the analyses were adjusted for sex (in addition to age and other factors), there needs to be at least some discussion of whether there were detected any sex differences across the studies. Additionally, participants' sex distribution among the studies would be helpful to include in the supplement.

(Remarks on code availability)
I glanced at the code and it is provided at the listed github link. The documents on the github look like they are organized and thorough, so I would expect the code to be organized and work as well.
I did not evaluate whether the code could be installed or run.

Reviewer #2

(Remarks to the Author)
Spisak and colleagues present a meta-analytic approach regarding human brain activity associated with conditioning vs expectancy modulated placebo analgesia. They pose an interesting question at the outset – whether our field suffers a bias to misinterpret PA elicited from combining expectancy modulations + conditioning or conditioning effects alone, and whether associated brain activity when PA is elicited through these two approaches is separable. Across a large dataset pooled from 16 within-subjects designs, they answer this question through main effects, mediation, and regression analyses to weave a

story displaying the likely neural contributors to the differences in behavioural PA observed when expectations alone are utilized versus when combining conditioning effects with expectancy modulations elicit a stronger PA. Their methodology and sample are sound, and the approach is reproducible, using standardized pattern masks for the majority of their findings. I make predominantly minor comments with only few suggestions for further explanation / analysis should the authors also believe these suggestions would strengthen the manuscript with inclusion.

Thank you for the opportunity to review this work, and I would be willing to provide further feedback on resubmission if required.

All the best for the author's future work,

Major Comments:
- Page 7: It is stated age, sex, and pain ratings in control condition were added as covariates – however in table 1 (page 6) both Kong 2009 and Zeidan 2015 are marked to only include placebo-treatment groups, how were participants from these two studies pain ratings covariates loaded into the mediation analysis? I may be misunderstanding what is meant by symbol "a" in this table, as the listed inclusion criteria is set that matched placebo and control conditions must be present above.
- The primary sensory cortex and thalamic changes in both paths "A" and "B" are of interest, especially seeing past observations of little to no changes in ascending nociceptive pathways related to PA – can the authors comment on the laterality effects in S1 and thalamus? That is, are there equal number of studies included where pain was applied to the left or right sides of the body so that these changes reflect both side "contralateral" changes – if the authors were to flip the contrast images for all studies where pain was only applied to the right body side for instance so that all contrast images simulated "left body side noxious stimulus application" do they think they would only observe right hemisphere S1 / thalamic changes? I think this could be an interesting addition to complement whether these changes are necessarily solely responding to ascending input or rather whether the bilateral changes in S1 are just part of the cortical architecture involved in COND+INST PA responses relative to control.
- Limitations: average age notwithstanding a weighting for sample size between studies is 26.5 years, given evidence of changes in brain structure and function of several of the sites listed as mediators across the lifespan and with aging / during the presence of certain cognitive disease-states, perhaps the authors could include this as a limitation / suggest as a future direction an expansion of this work to different age groups to ensure these mediators retain their relative contributions across the lifespan.
- An entire analysis (CA3) is devoted to investigating connectivity changes with Habenula but these results for INST / partial null findings in COND+INST are seemingly not discussed within the discussion – could the authors expand on their findings in this section about CA3 / include their thoughts on why this approach yielded an opposite effect than what would be expected given the other imaging findings – that is, greater deactivations within INST alone than with INST+COND?

Minor Comments:
- Suggested change in abstract 4th last line from: "yielded greater analgesia" to something which clearly defines this is a subjective change in perceived pain and not pharmacologically-driven analgesia – this is personal opinion, but the authors do use "subjective pain relief" in the introduction.
- Page 19. This may be outside the scope of this manuscript, but since the authors suggest Bayesian level encoding as partly responsible for the altered activity in pain-responsive regions such as dpIns and S1/2 – do the authors have access to the VAS ratings provided by participants during the conditioning process? Do they expect or can they measure statistically whether the average VAS difference between control- and placebo-treated sites during conditioning relates to the magnitude of either deactivation within these sites or across the NPS?
- Page 20 – typo "…two regions that were also found to mediate the effects of induction method on placebo analgesia our study".
- Page 21 - If word count allows, could the authors expand their comment on VMPFC associations with chronic pain – it is interesting that the reference used explores the transitionary stages into chronic pain, alongside the increasing suggestion that components of chronic pain symptomatology are driven from encoded, learned behaviour – this line of arguments could be interesting to explore and dovetail nicely with the belief-updating role of the VMPFC proposed here.
- Page 21 – typo "may compliments"
- Page 25 – I think this is a typo? "costume analysis code" – should this be "custom"?

(Remarks on code availability)
I have reviewed the code provided on Github, all seems to be present notwithstanding the 1st level contrast images which are noted to be available on request to the placebo imaging consortium. A readme is present, as well as basic installation instructions for use within python. Functions are provided within the manuscript itself and the preprint with extensive instructions for reproducibility.

Reviewer #3

(Remarks to the Author)
This paper by Spisak et al. provides a meta-analysis of individual neuroimaging data, revealing distinct neural mechanisms for placebo analgesia induced by suggestions alone versus suggestions combined with conditioning.

While this study addresses an important question, the overall sample size in mega-analysis is small for functional magnetic resonance imaging (fMRI) data, and it is constrained by some significant methodological and interpretive limitations, such as including study heterogeneity, unverified analytical assumptions, and several unaddressed confounding factors. These

concerns may compromise the robustness and clarity of the findings. The following are several major and minor concerns.

Major concerns

(1) The meta-analysis may be subject to publication bias, as studies with significant placebo effects are more likely to be published. This could inflate the overall effect estimates.

(2) The conditioning protocols varied considerably across studies in terms of duration, intensity, and reinforcement procedures. This heterogeneity may have influenced the results and the authors can clarify whether it was accounted for in the analysis or interpretation.

(3) Multiple claims of stronger associations are based on p-values instead of coefficients. For example, the Figure 6 caption.

(4) Selection bias is not addressed in the manuscript. Since the authors collected data by contacting investigators from prior studies, it would be helpful to report additional details, such as the number of studies excluded due to unavailable data and the number of authors contacted versus those who responded, to assess the risk of inclusion bias.

(5) The conjunction analysis is appropriate for identifying core regions commonly engaged across both induction methods, but its statistical conservativeness may overlook meaningful activation patterns present in only one condition or marginally in both. The authors may consider complementary approaches (e.g., inclusive masking or direct contrasts) to provide a fuller picture.

(6) The sample sizes across included studies vary substantially, from as few as n=10 to over n=40. It is unclear whether sample size weighting or sensitivity analyses were applied to reduce potential bias from small-sample studies in group-level statistics (e.g., conjunction analysis, mediation). Without such adjustments, small studies could exert disproportionate influence on the results. In addition, the sample size is small for fMRI studies, and it has been demonstrated that replication of fMRI findings may need much larger sample size.

(7) The studies included diverse pain stimuli (such as heat, laser, and distension), which likely engage different neural pathways and may influence placebo responses. The authors should consider its potential as a confounding factor.

(8) In Figure 4, why in caudate, the correlation seems to be positive (red), but the Pearson correlation r is negative?

(9) The assumptions underlying the mediation analysis, such as the absence of unmeasured confounding between the mediator and the outcome, are not discussed or evaluated. It would strengthen the study to explicitly address these assumptions and their potential impact on interpretation.

Minor Concerns

(1) The methods section does not clearly specify the statistical software or packages used for key analyses such as the mixed-effects model (CA1) or Levene's test. Additionally, it is unclear which estimation method was used to compute effect sizes (e.g., REML vs. ML) in the mixed model.

(2) Although the 3-point difference in VAS ratings between COND+INST and INST is statistically significant, prior research suggests that a minimal clinically important difference (MCID) is typically around 10–15 points on a 100-point scale. The authors should distinguish between statistical and clinical significance and acknowledge this limitation in the discussion.

(3) Line 276-277, (Figure 3B,). Need to clean up.

(4) Correct the citation format. "A detailed description of the data collection procedures can be found in 14" (Line 603)

(Remarks on code availability)

Reviewer #4

(Remarks to the Author)
I co-reviewed this manuscript with one of the reviewers who provided the listed reports. This is part of the Nature Communications initiative to facilitate training in peer review and to provide appropriate recognition for Early Career Researchers who co-review manuscripts.

(Remarks on code availability)

Version 1:

Reviewer comments:

Reviewer #1

(Remarks to the Author)
Through the revisions of the manuscript, all of my and other reviewers' comments appear to be addressed very well.

(Remarks on code availability)

Reviewer #2

(Remarks to the Author)
The authors have resolved all of my comments with specific analyses and discussion elements included which do not require further adjustments in my opinion.
The reordering of habenula analysis to supplement also improves manuscript structure and flow.
Compliments to the authors for this work - which is a lovely addition to our field
Dr Lewis Crawford

(Remarks on code availability)
Code assessed in first revision cycle. No outstanding issues

Reviewer #3

(Remarks to the Author)
I appreciate the authors' thorough revisions, which have addressed most of my concerns.

I recognize that this study likely represents the largest dataset assembled to date for this type of placebo neuroimaging meta-analysis. That said, given the substantial heterogeneity across the included studies, it remains unclear how this variability may affect effective power and the robustness of the pooled estimates.

To help readers interpret the strength of the evidence, it would be valuable to add a formal power/sensitivity analysis (e.g., minimal detectable effect sizes) demonstrating what effect sizes are detectable with the current sample size under plausible assumptions for a within-participant, task-based fMRI design, and clarifying any remaining risks to power and inference.

(Remarks on code availability)

Reviewer #4

(Remarks to the Author)
I co-reviewed this manuscript with one of the reviewers who provided the listed reports. This is part of the Nature Communications initiative to facilitate training in peer review and to provide appropriate recognition for Early Career Researchers who co-review manuscripts.

(Remarks on code availability)

Open Access This Peer Review File is licensed under a Creative Commons Attribution 4.0 International License, which permits use, sharing, adaptation, distribution and reproduction in any medium or format, as long as you give appropriate credit to the original author(s) and the source, provide a link to the Creative Commons license, and indicate if changes were made.
In cases where reviewers are anonymous, credit should be given to 'Anonymous Referee' and the source.
The images or other third party material in this Peer Review File are included in the article's Creative Commons license, unless indicated otherwise in a credit line to the material. If material is not included in the article's Creative Commons license and your intended use is not permitted by statutory regulation or exceeds the permitted use, you will need to obtain permission directly from the copyright holder.
To view a copy of this license, visit https://creativecommons.org/licenses/by/4.0/

Reviewer #1 (Remarks to the Author)

The manuscript by Spisak et al. presents noteworthy results from a meta-analysis across 16 studies involving placebo conditioning responses to painful stimuli. From the large sample size of over 400 data sets of behavioral response and fMRI data, the authors effectively compared 2 placebo paradigms of instruction alone vs. instruction plus conditioning. They confirmed greater behavioral efficacy of the instruction plus conditioning placebo and identified unique contributors to the 2 placebo conditions, providing novel and in-depth insight of brain mechanisms of placebo response. The work supports established literature and builds significantly on what has been previously shown in prior placebo studies. The conclusions are supported, and the methodology appears to be sound, with enough detail and provided code and images to reproduce the work.

We thank the reviewer for their positive evaluation of our work and for their constructive feedback. We reply to each of their other comments below and cite all changes made to the manuscript (reviewer comments in blue, author replies in black, manuscript text in grey italics and new additions in green italics). Page and line numbers refer to the revised manuscript with tracked changes enabled.

A few suggestions for revisions are listed below:
- In the abstract lines 99-100, the statement about the NIPS is somewhat unclear. It would be helpful to adjust this statement slightly and to add a bit more context in the discussion (lines 520-521), in particular, to add comments on why the brain's placebo profile is less similar to the SIIPS (in contrast to it being more related to NIPS).

We thank the reviewer for this suggestion. The confusion stems from a typo in the original text:

Page 23, Lines 557-560: *"Although there was no significant voxel-level interaction between brain-behavior correlation and induction type, we observed that the NPS, a weighted multivariate signature~~ of pain~~ linked to nociception, predicted behavioral analgesia in the COND+INST condition more accurately than ~~the stimulus intensity-independent pain signature (SIIPS; 13)~~ in the INST only condition."*

We apologize for this oversight. In the revised text we clarify that the similarity of the brain map encoding placebo effects in the NPS is not significantly different from that of the SIIPS:

Page 19, Lines 436-444: "*Pooled over induction types, both the NPS and the SIIPS exhibited a statistically significant negative correlation with behavioral analgesia (NPS: T(390)=- 407 3.169, r=.159, p=.0015, two-tailed; SIIPS: (T(390)=-3.109, r=.155, p=.0031, two-tailed), with no significant difference between them. ~~Pooled over induction types, the NPS exhibited a statistically significant negative correlation with behavioral analgesia (T(390)=-3.169, r=.159, p=.0015, two-tailed).~~ When contrasting induction types, we observed a stronger NPS-analgesia association with COND+INST (T(252)=-3.025, r = .19, p =.002, two-tailed) than with INST only, (T(136)=-1.184, r = .1, p = .12, two-tailed). The difference between induction types was statistically significant (interaction: T(409) = -1.83, p = .034, two-tailed). Detailed results are*

presented in Table 7. The SIIPS showed ~~a weak but significant negative correlation with behavioral analgesia both when data were pooled across the induction types (T(390)=-3.109, r=0.155, p=0.0031, two-tailed) and when analyzed separately for each (r = .15 and .16, p = .05 and .02 for INST and COND+INST, respectively), with~~ no statistically significant interaction with induction type."

We have also revised the abstract to improve clarity:

Page 3, Lines 99-100: *"Conditioning also strengthened the negative association between analgesia and nociceptive activity, as ~~reflected in~~ quantified by the Neurologic Pain Signature."*

- Figure 3's legend reference does not seem to match the brain regions presented since somatosensory shows "higher" response but it is listed after "increased" with the other regions. Also, in this legend, there seems to be missing the Table reference after "See _____ for a comprehensive list" maybe because it was split there to insert the table.

We thank the reviewer for spotting this discrepancy. In the revised manuscript, the Figure 3 caption now reads as follows:

Page 13, Lines 345-347: *"(B) COND+INST resulted in significantly higher placebo-related activation in the middle temporal gyrus, DMPFC, DLPFC, dorsal inferior parietal cortex and decreased activation in middle insular and somatosensory areas."*

We have also corrected a Word formatting glitch that incorrectly referenced the entire Table 4 within the caption of Figure 3 instead of its label.

- The habenula ROI analysis seems to be somewhat disjointed within the manuscript. While it is presented as one of the listed aims and the results are adequately described, the rationale for including the habenula ROI is missing from the introduction/first section of the results. And then the habenula results are not described much in the discussion. Since the habenula results seem to be minimal in terms of explaining the placebo response and mediation analysis relationships, the authors might consider moving it to the supplement (and still include some additional statements to explain why they investigated the habenula ROI).

We agree that the habenula ROI analysis was not well integrated into the main narrative. In line with this comment, and a similar point raised by Reviewer #2, we have moved the analysis (formerly CA3) and its description to the supplementary materials (Supplementary Table S7), where we have also included the rationale for investigating the habenula to provide full context.

Supplement, Page 12, Lines 124-137: *"The habenula has previously also been implicated in pain modulation and placebo mechanisms [2,3]. We obtained the habenula ROI from the subcortical brain atlas of [4] and divided it into bilateral components (by x = 0). Within all voxels of each ROI, we averaged the voxel-wise path a, path b and mediation (a*b) regression coefficients, as well as the group-mean estimates for INST and COND+INST (see Mediation Analysis in the main manuscript). To construct the null distribution for statistical inference, we used the ROI-wide averages of the voxel-*

*wise null-distribution estimates, calculated based on sign-flipped 10 000 surrogate datasets, separately for path a, b, a*b and the INST and COND+INST group means. Similarly to the voxel-wise analysis, we calculated p-values by fitting a generalized Pareto distribution on the tail of the null distributions. We found that activity in the left habenula showed a significant placebo-related decrease with INST (harmonized effect size difference: -5.1%, p = .016) and COND+INST (-5.3%, p = .046), replicating our previous findings [2]. No statistically significant effects were observed in the right habenula. The ROI analysis also found no significant path a, path b, or mediation effects in either the left or right habenula."*

- In the figures with the horizontal brain images (slices), left/right needs to be indicated.

We have now added this information.

- Typo on page 25 line 629 "as" makes the statement a fragment, rather than a sentence.

We have revised the sentence as follows:

Page 28, Line 736-737: *"Additionally, we obtained study-wise standardized effect size summaries of the behavioral PA by calculating Hedges' g scores~~. Hedges' g,~~ as is common in meta-analyses."*

- While the analyses were adjusted for sex (in addition to age and other factors), there needs to be at least some discussion of whether there were detected any sex differences across the studies. Additionally, participants' sex distribution among the studies would be helpful to include in the supplement.

We thank the reviewer for highlighting the importance of discussing sex differences. Analysis of the behavioral data revealed no significant sex differences (Table 2; β = 2.247, T = 1.17, p = .243). Regarding brain responses, while our primary models already included sex as a covariate, we agree that a direct investigation of sex differences provides valuable additional insight. We have therefore conducted a dedicated analysis comparing placebo-related brain responses between male and female participants, controlling for age, induction type, and control pain ratings. We discuss these results in the revised manuscript as follows:

Page 12, Lines 320-330: *"While the primary focus of the present manuscript is the effect of induction type, we also conducted a supplementary analysis examining the effect of sex and age on placebo-related brain responses. We found that females, compared with males, showed stronger placebo-related deactivations in key pain-processing regions such as the anterior insula, ACC, and S1 (see Supplementary Figure S7 and Supplementary Table S8 for details). These findings align with previous literature reporting sex-based differences in pain perception and modulation and underscore the importance of considering sex as a biological variable in pain and placebo research [5,6]. Furthermore, we identified five small clusters showing a significant age effect (all showing higher activation with increasing age, see Supplementary Figure S8 and Supplementary Table S9). While a full mechanistic explanation of these results is beyond the scope of this meta-analysis, they highlight an important avenue for future investigation."*

To present these new results, we have now added a new figure and corresponding table to the Supplement (Supplementary Figure S7 and Table S9). The sex distribution across studies is reported in Table 1. We have added an additional supplementary figure (Supplementary Figure S4) to visualize the distribution. We present all new figures below for convenience.

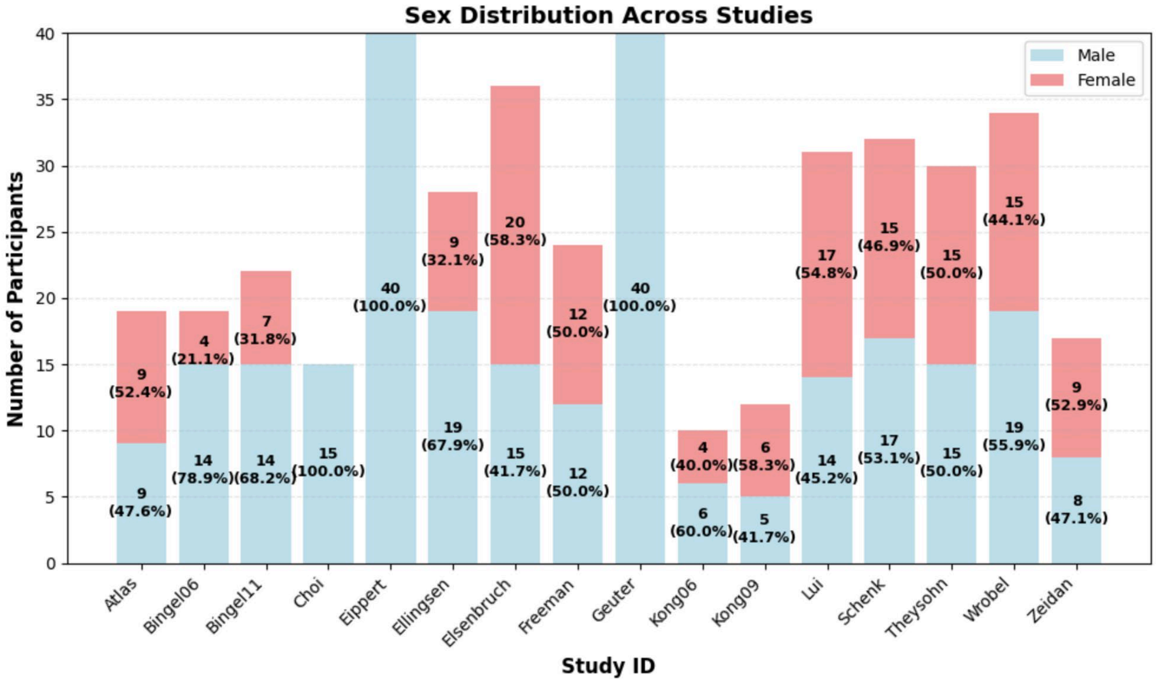

Supplementary Figure S4. *Sex distribution across the studies.*

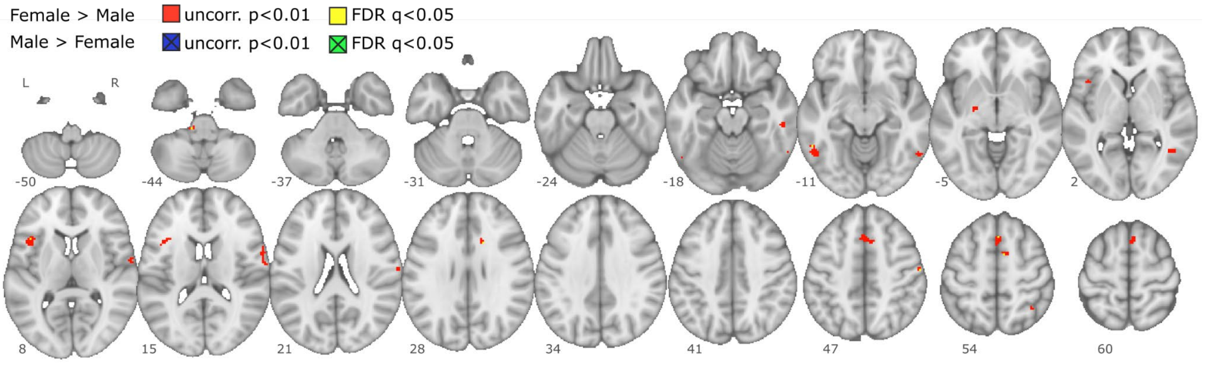

Supplementary Figure S7. *Sex distribution across studies. Sex differences in placebo analgesia. We found multiple clusters where placebo-related brain activity was significantly higher in females (FDR q < 0.05), including regions like the anterior insula, DMPFC, ACC, S1 and basal ganglia. In the case of uncorrected results (p < .01, red), we only show clusters that contain at least one voxel surviving false discovery rate correction (q < 0.05). No voxels showed increased activity in males after FDR-correction. See* ***Supplementary Table S7*** *for a comprehensive list of activations significant after FDR correction.*

Reviewer #1 (Remarks on code availability)

I glanced at the code and it is provided at the listed github link. The documents on the github look like they are organized and thorough, so I would expect the code to be organized and work as well.

I did not evaluate whether the code could be installed or run.

We thank the reviewer for checking the code repository and for their positive feedback on its organization, which we designed to be both reproducible and adaptable for other researchers.

Reviewer #2 (Remarks to the Author)

Spisak and colleagues present a meta-analytic approach regarding human brain activity associated with conditioning vs expectancy modulated placebo analgesia. They pose an interesting question at the outset – whether our field suffers a bias to misinterpret PA elicited from combining expectancy modulations + conditioning or conditioning effects alone, and whether associated brain activity when PA is elicited through these two approaches is separable. Across a large dataset pooled from 16 within-subjects designs, they answer this question through main effects, mediation, and regression analyses to weave a story displaying the likely neural contributors to the differences in behavioral PA observed when expectations alone are utilized versus when combining conditioning effects with expectancy modulations elicit a stronger PA. Their methodology and sample are sound, and the approach is reproducible, using standardized pattern masks for the majority of their findings. I make predominantly minor comments with only few suggestions for further explanation / analysis should the authors also believe these suggestions would strengthen the manuscript with inclusion.

Thank you for the opportunity to review this work, and I would be willing to provide further feedback on resubmission if required.
All the best for the author's future work,

We sincerely thank the reviewer for their thoughtful and positive assessment of our manuscript. We appreciate the constructive suggestions provided, have addressed them point-by-point below, and cite all changes made to the manuscript (reviewer comments in blue, author replies in black, manuscript text in grey italics and new additions in green italics). Page and line numbers refer to the revised manuscript with tracked changes enabled.

Major Comments:
- Page 7: It is stated age, sex, and pain ratings in control condition were added as covariates – however in table 1 (page 6) both Kong 2009 and Zeidan 2015 are marked to only include placebo-treatment groups, how were participants from these two studies pain ratings covariates loaded into the mediation analysis? I may be misunderstanding what is meant by symbol "a" in this table, as the listed inclusion criteria is set that matched placebo and control conditions must be present above.

We thank the reviewer for requesting this important clarification. The footnote in Table 1 was indeed ambiguous. To clarify, all studies included in our meta-analysis employed a within-participant design. The two studies in question (Kong et al., 2009; Zeidan et al., 2015) were multi-arm trials, and we exclusively used data from their placebo-treatment arms, each of which contained the required within-participant (placebo vs. control) component. Therefore, matched control pain ratings were available for all participants.

We have revised the footnote in Table 1 to state this explicitly:

Page 6, Line 185, note to Table 1: *"a Only placebo-treatment groups"* => *"[a] Multi-arm trial; only the placebo arm was retained, which included within-person placebo and control conditions."*

- The primary sensory cortex and thalamic changes in both paths "A" and "B" are of interest, especially seeing past observations of little to no changes in ascending nociceptive pathways related to PA – can the authors comment on the laterality effects in S1 and thalamus? That is, are there equal number of studies included where pain was applied to the left or right sides of the body so that these changes reflect both side "contralateral" changes – if the authors were to flip the contrast images for all studies where pain was only applied to the right body side for instance so that all contrast images simulated "left body side noxious stimulus application" do they think they would only observe right hemisphere S1 / thalamic changes? I think this could be an interesting addition to complement whether these changes are necessarily solely responding to ascending input or rather whether the bilateral changes in S1 are just part of the cortical architecture involved in COND+INST PA responses relative to control.

We thank the reviewer for this insightful question regarding the laterality of our findings. We agree that variable stimulation side (and location) may introduce biases in regions with strong lateralization, such as the primary sensory cortices and the thalamus. While flipping the images could provide an elegant solution, we believe our dataset is unfortunately not well suited for such analysis due to (i) the limited accuracy of matching our data to a symmetric template without having access to the individual T1 and native space functional maps (as we only collected standard space activation maps), (ii) the lack of clear laterality in rectal distension studies (Elsenbruch, Theyson) and in studies using one arm as a control site and the other as placebo (Bingel06); and (iii) potential interpretational issues given prior evidence for lateralized pain processing independent of stimulus side [7,8].

Instead, we performed a new supplementary analysis to directly quantify stimulation side effects on placebo brain responses:

Page 12, Lines 331-339: *"Furthermore, as the laterality of pain stimulation varied considerably across the involved studies (Supplementary Figure S2), we conducted a supplementary analysis directly comparing placebo brain responses with left-sided versus right-sided stimulation, adjusted for age, sex and control pain ratings. Studies with bilateral stimulation (Bingel06) or without clear laterality (Elsenbruch, Theyson) were excluded, resulting in 13 studies with a total of 298 participants. This analysis revealed significant stimulation-side-dependent differences in activity mainly in the sensory and motor cortices, but also in parietal and frontal regions and the amygdala (Supplementary Figure S9, Supplementary Table S10). The observed lateralization patterns were consistent with the well-documented hemispheric specialization in sensory/nociceptive function."*

We have added a new paragraph into the discussion discussing related limitations:

Page 25, Lines 634-639: *"A further limitation is that the laterality of pain stimulation varied considerably across the included studies (Supplementary Figure S2). In our supplementary analysis examining the effects of stimulation side on placebo brain*

responses, we did not find significant differences in regions that exhibited significant mediation effects in our main analysis (including the VMPFC, caudate, S2, insula, or cerebellar vermis), suggesting that the findings of our mediation analysis are unlikely to be strongly influenced by laterality."

For convenience, we present Supplementary Figure S9 detailing the results below.

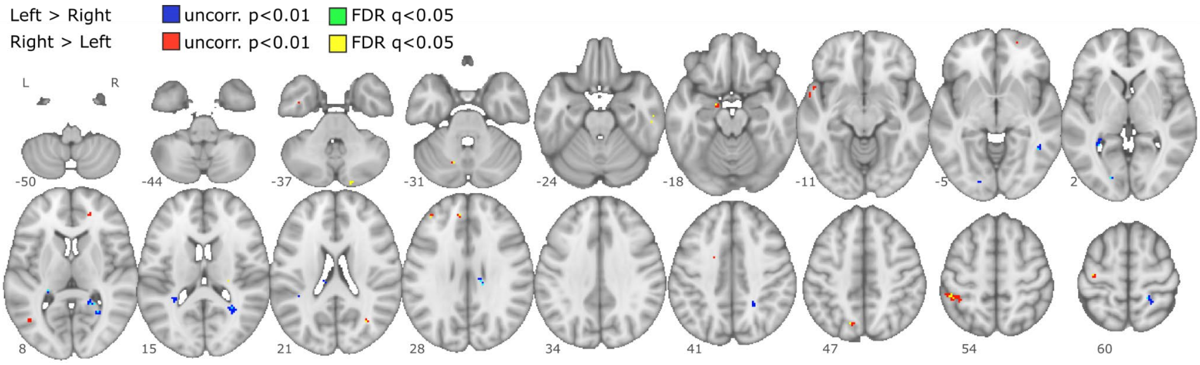

Supplementary Figure S9. *Differences in placebo analgesia in relation to stimulation laterality, adjusted for age, sex and control pain ratings. In this analysis, we excluded studies with bilateral stimulation (Bingel06) or without clear laterality (Elsenbruch, Theyson), resulting in 13 studies with a total of 298 participants. We found multiple clusters where placebo-related brain activity was significantly related to stimulation side (FDR q < 0.05), including mostly sensory and motor areas, but also parietal and frontal regions and the amygdala. The lateralization of differences generally reflected the well documented hemispheric specialization in sensory/nociceptive function. In the case of uncorrected results (p < .01, red and blue), we only show clusters that contain at least one voxel surviving false discovery rate correction (q < 0.05). Images are shown in neurological convention (left is left). Standard-space Z-coordinates are shown for each slice. See* ***Supplementary Table S5*** *for a comprehensive list of activations significant after FDR correction.*

- Limitations: average age notwithstanding a weighting for sample size between studies is 26.5 years, given evidence of changes in brain structure and function of several of the sites listed as mediators across the lifespan and with aging / during the presence of certain cognitive disease-states, perhaps the authors could include this as a limitation / suggest as a future direction an expansion of this work to different age groups to ensure these mediators retain their relative contributions across the lifespan.

We thank the reviewer for this important comment. We do indeed observe a significant age effect on behavioral placebo analgesia (Table 2) and for the NPS results (Table 7), supporting the notion that age modulates the placebo effect in our sample. While the mean age across studies is indeed relatively young (26.3 years, weighted by sample size), the overall age range is relatively broad (min: 19, max: 59, 13.2% of participants older than 30 years), which makes us cautiously optimistic about the generalizability of our results. Nevertheless, we agree that the age distribution still imposes inherent limitations on the generalizability of these effects. To address this, we have added a supplementary analysis of age effects (Supplementary Figure S8, Supplementary Table S9) and a new paragraph in the Discussion acknowledging this limitation and highlighting it as an important direction for future research:

Page 24, Lines 626-633: *"Another limitation of our study is that the findings are based on a cohort of relatively young, healthy adults. As both brain function [9] and placebo effects [10] can change across the lifespan, the generalizability of these specific neural mediators requires further investigation. Indeed, our own results showed a significant effect of age on behavioral analgesia (β = -0.2898, T = -1.981, p = .048), as well as*

significant positive associations with placebo brain responses and age (Supplementary Figure S8, Supplementary Table S9). These findings underscore the need for future research systematically examining placebo mechanisms across different age groups, including patient populations affected by neurodegenerative diseases [11].”

For convenience, we present Supplementary Figure S8 detailing the results below:

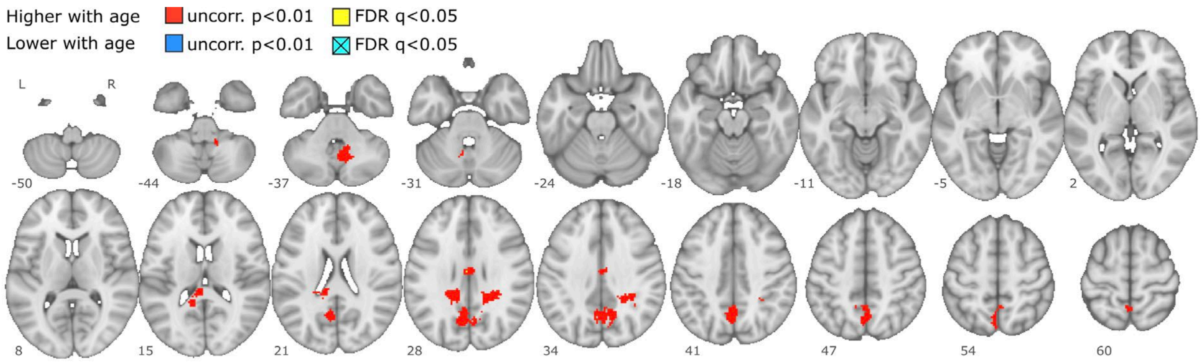

Supplementary Figure S8. *Association between placebo analgesia and age, adjusted for induction type, sex and control pain ratings We found multiple clusters where placebo-related brain activity showed a significant positive association with age in 6 different peak locations (FDR q < 0.05) in the right cerebellum, left precuneus, left and right posterior cingulate cortices and left middle cingulate cortex. In the case of uncorrected results (p < .01, red), we only show clusters that contain at least one voxel surviving false discovery rate correction (q < 0.05). No voxels showed negative association with age after FDR-correction. See* ***Supplementary Table S6*** *for a comprehensive list of activations significant after FDR correction.*

- An entire analysis (CA3) is devoted to investigating connectivity changes with Habenula but these results for INST / partial null findings in COND+INST are seemingly not discussed within the discussion – could the authors expand on their findings in this section about CA3 / include their thoughts on why this approach yielded an opposite effect than what would be expected given the other imaging findings – that is, greater deactivations within INST alone than with INST+COND?

We thank the reviewer for this observation. As also mentioned in our response to Reviewer #1, we have moved the habenula analysis to the supplementary materials, where we now provide an appropriate discussion of these findings.

Supplement, Page 12, Lines 124-137: *“The habenula has previously also been implicated in pain modulation and placebo mechanisms [2,3]. We obtained the habenula ROI from the subcortical brain atlas of [4] and divided it into bilateral components (by x = 0). Within all voxels of each ROI, we averaged the voxel-wise path a, path b and mediation (a*b) regression coefficients, as well as the group-mean estimates for INST and COND+INST (see Mediation Analysis in the main manuscript). To construct the null distribution for statistical inference, we used the ROI-wide averages of the voxel-wise null-distribution estimates, calculated based on sign-flipped 10 000 surrogate datasets, separately for path a, b, a*b and the INST and COND+INST group means. Similarly to the voxel-wise analysis, we calculated p-values by fitting a generalized Pareto distribution on the tail of the null distributions. We found that activity in the left habenula showed a significant placebo-related decrease with INST (harmonized effect size difference: -5.1%, p = .016) and COND+INST (-5.3%, p = .046), replicating our previous findings [2]. No statistically significant effects were observed in the right*

habenula. The ROI analysis also found no significant path a, path b, or mediation effects in either the left or right habenula."

Minor Comments:

- Suggested change in abstract 4th last line from: "yielded greater analgesia" to something which clearly defines this is a subjective change in perceived pain and not pharmacologically-driven analgesia – this is personal opinion, but the authors do use "subjective pain relief" in the introduction.

We thank the reviewer for this helpful suggestion to improve precision in our wording. We have revised the phrase on page 3, lines 101 to: *"yielded greater placebo analgesia".*

- Page 19. This may be outside the scope of this manuscript, but since the authors suggest Bayesian level encoding as partly responsible for the altered activity in pain-responsive regions such as dpIns and S1/2 – do the authors have access to the VAS ratings provided by participants during the conditioning process? Do they expect or can they measure statistically whether the average VAS difference between control- and placebo-treated sites during conditioning relates to the magnitude of either deactivation within these sites or across the NPS?

We thank the reviewer for this excellent suggestion. Unfortunately, we do not have access to VAS ratings from the conditioning phase for most of the included studies, as our dataset contains only the test phase data. We agree that examining whether the magnitude and variability of experienced pain relief during conditioning relates to subsequent neural deactivation patterns would provide valuable mechanistic insights into the Bayesian framework we propose. We have now added this as a suggestion for future research in our discussion:

Page 22, Lines 530-533:*" Future studies should also examine whether the magnitude and variability of pain relief experienced during the conditioning phase relates to the degree of subsequent neural deactivation, which would provide additional support for the Bayesian predictive coding framework proposed here [12]."*

- Page 20 – typo "…two regions that were also found to mediate the effects of induction method on placebo analgesia our study".

We thank the reviewer for catching this typo. We have corrected the sentence to read: *"...two regions that were also found to mediate the effects of induction method on placebo analgesia in our study".*

- Page 21 - If word count allows, could the authors expand their comment on VMPFC associations with chronic pain – it is interesting that the reference used explores the transitionary stages into chronic pain, alongside the increasing suggestion that components of chronic pain symptomatology are driven from encoded, learned behaviour – this line of arguments could be interesting to explore and dovetail nicely with the belief-updating role of the VMPFC proposed here.

We thank the reviewer for this insightful suggestion. We agree that the connection between VMPFC's role in belief updating and its involvement in chronic pain transitions

is fascinating and relevant to our findings. We have expanded our discussion of the VMPFC to include this perspective, highlighting how the belief-updating mechanisms we observe in placebo responses may also contribute to the learned components of chronic pain development.

Page 23, Lines 568-574: *"The VMPFC has long been associated with value-based processing [13,14], cognitive control [15], emotion regulation [16], and belief updating [17]. Importantly, alterations in VMPFC activity and connectivity have been reported in chronic pain [18], particularly during the transition from acute to chronic pain states. This connection is particularly relevant to our findings, as the belief-updating mechanisms we observe in placebo analgesia may represent similar neural processes that, when dysregulated, contribute to the learned behavioral components underlying chronic pain symptomatology."*

- Page 21 – typo "may compliments"
- Page 25 – I think this is a typo? "costume analysis code" – should this be "custom"?

Thank you for spotting these, we have corrected both typos.

Reviewer #2 (Remarks on code availability)

I have reviewed the code provided on Github, all seems to be present notwithstanding the 1st level contrast images which are noted to be available on request to the placebo imaging consortium. A readme is present, as well as basic installation instructions for use within python. Functions are provided within the manuscript itself and the preprint with extensive instructions for reproducibility.

We thank the reviewer for taking the time to review our code repository and for their positive feedback on its completeness and organization.

Reviewer #3 (Remarks to the Author)

This paper by Spisak et al. provides a meta-analysis of individual neuroimaging data, revealing distinct neural mechanisms for placebo analgesia induced by suggestions alone versus suggestions combined with conditioning.

We sincerely thank the reviewer for their thoughtful assessment of our manuscript. We greatly appreciate the constructive suggestions provided and have addressed each point in detail below. All changes made to the manuscript are cited accordingly (reviewer comments in blue, author replies in black, manuscript text in grey italics and new additions in green italics). Page and line numbers refer to the revised manuscript with tracked changes enabled.

While this study addresses an important question, the overall sample size in mega-analysis is small for functional magnetic resonance imaging (fMRI) data, and it is constrained by some significant methodological and interpretive limitations, such as including study heterogeneity, unverified analytical assumptions, and several unaddressed confounding factors. These concerns may compromise the robustness and clarity of the findings. The following are several major and minor concerns.

We thank the reviewer for their thorough evaluation and for the valuable comments that helped us further improve the quality of our analyses and strengthen the validity of our findings.

Regarding sample size, our total sample (*N* = 409) represents one of the largest placebo neuroimaging datasets to date. While recent recommendations [19,20] for between-subject brain-wide association studies (BWAS) rightly call for very large samples to detect small effects (e.g., <2% variance explained), these do not apply to our more powerful within-participant design or indeed to task fMRI, which was not tested in [19] and can yield much larger effect sizes. By analyzing within-subject contrasts, we account for a substantial proportion of inter-individual variance, ensuring that our study is well powered to detect the reported effects and that our findings are robust.

We acknowledge the reviewer's concerns about methodological limitations and confounding factors. Each of these points has been carefully addressed below, with corresponding revisions made to clarify and strengthen both the analysis and interpretation of our results.

Major concerns

(1) The meta-analysis may be subject to publication bias, as studies with significant placebo effects are more likely to be published. This could inflate the overall effect estimates.

We thank the reviewer for raising this important and valid point. Publication bias is an inherent risk in any meta-analysis, and we agree it is a factor to consider in studies that aggregate and draw conclusions from published results.

However, we believe several aspects of our approach mitigate this concern.

First, in our experience, neuroimaging studies are resource-intensive and are often published based on the quality of the data acquisition and analysis, even if the primary behavioral results are weak or null. This may make the field less susceptible to the classic "file-drawer problem" compared to research relying solely on less expensive behavioral outcomes. This is confirmed by the selection-bias assessment we performed during the revision, where we constructed funnel plots and assessed publication bias with two statistical tests (the conventional Egger-test, as well as the non-parametric Begg-test, due to the low number of studies), separately for the INST and COND+INST. We found no evidence of bias. We report these results on p. 6/7 in the revised manuscript, as well as in Supplementary Figure S11:

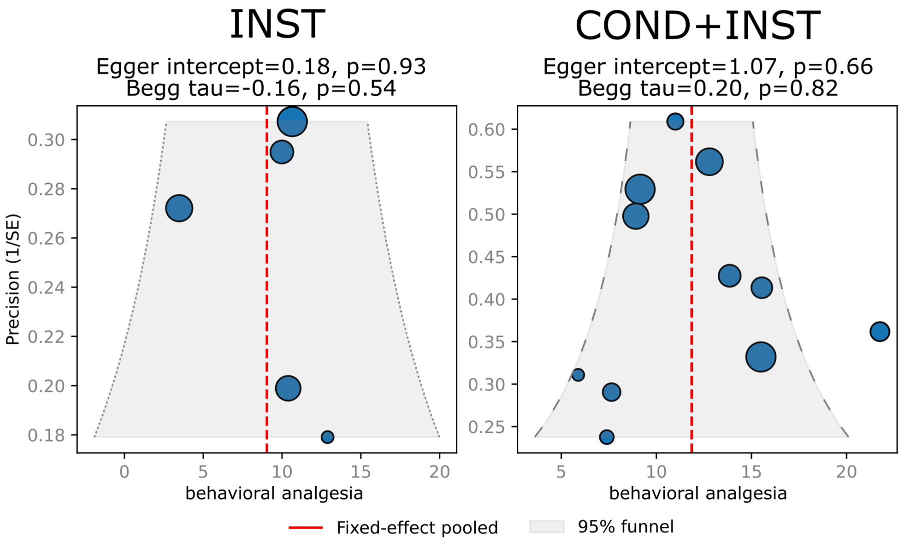

Supplementary Figure S11. *Funnel plots of behavioral analgesia by induction type (INST; COND+INST). Each point is a study with marker size representing study sample size. The x-axis shows behavioral analgesia (higher values = stronger behavioral placebo analgesia), the y-axis shows precision (inverse standard error). The red dashed line marks the fixed-effect pooled estimate; the shaded region is the 95% funnel. Egger's regression and Begg's rank-correlation tests indicate no evidence of funnel asymmetry in either group of studies (all p's > .05; see exact values in panel headers).*

Second, we included all participants from each shared dataset, no matter the strength of their individual behavioral placebo effects, which gives us substantial variability in responses from no to very strong placebo effects (also visible in Figure 2). Despite this variability, we find convincing evidence for brain activation differences, for example regarding differences in induction type.

Third, though some imaging findings in meta-analyses are likely to be subject to publication biases, as we and others have pointed out (e.g., amygdala activity in studies of negative emotion), here any potential bias in our analysis of instructions vs. conditioning + instructions is likely to work against our main findings. If, e.g., fewer studies of INST alone were published because they had weaker effects, that would bias the sample towards stronger effects in INST alone in surviving (published) studies, thus working against our findings of stronger placebo effects in COND+INST. It is therefore possible that conditioning effects are even stronger compared to INST than they appear here, though as we note above, our analyses do not reveal any evidence for publication bias.

While we cannot entirely rule out the influence of publication bias, we are confident that our findings provide a robust characterization of the neural correlates of placebo analgesia as it is reported in the published literature. To ensure full transparency, we have added more details about our comprehensive risk-of-bias assessment (adapted from our previous work [2,21]), including a complete flow chart of the study selection process (Supplementary Figure S5), as well as a detailed description of our data acquisition procedure and the formal risk of bias assessment (Supplementary Methods 1-3 and Supplementary Tables S2-3). The results of this assessment are now summarized in the Results section and Supplement:

Page 6/7, Lines 186-192: *"We performed a dedicated risk-of-bias assessment with the Cochrane risk of bias tool [1], evaluating the risk for selection, performance, detection, attrition and reporting and sequence biases. Our assessment did not find evidence for publication bias (see Supplementary Figure S11 and concluded that, in terms of behavioral and neural differences between the induction types, the risks of these biases are generally low, although they cannot be fully ruled out. For details see Supplementary Methods 1-3, Supplementary Tables S2-3 and Supplementary Figure S5."*

Supplement, Page 19, Lines 298-306: *"We assessed small-study effects/publication bias using funnel plots of study-specific behavioral analgesia estimates (effect size on the x-axis) against their precision (1/SE). For visualization, we overlaid the fixed effect pooled estimate with its 95% funnel and scaled point size by sample size. Funnel asymmetry was tested using Egger's regression [22] (OLS of effect/SE on precision with an intercept; two sided test of intercept = 0) and Begg & Mazumdar's rank correlation test [23] (Kendall's tau between effects and SEs). Analyses were performed separately for the INST and COND+INST subgroups, using α = 0.05. We found no evidence of publication bias. See **Supplementary Figure S11** for details."*

Finally, we have added a sentence to our limitations section to explicitly acknowledge this potential issue:

Page 25, Lines 653-660: *"Like any meta-analysis of published literature, our results could be influenced by publication bias, wherein studies with significant findings are more likely to be published. While no evidence of publication bias was detected in our dataset (see Supplementary Figure S11, we cannot entirely rule out its presence. However, our primary aim is not to estimate the absolute effect size of placebo analgesia across studies, but to elucidate the neural mechanisms underlying the different induction techniques, given that the placebo effect has been successfully induced. Studies without a behavioral effect, while important for estimating overall prevalence, would be uninformative for our specific research question."*

For more information on our risk of bias assessment, see also our response on your comment (4) below.

(2) The conditioning protocols varied considerably across studies in terms of duration, intensity, and reinforcement procedures. This heterogeneity may have influenced the results and the authors can clarify whether it was accounted for in the analysis or interpretation.

We thank the reviewer for raising this important point. As these variations in conditioning protocols occur between, rather than within, studies, they are inherently captured by the study covariate included in our model, which accounts for variance arising from protocol differences, site effects, and other study-specific factors. Furthermore, we argue that this heterogeneity may, to some extent, enhance the generalizability of our findings. By identifying a common neural signature of conditioned placebo analgesia despite variations in specific parameters, our results point to a core, robust mechanisms underlying these effects.

However, we fully agree that disentangling the specific contribution of individual parameter (e.g., duration, intensity, reinforcement parameters) would be valuable. This was beyond the scope of the present study, as the number of studies systematically varying these parameters was insufficient to enable a meaningful analysis. We have now added a paragraph to our Limitations section to discuss this explicitly, referring to individual studies to illustrate these issues:

Page 25, Lines 640-652: *"Heterogeneity across the studies included in this meta-analysis extends beyond stimulation laterality, encompassing differences in duration, intensity, and conditioning protocols and includes diverse pain stimuli. While identifying consistent neural effects across these varied methodologies strengthens the generalizability of our core findings, this heterogeneity is also a source of variance. We have accounted for this in our statistical models by including study-level covariates, which absorb the variance attributable to these protocol differences along with other site-specific effects. Furthermore, we assessed the covariance between induction type and these methodological variables and found no evidence for significant associations (see Supplementary Figure S10 and Supplementary Table S13 for details). However, we cannot fully exclude the possibility that residual confounding may have contributed to our results, warranting cautious interpretation. Fully disentangling the specific influence of such methodological parameters would require a different, more targeted meta-analytic approach, and importantly, more neuroimaging studies that systematically vary these parameters."*

(3) Multiple claims of stronger associations are based on p-values instead of coefficients. For example, the Figure 6 caption.

We thank the reviewer for this valuable suggestion. We have now thoroughly revised the manuscript to ensure that we consistently report effect sizes (e.g., correlation coefficients, beta values, or standardized effect sizes) alongside *p*-values to provide comprehensive evidence for our claims. For instance, the caption of Figure 6 now reads as follows:

Page 19, Lines 446-456: *"Figure 6. NPS, but not SIIPS, more strongly predicts behavioral analgesia induced by COND+INST than by INST alone. The scatter plots show that the relationship between analgesia and NPS response was significantly stronger for COND+INST (orange; r = -.19, p = .002) than for INST-alone (blue; r = -.10, p = .12). The difference in these associations was confirmed by a significant interaction effect (black; T(409) = -1.83, p = .034). No such interaction was observed for the SIIPS (COND+INST: r = -.15, p = .05; INST: r = -.16, p = .02, interaction: T(390) = -1.17, p = .14)* ~~*the association between behavioral placebo analgesia and the NPS*~~

~~and SIIPS signature responses, respectively.~~ Regression lines and 95% confidence intervals are plotted separately for the INST and COND+INST induction techniques. Blue and orange p-values correspond to the INST and COND+INST induction techniques, corrected for age and sex. Black p-values correspond to the interaction effect between induction technique and behavioral analgesia; with the significant p-value for NPS indicating that the association between the NPS and behavioral analgesia is significantly stronger in case of COND+INST. Glass brains represent the predictive weights of the signatures (red: positive weight, blue: negative weight).

(4) Selection bias is not addressed in the manuscript. Since the authors collected data by contacting investigators from prior studies, it would be helpful to report additional details, such as the number of studies excluded due to unavailable data and the number of authors contacted versus those who responded, to assess the risk of inclusion bias.

We thank the reviewer for raising this important point about transparency in our study selection process. In the revised manuscript we now provide a complete flow chart of the study selection process (Supplementary Figure S5 including the number of studies identified, authors contacted, datasets received, and the specific reasons for excluding studies at each stage), as well as a detailed description of our data acquisition procedure and a formal risk of bias assessment (Supplementary Methods 1-3, Supplementary Figure S11 and Supplementary Tables S2-3, including selection, performance, and reporting biases), adapted from our previous publications [2,21]. In light of the comprehensive analyses presented, we are confident that the potential biases identified do not affect the robustness or validity of our conclusions (please also see our response to your comment (1) concerning publication bias).

Furthermore, now we provide a more detailed description of these procedures in the Methods section:

Page 27, Lines 708-714: *"Our original data acquisition procedure [2,21] identified 96 published articles, of which 28 were selected as eligible according to above criteria a-e. Authors of 20 studies provided data (including 71% of eligible studies and 79.4% of eligible participants). In the present analysis, we excluded studies with a between-participant design (according to criteria f and g), leading to a final dataset consisting of k = 16 studies and n = 415 individuals (n = 409 after excluding participants with missing pain ratings). Details on data acquisition are provided in Supplementary Methods 1-2, Supplementary Table S2 and Supplementary Figure S5 as well as in [2,21]."*

Page 28, Lines 724-730: *"**Risk of Bias Assessment**. We used the Cochrane risk of bias tool [1] to evaluate the risk of bias for studies included in the present meta-analysis (details are in Supplementary Methods 3 and Supplementary Table S3. We assessed biases from selection (which arises via insufficient randomization), performance (via insufficient blinding of participants or treatment providers), detection (via insufficient blinding of analysts), attrition (by missing data), reporting (via underreporting of nonsignificant studies), and sequence (which is potentially introduced by within-participant designs)."*

(5) The conjunction analysis is appropriate for identifying core regions commonly engaged across both induction methods, but its statistical conservativeness may overlook meaningful activation patterns present in only one condition or marginally in both. The authors may consider complementary approaches (e.g., inclusive masking or direct contrasts) to provide a fuller picture.

We thank the reviewer for this excellent methodological suggestion. We agree that conjunction analysis, while robust for identifying core overlapping activations, is statistically conservative and may overlook areas with weaker or sub-threshold effects in one condition.

Following the reviewer's recommendation, we have now assessed the average response in all participants regardless of induction type (direct contrast), as well as the union of significant voxels (inclusive masking). The direct contrast confirmed the same core regions identified by the original analyses but also revealed several additional areas of common engagement that did not survive the strict conjunction threshold, including the inferior and middle temporal gyri, visual cortex, and hippocampus.

We have now added these new findings to the manuscript, including Supplementary Figure S6 and Supplementary Table S6 to display the results. We also briefly refer to this new analysis in the main text:

Page 9, Lines 276-283: *"While the conjunction analysis is appropriate for identifying core regions commonly engaged across both induction methods, its statistical conservativeness may overlook meaningful activation patterns present in only one condition or marginally in both. Therefore, in a supplementary analysis (Supplementary Figure S6 and Supplementary Table S6), we have evaluated the average response in all participants regardless of induction type (direct contrast), as well as the union of significant voxels (inclusive masking). This analysis largely corroborated our previously published results [2] based on the restricted dataset used in the present study."*

A Pooled mean placebo response (both INST & COND+INST)

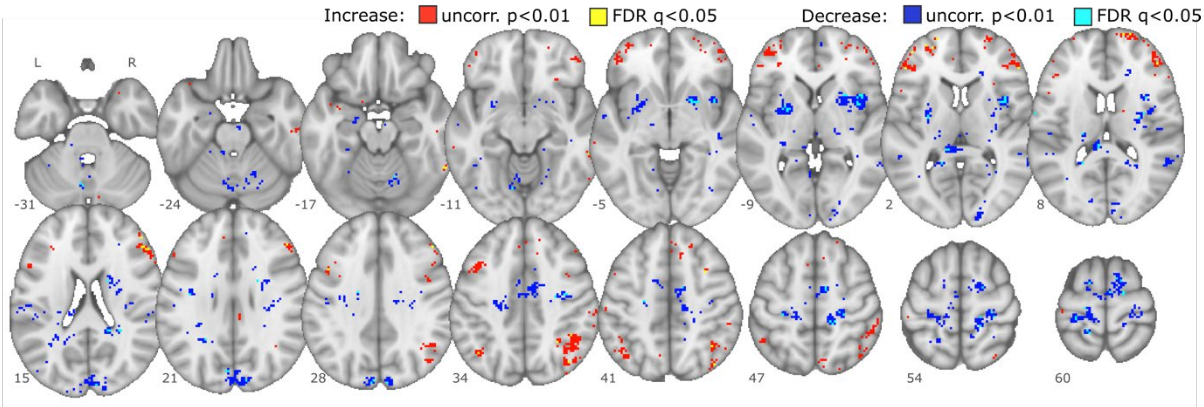

B INST or COND+INST (inclusive masking)

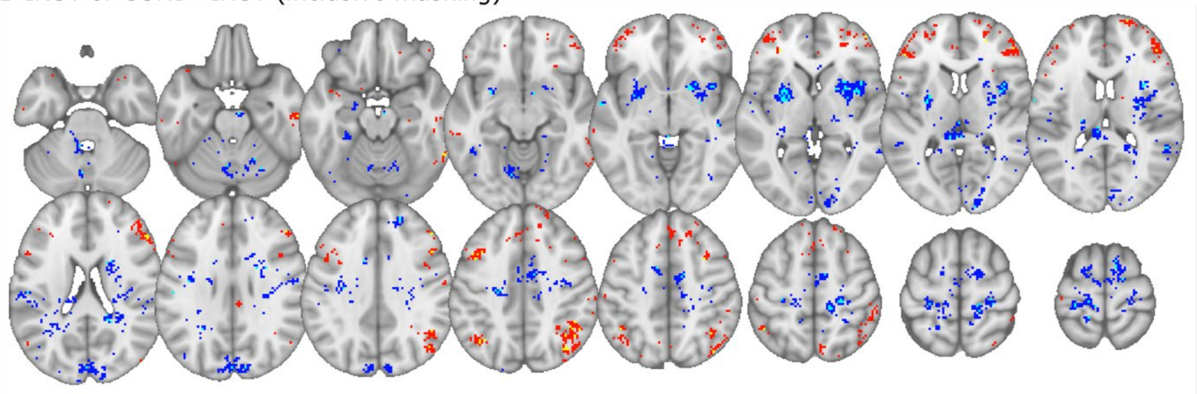

Supplementary Figure S6. Group-level brain activation for placebo analgesia. A: Pooled across all participants (regardless of induction type). B: The union of INST and COND+INST (inclusive masking). All results are adjusted for age, sex and control pain ratings. Images are shown in neurological convention (left is left). Standard-space Z-coordinates are shown for each slice on the first panel. The second panel visualizes the same slices. See Supplementary Table S6 for a comprehensive list of activations significant after FDR correction.

(6) The sample sizes across included studies vary substantially, from as few as n=10 to over n=40. It is unclear whether sample size weighting or sensitivity analyses were applied to reduce potential bias from small-sample studies in group-level statistics (e.g., conjunction analysis, mediation). Without such adjustments, small studies could exert disproportionate influence on the results. In addition, the sample size is small for fMRI studies, and it has been demonstrated that replication of fMRI findings may need much larger sample size.

We thank the reviewer for raising these methodological aspects and would like to clarify two aspects of our design that ensure its robustness.

First, regarding variable sample sizes, our Individual Participant Data (IPD) meta-analysis pools all participants into a single model. This approach inherently weights each study by its sample size, which is the standard and appropriate way to avoid the potential biases from small studies in traditional meta-analyses.

Second, with respect to the reviewer's comment on sample size requirements for replicability, as noted in our first response above, recent recommendations [19,20] for between-subject brain-wide association studies (BWAS) do not apply to our more

powerful within-participant design, ensuring sufficient power to detect the reported effects and supporting the robustness of our findings.

(7) The studies included diverse pain stimuli (such as heat, laser, and distension), which likely engage different neural pathways and may influence placebo responses. The authors should consider its potential as a confounding factor.

We thank the reviewer for raising this important point regarding the heterogeneity of pain stimuli.

First, from a methodological standpoint, our analytical approach already partly accounts for this potential confound. For a variable such as stimulus type (e.g., heat, laser, distension) to act as a confound, it must covary with the effect of interest. Placebo vs. control comparisons are conducted within studies and are therefore matched on study-level variables such as stimulus type, eliminating meaningful risk of confounding. In contrast, the COND+INST vs. INST comparison involves different groups of studies that could vary in methodological variables, including stimulus type. To address this, we explicitly assessed the covariance between this effect and methodological variables. The revised manuscript now details this analysis and its results:

Page 29, Lines 772-777: *"As COND+INST vs. INST compares different groups of studies that could vary in methodological variables like stimulus type, we assessed the covariance between induction type and various methodological variables like the type, location and duration of pain stimulation or the repetition and echo times of the respective fMRI sequences. We found no significant association with any of these variables (see Supplementary Figure S10 and Supplementary Table S13 for details)."*

Second, from a theoretical standpoint, we argue that this heterogeneity is a major strength that enhances the generalizability of our findings. By identifying a consistent neural signature for placebo effects across somatic and visceral pain, and across heat, laser, and mechanical stimulation, our results point to a core, modality-independent mechanism of top-down pain modulation. This suggests that the brain engages a common set of higher-order belief- and expectation-related processes, regardless of the specific nature of the afferent nociceptive input.

We have now addressed the above aspects in more detail in the limitations section of our discussion:

Page 25, Lines 640-652: *"Heterogeneity across the studies included in this meta-analysis extends beyond stimulation laterality, encompassing differences in duration, intensity, and conditioning protocols and includes diverse pain stimuli. While identifying consistent neural effects across these varied methodologies strengthens the generalizability of our core findings, this heterogeneity is also a source of variance. We have accounted for this in our statistical models by including study-level covariates, which absorb the variance attributable to these protocol differences along with other site-specific effects. Furthermore, we assessed the covariance between induction type and these methodological variables and found no evidence for significant associations (see Supplementary Figure S10 and Supplementary Table S13 for details). However, we cannot fully exclude the possibility that residual*

confounding may have contributed to our results, warranting cautious interpretation. Fully disentangling the specific influence of such methodological parameters would require a different, more targeted meta-analytic approach, and importantly, more neuroimaging studies that systematically vary these parameters."

(8) In Figure 4, why in caudate, the correlation seems to be positive (red), but the Pearson correlation r is negative?

We are thankful to the reviewer for spotting this discrepancy in Figure 4 and apologize for the confusion. The correlation for the caudate is indeed positive (r = .15), as correctly stated in Table 5 and as indicated by the positive slope of the regression line and red color coding in the figure itself. We have corrected this typo in the revised version of Figure 4.

(9) The assumptions underlying the mediation analysis, such as the absence of unmeasured confounding between the mediator and the outcome, are not discussed or evaluated. It would strengthen the study to explicitly address these assumptions and their potential impact on interpretation.

We thank the reviewer for raising this important point about the interpretation of our mediation analysis. Following this suggestion, we have now added a paragraph to our Limitations section to clarify that mediation analysis is used here as a powerful statistical tool for identifying plausible neural pathways and testing for significant relationships, rather than to make definitive causal claims:

Page 25, Lines 661-674: *"Finally, our use of mediation analysis entails important interpretive caveats. While this analysis provides a powerful framework for identifying potential neural pathways linking induction type to analgesia, it does not establish causality for paths linking observed variables (as opposed to experimentally randomized variables, which afford stronger causal interpretation* [24]*). A key assumption for making causal claims is the absence of unmeasured confounding between the mediator (here, brain activity) and the outcome (here, behavioral analgesia). In a complex psycho-biological context such as this, it is impossible to rule out the influence of all potential unmeasured confounders, such as stable personality traits or prior beliefs not captured in our model. Therefore, we do not make directional causal claims about the observed associations between regional brain activity and analgesia. However, because placebo vs. control conditions were randomly assigned, it is reasonable to infer that placebo effects on brain activity are causal. Our mediation results, which depend jointly on both effects, should thus be interpreted as identifying statistically significant pathways consistent with a proposed mechanistic model, highlighting promising targets for future studies explicitly designed to test for causal directionality."*

Minor Concerns

(1) The methods section does not clearly specify the statistical software or packages used for key analyses such as the mixed-effects model (CA1) or Levene's test. Additionally, it is unclear which estimation method was used to compute effect sizes (e.g., REML vs. ML) in the mixed model.

We have now updated the Methods section (under CA1: Variance Analysis) to specify the software packages and estimation methods used:

Page 30, Lines 814-816: *"The mixed-effects model was implemented using the using the statsmodels package (v0.14.2) in Python with Restricted Maximum Likelihood (REML) estimation, and Levene's test was conducted using the scipy.stats package (v1.13.1)."*

(2) Although the 3-point difference in VAS ratings between COND+INST and INST is statistically significant, prior research suggests that a minimal clinically important difference (MCID) is typically around 10–15 points on a 100-point scale. The authors should distinguish between statistical and clinical significance and acknowledge this limitation in the discussion.

We thank the reviewer for this important perspective on the clinical relevance of our findings, which we agree warrants further clarification. We have added a paragraph to our discussion to explicitly address this point and contextualize our findings:

Page 21, Lines 483-491: *"It is important to place the magnitude of our behavioral findings in the appropriate context. The observed VAS increase in analgesia with conditioning, while statistically robust, is below thresholds typically considered a minimal clinically important difference (MCID) in pain research involving patients (e.g. [25,26]). This should be interpreted in light of our study's mechanistic goal and inclusive analytical approach. We analyzed data from all participants, rather than pre-selecting for 'placebo responders', which necessarily lowers the average group effect size. Our findings therefore reflect the average benefit of adding conditioning across a heterogeneous population of healthy volunteers in an experimental setting, rather than the potential effect size in a clinical population or a responder subgroup."*

(3) Line 276-277, (Figure 3B,). Need to clean up.

We have corrected the Word formatting glitch that incorrectly referenced the entire Table 4 within the caption of Figure 3 instead of its label.

(4) Correct the citation format. "A detailed description of the data collection procedures can be found in 14" (Line 603)

We corrected the citation format on page 27, line 695 to *"Zunhammer et al. (2018) [21]"*.

Reviewer #4 (Remarks to the Author)

I co-reviewed this manuscript with one of the reviewers who provided the listed reports. This is part of the Nature Communications initiative to facilitate training in peer review and to provide appropriate recognition for Early Career Researchers who co-review manuscripts.

We are thankful to Reviewer #4 for contributing to the assessment of our manuscript.

Mentioned references

1. The Cochrane Collaboration. *Cochrane Handbook for Systematic Reviews of Interventions Version 5.1.0 [updated March 2011]*. (2011).
2. Zunhammer, M., Spisak, T., Wager, T. D., Bingel, U. & The Placebo Imaging Consortium. Meta-analysis of neural systems underlying placebo analgesia from individual participant fMRI data. *Nat. Commun.* **12**, 1391 (2021).
3. Brooks, J. & Tracey, I. From nociception to pain perception: imaging the spinal and supraspinal pathways. *J. Anat.* **207**, 19–33 (2005).
4. Pauli, W. M., Nili, A. N. & Tyszka, J. M. A high-resolution probabilistic in vivo atlas of human subcortical brain nuclei. *Sci. Data* **5**, 180063 (2018).
5. Keogh, E. Sex, gender, and pain: Evidence and knowledge gaps. *Curr. Opin. Psychol.* **63**, 102006 (2025).
6. Shafir, R., Olson, E. & Colloca, L. The neglect of sex: A call to action for including sex as a biological variable in placebo and nocebo research. *Contemp. Clin. Trials* **116**, 106734 (2022).
7. Brügger, M. *et al.* Taking Sides with Pain – Lateralization aspects Related to Cerebral Processing of Dental Pain. *Front. Hum. Neurosci.* **volume 5**-, (2011).
8. Symonds, L. L., Gordon, N. S., Bixby, J. C. & Mande, M. M. Right-lateralized pain processing in the human cortex: An fMRI study. *J. Neurophysiol.* **95**, 3823–3830 (2006).
9. Setton, R. *et al.* Age differences in the functional architecture of the human brain. *Cereb. Cortex* **33**, 114–134 (2023).
10. Weimer, K., Colloca, L. & Enck, P. Age and Sex as Moderators of the Placebo Response - An Evaluation of Systematic Reviews and Meta-Analyses across Medicine. *Gerontology* **61**, 97–108 (2014).
11. Wiech, K. & Bingel, U. Alzheimer disease may compromise patients' ability for expectancy-based pain modulation. Now what? *Pain* **165**, 256–257 (2024).
12. Seymour, B. & Mancini, F. Hierarchical models of pain: Inference, information-seeking, and adaptive control. *Neuroimage* **222**, 117212 (2020).
13. Roy, M. *et al.* Representation of aversive prediction errors in the human periaqueductal gray. *Nat. Neurosci.* **17**, 1607–1612 (2014).
14. Veselic, S. *et al.* A cognitive map for value-guided choice in the ventromedial prefrontal cortex. *Cell* **188**, 3259-3273.e22 (2025).
15. Clairis, N. & Pessiglione, M. Value Estimation versus Effort Mobilization: A General Dissociation between Ventromedial and Dorsomedial Prefrontal Cortex. *J. Neurosci.* **44**, 1–13 (2024).
16. Koban, L., Jepma, M., Geuter, S. & Wager, T. D. What's in a word? How instructions, suggestions, and social information change pain and emotion. *Neurosci. Biobehav. Rev.* **81**, 29–42 (2017).
17. Motzkin, J. C. *et al.* Human ventromedial prefrontal cortex lesions enhance the effect of expectations on pain perception. *Cortex* **166**, 188–206 (2023).
18. Löffler, M. *et al.* Corticostriatal circuits in the transition to chronic back pain: The predictive role of reward learning. *Cell Reports Med.* **3**, (2022).
19. Marek, S. *et al.* Reproducible brain-wide association studies require thousands of individuals. *Nature* **603**, 654–660 (2022).
20. Spisak, T., Bingel, U. & Wager, T. D. Multivariate BWAS can be replicable with moderate sample sizes. *Nature* **615**, E4-e7 (2023).
21. Zunhammer, M., Bingel, U. & Wager, T. D. Placebo effects on the neurologic pain signature: A meta-analysis of individual participant functional magnetic

resonance imaging data. *JAMA Neurol.* 1–10 (2018) doi:10.1001/jamaneurol.2018.2017.
22. Egger, M., Davey Smith, G., Schneider, M. & Minder, C. Bias in meta-analysis detected by a simple, graphical test. *BMJ* **315**, 629–634 (1997).
23. Begg, C. B. & Mazumdar, M. Operating characteristics of a rank correlation test for publication bias. *Biometrics* **50**, 1088–1101 (1994).
24. Mascha, E. J., Dalton, J. E., Kurz, A. & Saager, L. Understanding the Mechanism: Mediation Analysis in Randomized and Nonrandomized Studies. *Anesth. Analg.* **117**, (2013).
25. Myles, P. S. *et al.* Measuring acute postoperative pain using the visual analog scale: the minimal clinically important difference and patient acceptable symptom state. *BJA Br. J. Anaesth.* **118**, 424–429 (2017).
26. Olsen, M. F. *et al.* Pain relief that matters to patients: systematic review of empirical studies assessing the minimum clinically important difference in acute pain. *BMC Med.* **15**, 35 (2017).

Rebuttal Letter

Reviewer #1 (Remarks to the Author):

Through the revisions of the manuscript, all of my and other reviewers' comments appear to be addressed very well.

Reply: Thank you for your positive evaluation and effort to review our work.

Reviewer #2 (Remarks to the Author):

The authors have resolved all of my comments with specific analyses and discussion elements included which do not require further adjustments in my opinion.
The reordering of habenula analysis to supplement also improves manuscript structure and flow.
Compliments to the authors for this work - which is a lovely addition to our field
Dr Lewis Crawford

Reviewer #2 (Remarks on code availability):

Code assessed in first revision cycle. No outstanding issues

Reply: Thank you for your positive evaluation and effort to review our work.

Reviewer #3 (Remarks to the Author):

I appreciate the authors' thorough revisions, which have addressed most of my concerns.

I recognize that this study likely represents the largest dataset assembled to date for this type of placebo neuroimaging meta-analysis. That said, given the substantial heterogeneity across the included studies, it remains unclear how this variability may affect effective power and the robustness of the pooled estimates.

To help readers interpret the strength of the evidence, it would be valuable to add a formal power/sensitivity analysis (e.g., minimal detectable effect sizes) demonstrating what effect sizes are detectable with the current sample size under plausible assumptions for a within-participant, task-based fMRI design, and clarifying any remaining risks to power and inference.

Reply: Thank you for your positive evaluation and effort to review our work. To address the reviewer's concern about heterogeneity and robustness of pooled estimates, we added a formal

sensitivity analysis that derives voxel-wise minimal detectable effects (MDEs) directly from the sign-flip null distributions used for inference. For two-sided significance level $\alpha < .01$ (the cluster-forming threshold used to visualize our results), the median MDE across all brain voxels was $\beta = 6.5\%$ in terms of percentile points for Path A (on the effect-size scale used in the manuscript), $r = .131$ for Path B (correlation), and a×b = 0.249 for the indirect (mediation) effect. In the manuscript tables 1-3 reporting $p < .01$ clusters with FDR-significant peaks, the corresponding absolute peak effects ranged from $\beta = 5.4\text{-}10.7$, $|r| = .11\text{-}.18$, and a×b = 0.2-0.6, robustly exceeding the corresponding detectability thresholds and including peaks detectable even at $\alpha = .001$. Detectability thresholds were remarkably homogenous across brain voxels and inference paths, with slightly higher thresholds in cerebellar and orbitofrontal regions (overall maximal MDE values: $\beta = 13.0\%$, $r = .17$, a×b = 0.68), suggesting that in certain parts of these regions, our study may have missed small effects. For further details, see Supplementary Figure S12 (reproduced below for your convenience).

In the manuscript, we discuss this analysis as follows (page 12, lines 332-337):

"To clarify any remaining risks to power and inference, we quantified voxel-wise minimal detectable effects (MDEs) for each of the three inference paths by deriving detectability thresholds from the corresponding sign-flip null distributions. The resulting MDE maps, shown in Supplementary Figure S12, indicate high sensitivity across most brain voxels, with reduced detectability largely confined to peripheral cerebellar and orbitofrontal regions, consistent with susceptibility-related fMRI signal dropout."

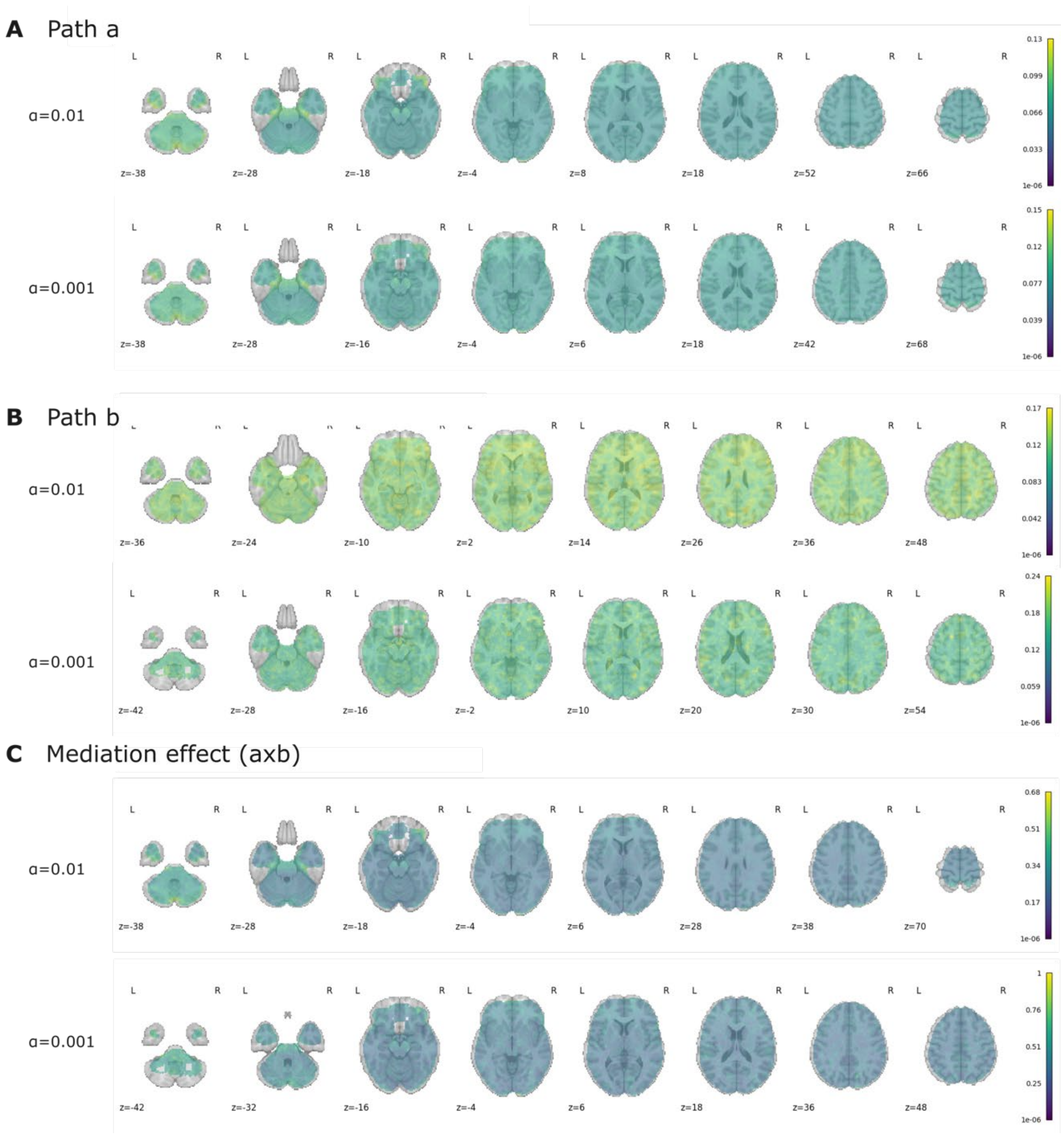

Supplementary Figure S12. *Voxel-wise minimal detectable effects (MDEs) for the mediation analysis paths. (A) Path a (X→M): induction type effect (COND+INST vs. INST) on quantile-harmonized placebo brain responses; MDE is shown in the harmonized quantile units (approximately a percentage-point shift in the proportion of participants with positive placebo responses; (B) Path b (M→Y): association between harmonized brain responses and behavioral placebo analgesia; MDE is shown as partial correlation r after residualizing the same covariates as in the main model. (C) Indirect (a×b): mediation (ACME) effect; MDE is shown in VAS points. For each voxel and path, MDE at level* α *was defined as the two-sided detectability threshold* $MDE_v(\alpha) = Q_{1-\alpha}(|E_{null,v}|)$, *i.e., the 1-α quantile of the absolute effect under the sign-flip null distribution (1,000 sign-flip samples), generated by randomly flipping the sign of participant placebo–control contrast maps and rerunning the full harmonization and mediation model (including study regressors orthogonalized to induction type and covariates age, sex, and control pain). Axial slices (z-coordinates shown) display MDE maps for* α *= .01, and .001 (rows); lower MDE values indicate higher sensitivity. To clarify any remaining risks to power and inference, we quantified voxel-wise MDEs for each inference path from the corresponding sign-flip null distributions. For two-sided significance level p < .01 (the cluster-forming threshold used to visualize our results), the median MDE across all brain voxels was β = 6.5% in terms of percentile points for Path A (on the effect-size scale used in the manuscript), r = .131 for Path B (correlation), and a×b = 0.249 for the indirect (mediation) effect. In the manuscript tables X-Y reporting p < .01 clusters with FDR-significant peaks,*

the corresponding absolute peak effects ranged from β = 5.4-10.7, |r| = .11-.18, and a×b = 0.2-0.6, robustly exceeding the corresponding detectability thresholds and including peaks detectable even at α = .01 and α = .001. Detectability thresholds were remarkably homogenous across brain voxels and inference paths, with slightly higher thresholds in cerebellar and orbitofrontal regions (overall maximal MDE values: β = 13.0%, r = .17, a×b = 0.68), suggesting that in certain parts of these regions, our study may have missed small effects. In sum, the resulting MDE maps indicate high sensitivity across most brain voxels, with reduced detectability largely confined to peripheral cerebellar and orbitofrontal regions, consistent with susceptibility-related fMRI signal dropout.

Reviewer #4 (Remarks to the Author):

I co-reviewed this manuscript with one of the reviewers who provided the listed reports. This is part of the Nature Communications initiative to facilitate training in peer review and to provide appropriate recognition for Early Career Researchers who co-review manuscripts.

Reply: Thank you for your positive evaluation and effort to review our work.
